# Genomic insights into the broad antifungal activity, plant-probiotic properties, and their regulation, in *Pseudomonas donghuensis* strain SVBP6

**DOI:** 10.1371/journal.pone.0194088

**Published:** 2018-03-14

**Authors:** Betina Cecilia Agaras, Andrés Iriarte, Claudio Fabián Valverde

**Affiliations:** 1 Laboratorio de Bioquímica, Microbiología e Interacciones Biológicas en el Suelo, Departamento de Ciencia y Tecnología, Universidad Nacional de Quilmes, Bernal, Buenos Aires, Argentina; 2 Consejo Nacional de Investigaciones Científicas y Técnicas (CONICET), Buenos Aires, Argentina; 3 Laboratorio de Biología Computacional, Departamento de Desarrollo Biotecnológico, Instituto de Higiene, Facultad de Medicina, Universidad de la República, Montevideo, Uruguay; Universite Paris-Sud, FRANCE

## Abstract

Plant-growth promotion has been linked to the *Pseudomonas* genus since the beginning of this research field. In this work, we mined the genome of an Argentinean isolate of the recently described species *P*. *donghuensis*. Strain SVBP6, isolated from bulk soil of an agricultural plot, showed a broad antifungal activity and several other plant-probiotic activities. As this species has been recently described, and it seems like some plant-growth promoting (PGP) traits do not belong to the classical pseudomonads toolbox, we decide to explore the SVBP6 genome via an bioinformatic approach. Genome inspection confirmed our previous *in vitro* results about genes involved in several probiotic activities. Other genetic traits possibly involved in survival of SVBP6 in highly competitive environments, such as rhizospheres, were found. Tn*5* mutagenesis revealed that the antifungal activity against the soil pathogen *Macrophomina phaseolina* was dependent on a functional *gacS* gene, from the regulatory cascade Gac-Rsm, but it was not due to volatile compounds. Altogether, our genomic analyses and *in vitro* tests allowed the phylogenetic assignment and provided the first insights into probiotic properties of the first *P*. *donghuensis* isolate from the Americas.

## Introduction

Among all soil bacterial genera having a representative described as a Plant-Growth Promoting Microbe (PGPM), the genus *Pseudomonas* comprise a wide variety of probiotic species, with distinct mechanisms of action that strengthen plant health, either directly or indirectly [1,2]. Several pseudomonads have demonstrated production of different kinds of secondary metabolites involved in antagonism to pathogens, phytostimulation or nutrient supply, and an ability to degrade complex organic compounds, not only being able hence to contribute to plant growth, but also to bioremediation of soils [2–6]. As members of the γ-Proteobacteria subphylum, which range from 1% to 34% of the abundance of total soil bacterial community of different environments [7], *Pseudomonas* spp. are key members of the soil microbiome. Considered as copiotrophs, because they are specially present in areas where resource availability is high and carbon sources are simple [8], their remarkable nutritional versatility allows them to exploit diverse rhizosphere environments, where each plant exudates a variety of organic compounds [9]. Their high rhizosphere competence is critical for the correct functioning of microbial inoculants and their effect on soil microbiome [10,11].

The physiological and genetic adaptability of *Pseudomonas* spp. facilitated the widespread distribution of this genus in various ecosystems around the world [12–14]. Besides, those genomic traits allow these bacteria to be continuously studied as sources of new metabolic pathways and novel products, sometimes related with plant-probiotic activities [15–18], which could be useful for the development of more efficient agricultural bio-inputs.

Genome sequencing has become a useful tool for different microbiology projects, not only to understand the genetic basis of metabolic processes [19,20], but also to unravel some inconsistences in taxonomic affiliations that the classical approaches cannot define, for instance, by comparing the average nucleotide identity (ANI) [21,22]. As *Pseudomonas* spp. genomes are so adaptable, these techniques have recently allowed the description of several novel species [23–26], and to better organize this genus, which is the largest within Gram-negative bacteria [27]. An additional benefit of next generation sequencing is the possibility to access to an increasing amount of genetic information. Thus, genome mining has become an essential tool for processing such amount of data generated, in order to search for new catalysts, targets or products, particularly in microorganisms with industrial applications, as PGPM [28].

There are several reports on the difficulties to reproduce the positive effects of PGPM that had been seen at the laboratory on plant growth, in field assays, most probably due to the influence of several uncontrolled biotic and abiotic factors [29–33]. Therefore, the focus has been recently put into the isolation of PGPM from the same agricultural plots or crop rhizospheres where they would be applied later as inoculants. This strategy may help to overcome difficulties in adapting non-native bacterial species in a new environment [6,34–37]. With this purpose, we isolated and characterized a group of 19 pseudomonads from the humid Pampean region of Argentina, which were selected by their *in vitro* antagonistic activity against different fungal pathogens [6]. One of the isolates, named SVBP6, displayed a broad spectrum of fungal-growth inhibition activity. Besides, SVBP6 could not be clearly assigned to any of the *Pseudomonas* species already described at that moment. Therefore, the aim of this work was to obtain and explore the draft genome of strain SVBP6, in order to better define its taxonomic assignment, to survey the genetic determinants of the broad antifungal activity shown *in vitro*, and to discover putative plant-probiotic traits that may be instrumental for this strain as an agricultural bio-input.

## Materials and methods

### Isolation of SVBP6, and physiological and biochemical characterization

Strain SVBP6 was isolated from the bulk top soil (0–10 cm) of an agricultural plot located in Viale, Entre Ríos province, Argentina (31° 52’ 59,6” S; 59° 40’ 07” W) using Gould’s S1 selective media [38]. At sampling time (February 2010), the plot was cultivated with soybean and had a history of no-till management under good agricultural practices (crop rotation, nutrient replacement, and minimized agrochemical use) for, at least, 13 years [39]. Isolate SVBP6 was selected by its antagonistic potential against a diverse group of phytopathogenic fungi that included members of *Fusarium*, *Colletotrichum*, *Phomopsis*, *Macrophomina* and *Cercospora* genera [6].

Colony morphology was observed under a magnifying glass (Olympus SZ61, 10×), after sowing a 20 μl-drop of a bacterial suspension with DO_600_ = 1.0 on a nutrient agar (NA, Biokar) plate and incubating for 48 h at 28°C. Morphological characterization of its cellular structure was performed using Transmission Electron Microscopy (JEM 1200EX II, Jeol) at the Central Service of Electron Microscopy (SCME) from the Faculty of Veterinary of the National University of La Plata (Buenos Aires, Argentina). For this assay, SVBP6 was grown overnight on nutrient yeast broth (NYB, Biokar) at 28°C and 200 rpm.

Classical biochemical assays were performed to characterize SVBP6: Gram staining (Laboratorios Britania); catalase direct test with a drop of 3% H_2_O_2_ on a colony and evaluation of bubbling (O_2_ production); oxidase test (BD BBL™ Taxo™, N-discs with 6% of p-aminodimethylaniline monohydrochloride); gelatin liquefaction after 24 h of incubation of NYB with 12% of gelatin; glucose fermentation in Hugh and Leifson’s OF basal medium (pH = 7.4); growth on NA plates at different temperatures (4°C, 25°C, 28°C, 35°C, 37°C and 45°C); growth on Luria-Bertani (LB) broth at several pH values (from 3.0 to 13.0) and NaCl concentrations (from 0% to 11%); reduction of nitrites and nitrates after 24 h of culture growth on NYB supplemented with KNO_2_ or KNO_3_, respectively, by the evaluation of nitrite presence with n-(1-naphtyl)-ethylenediamine dihydrochloride [40] and gas formation inside a Durham tube [41]. Depending on the test performed, *Escherichia coli* K12 or *Pseudomonas aeruginosa* PA01 were employed as positive or negative controls. All incubations were done at 28°C, unless otherwise specified.

To analyze the carbon sources that SVBP6 was able to metabolize, we assayed its growth in Biolog EcoPlates^TM^ [42], after 108 h of static incubation at 30°C. Antibiotic resistance was analyzed by growing SVBP6 in NYB medium supplemented with the corresponding antibiotic at the concentrations commonly employed for the *Pseudomonas* spp. genus: chloramphenicol (20 μg/ml), ampicillin (100 μg/ml), gentamicin (10 μg/ml), kanamycin (50 μg/ml) and tetracycline (150 μg/ml).

### Evaluation of PGP properties

We tested several plant-growth promoting (PGP) activities as previously described [6]: exoprotease, phospholipase and 1-aminocyclopropane carboxylic acid (ACC) deaminase activities; hydrogen cyanide (HCN) production; siderophore and indole acetic acid (IAA) synthesis; swimming and swarming motilities; inorganic phosphate solubilization ability; biofilm development; secretion of acyl homoserine lactone-like (AHL) quorum sensing signals and the presence of antifungal-related genes (*phlD*, *phzF*, *pltB* and *prnD*). Exoprotease (milk agar plates), phospholipase (egg yolk agar plates) and siderophore (CAS agar plates) activities were relativized to the colony diameter as follows: [halo diameter (in mm)—diameter of each bacterial spot (in mm)] / diameter of each bacterial spot (in mm). Relative values were expressed as percentage.

Additionally, we tested the chitinase activity by a fluorometric assay with 4-methylumbelliferone-N-acetyl-b-D-glucosaminide as substrate [43]. Briefly, we grew SVBP6 on synthetic medium (SM) liquid medium supplemented with 10% v/v of LB broth and 0.2% w/v of colloidal chitin [44] for 72 h at 200 rpm and 28°C. Then, we collected 200 μl of supernatant by triplicate and mixed it with 50 μl of substrate at 200 μM in MES buffer 0.1M (pH = 6.1), to measure the fluorescence during 15 minutes. Control reactions and calculations of activity were done as previously described [43]. Chitinase production was expressed as the enzyme activity (nmol/min) relative to the OD_600_ value of the SM culture.

For detection of lipopeptide production, we carried out the drop-collapse test on Parafilm® [19]. The presence of biosurfactants in a drop of cell-free supernatant (from a bacterial culture grown on NYB for 16 h at 28°C and 200 rpm) decreases the surface tension of the liquid and, therefore, results in its collapse. Methylene blue was added to stain the drops for photographic purposes and had no influence on the results. Besides, a specific assay for rhamnolipids detection was performed in Siegmund Wagner (SW) agar [45], evaluating the production of halos around the colonies after plate incubation for 48 h at 28°C. Rhamnolipids form an insoluble complex with the CTAB present in the SW medium, and it interacts with the methylene blue also present. Thus, rhamnolipids producers are detected by a dark blue halo around the colony [46].

Antagonism by the production of volatile organic compounds (VOCs) was evaluated by co-cultivation assays in partitioned Petri dishes [47]. Briefly, a heavy streak of SVBP6 was performed in one side of the plate onto NA, and a 1 cm^2^ plug of a fresh *M*. *phaseolina* culture was deposited onto the other side of the plate containing potato dextrose agar (PDA). Plates were sealed with Parafilm® and incubated for 5 days at 28°C in the darkness. The assay was performed by triplicate and controls without bacteria or fungal inoculum were included.

Antibacterial activity was evaluated in two assays to analyze if it was due to a direct contact of cells (co-culture) or to the secretion of a toxin molecule (overlaid layer). For the first assay, NA plates were sown with 100 μl of a bacterial suspension of the prey with a OD_600_ = 1.0. Once plates were dried, a 10 μl drop of a SVBP6 bacterial suspension with OD_600_ = 1.0 was spotted on each one, and plates were incubated 24 h at 28°C for the optimal SVBP6 growth. For the second assay, a 10 μl drop of a similar SVBP6 bacterial suspension was spotted in the NA plates and incubated overnight at 28°C. Then, after 30 minutes of UV exposure to kill the SVBP6 cells, 4 ml of soft NA (0.8% agar) with 10% v/v of a saturated cultures of the confronted bacterial strain were overlaid above the drops and plates were incubated for 24 h at the optimal temperature of the bacterial prey. SVBP6 was confronted against *Escherichia coli* K12, a *Bacillus subtilis* strain from the bacterial collection of the Microbiology Area of the Department of Science and Technology (Universidad Nacional de Quilmes) and *Pseudomonas fluorescens* 1008 from Rizobacter Argentina S.A. NA plates with the overlaid layers of *E*. *coli* or *B*. *subtilis* strains were incubated at 37°C, or with *P*. *fluorescens* strain, at 28°C.

### Fatty acids and whole-cells protein profile analyses

Fatty acid analysis was performed by gas chromatography using the MIDI Sherlock® Microbial Identification System and the standard protocol [48]. The RTSBA6 method was employed for comparison of the FAME profile with the available library (http://www.midi-inc.com/pdf/RTSBA_6.21%20.(Environmental%20Aerobes).pdf, as available in August 2017).

Whole-cell protein profile of SVBP6 was obtained using the Ultraflex III UV-MALDI-TOF/TOF mass spectrometer and MALDI Biotyper 3.1 software (Bruker Daltonics, Bremen, Germany) at the CEQUIBIEM Institute (CONICET, Argentina) in association with the Institute of Biotechnology and Molecular Biology (IBBM, CONICET, La Plata, Argentina). The preparation of the samples was performed according to manufacturers' recommendation from a single colony cultured on LB for 24 h at 28°C [49]. Bacterial identification was performed with MALDI Biotyper Offline classification software using score values proposed by the manufacturer as follows: a score value higher than 2.0 indicates species identification; a score value between 1.7 and 1.9 indicates genus identification; and a score value lower than 1.7 indicates no taxonomic matching [50]. The *Pseudomonas* spp. database in the MALDI Biotyper software included 168 representative species (S1 Table).

### Genomic DNA preparation and sequencing

For DNA isolation, we collected 20 mg fresh weight of SVBP6 cells from an overnight culture on NA. After processing with the ZR Soil Microbe DNA MicroPrep™ kit (Zymo Research), we obtained *ca*. 9 μg of DNA with an OD_260/280_ of 1.9 and a good integrity as judged by electrophoresis in a 0.8% agarose gel. Purified DNA (approx. 300 ng) was sent to INDEAR (Rosario, Argentina) to prepare the corresponding libraries and to obtain a draft genome via the Illumina HiSeq 1500 sequencing system.

### Genome assembly and gene prediction

Genome assembly was performed with the A5 pipeline [51]. Gene prediction and genome analysis of PGP-related traits in the draft genome of SVBP6 strain were performed with the Rapid Annotation with Subsystems Technology (RAST) server database 2.0 [52], Basic Local Alignment Search Tool (BLAST®, [53]), *Pseudomonas* genome database (www.pseudomonas.com, [54]), Antibiotics & Secondary Metabolite Analysis Shell (antiSMASH) v. 3.0.5 [55] and the IslandViewer 3 software [56]. Synteny analyses were performed with the SimpleSynteny platform (https://www.dveltri.com/simplesynteny/index.html, [57]).

### Selection of genomes, identification of putative orthologous genes, alignments, and phylogenetic analysis

All draft and complete assemblies, and protein coding sequences of the genus *Pseudomonas* were downloaded via ftp from ftp.ncbi.nlm.nih.gov/genomes (April 19th, 2017). This first dataset comprised 3545 assemblies. Forty-four highly conserved ribosomal protein-coding genes were used for genetic comparison and sampling genomes (S2 Table). All 44 conserved genes were identified in 3162 genomes and these genomes were used for further analysis (S3 Table). The 44 groups of putative orthologous genes were independently aligned using Muscle [58] with the fastest algorithm (options: -maxiters 1 -diags1). Poorly aligned positions were eliminated using Gblock software with default parameters [59], and subsequently concatenated for sequence distance estimation. Genomes displaying a 99% or more similarity in these conserved genes were clustered. One genome for each cluster was randomly selected for subsequent phylogenetic analysis with preference for those with an assigned species, if available. This resulting second dataset comprise 140 selected genomes plus the one from *Pseudomonas* sp. SVBP6 reported here (Sampled Genomes in S3 Table). Sixty-seven putative orthologous genes were identified among these 141 genomes. The OrthoMCL method [60] was implemented in the Get_homologous software and used for homologous identification [61]. Blast searches were performed with a minimal identity value of 30% and minimal query coverage of 75%. Orthologous protein sequences were aligned using ClustalO v1.2.0 [62]. Again, poorly aligned positions were eliminated using Gblock with default parameters [59] and then concatenated for phylogenetic analysis. A phylogenetic tree was inferred using an approximately maximum likelihood method with an amino acid LG+G model using in FastTree 2.1 [63]. The Shimodaira-Hasegawa (SH-like) test was used to evaluate branch support. The phylogenetic position of SVBP6 was established and all genomes from the most inclusive and robust monophyletic group were selected. Genomes from this monophyletic group were used to track back all the closely related genomes to SVBP6 strain available in the original first dataset. As a result, 122 closely related genomes from the first dataset were retrieved and used for a further phylogenetic analysis (Closely related genomes in S3 Table). As previously described, putative orthologous genes among the 122 genomes plus SVBP6 strain were identified using Get_homologues package. Six hundred and seventy-six clusters of orthologous genes were found and subsequently aligned and trimmed using ClustalO v1.2.0 and Gblock, respectively. Blocks were finally concatenated, and a phylogenetic tree was built by means of FastTree 2.1, as aforementioned. The whole approach is described as a workflow in S1 Fig. A Venn diagram was constructed with R software v. 3.4.0 [64], employing the VennDiagram package and all the ORFs (PEGs) found in every *P*. *donghuensis* genome as database.

### Average nucleotide identity score (ANI)

Pairwise Two-way ANI score was computed among all closely related genomes of SVBP6 using the ani.rb script developed by Luis M. Rodriguez-R and available at enveomics.blogspot.com. ANI score is a result from a whole genome comparison. ANI index is used to delineate species from genomes sequence data [65]. Therefore, if two genomes display an ANI value of 95% or higher, both strains are believed to belong to the same species. Phylogenetic analysis was used to support ANI results.

### Tn*5* mutagenesis and screening of mutant clones with reduced antifungal activity

To obtain a clone library of random Tn*5* mutants of SVBP6, we performed a triparental conjugation with SVBP6 as the acceptor strain, *Escherichia coli* CC118 λ*pir* with the pBAMD1-4 plasmid as the donor strain and *E*. *coli* HB101 with the pRK600 plasmid as the helper strain, as previously described [66]. Briefly, from 5ml of NYB overnight cultures that were incubated at 37°C (for *E*. *coli* strains) and 35°C for SVBP6 strain (to improve the *Pseudomonas* ability to accept heterologous DNA, [67]), we combined equal volumes of the three bacterial cultures, centrifuged to obtain cellular pellets, and mixed them in a single 1.5 ml tube. We resuspended them with 50 μl of fresh NYB medium and we plated it on the border of a NA plate. The incubation was performed at 37°C for 5 h. Cells were collected with 1 ml of fresh NYB and appropriate dilutions were plated onto M9 minimal medium agar plates [49] with 0.2% of citrate as the sole carbon source, supplemented with 100 μg/ml of streptomycin [47]. We obtained approximately 2500 clones that were conserved in 384-well plates at -80°C in 20% glycerol. For selection of putative clones that had lost their antagonistic potential, we performed co-cultivation assays by streaking individual clones onto agar plates previously overlaid with a suspension of *M*. *phaseolina* 131.2010 conidia. After incubation for 48 h at 28°C, we searched for clones that did not produce a halo of fungal growth inhibition. To identify the Tn*5* insertion site in each selected clone, we carried out an arbitrary nested PCR amplification with the methodology previously described [66], followed by partial sequencing of the corresponding amplicons at Macrogen Inc. (Seoul, Korea).

### RNA extraction and purification, and Northern blot analysis

RNA preparation from SVBP6 cells was carried out essentially as described previously for *P*. *protegens* CHA0 [68], with minor modifications. Briefly, 200–500 μl of cell culture was centrifuged, and cells were resuspended in 500 μl of TKM buffer (10 mM Tris-HCl, 10 mM KCl, 5 mM MgCl_2_, pH 7.5). Washed cells were mixed with 75 μl of lysis solution (320 mM sodium acetate at pH 4.6, 8% SDS, 16 mM EDTA). Lysed cells were mixed for 5 min with 575 μl of water-saturated phenol at 65°C. After centrifugation, the supernatant was extracted once with phenol-chloroform and precipitated with 3 volumes of ethanol. The resulting RNA pellet was dissolved in diethylpyrocarbonate-treated H_2_O and kept at -80°C. RNA concentration was determined at 260 nm. Purity and integrity of RNA preparations were assessed by denaturing agarose electrophoresis and ethidium bromide staining. Northern blot analyses were performed as reported elsewhere [69]. Three micrograms of total RNA from each sample was initially electrophoresed for 45 min at a constant current (15 mA) in polyacrylamide gels (8.3 M urea, 8% [wt/vol] acrylamide, 0.2% [wt/vol] bisacrylamide in 1× Tris-borate-EDTA [TBE] buffer), with the low-range RNA ladder (Thermo Scientific, USA) serving as a molecular weight marker. The marker lane was cut and stained separately with ethidium bromide and the image registered with a UV transilluminator. The remaining gel was electroblotted at 150 mA for 30 min onto a Hybond-N membrane in 1× TBE buffer. After washing the membrane twice with 2× SSC solution (30 mM sodium citrate, 0.3 M NaCl), the RNA was cross-linked to the membrane by exposure to UV light for 5 min. The membranes were then blocked with prehybridization buffer (50% [wt/vol] formamide, 5× SSC, 50 mM phosphate buffer [pH 7.0], 2% [wt/vol] blocking reagent, 0.1% [wt/vol] N-laurylsarcosine, 7% [wt/vol] sodium dodecyl sulfate [SDS]) for 1 h at 50°C in a hybridization oven and then incubated overnight at 50°C with the hybridization buffer containing the specific anti-*rsmY* digoxigenin-labeled double-stranded DNA (dsDNA) probe (previously generated by amplification of the *rsmY* genomic locus of *P*. *protegens* CHA0) [69]. The hybridized membranes were washed under standard stringent conditions, incubated with an alkaline phosphatase-coupled anti-digoxigenin antibody solution, washed with the same buffer, and covered with the Lumiphos chemiluminescent reagent (Lumigen, USA) in the dark at room temperature for 5 min. The membranes were exposed for 60 min to photographic films and then further developed.

## Results and discussion

### Biochemical and morphological features support the assignation of SVBP6 to the *Pseudomonas* genus

**Fig 1:**
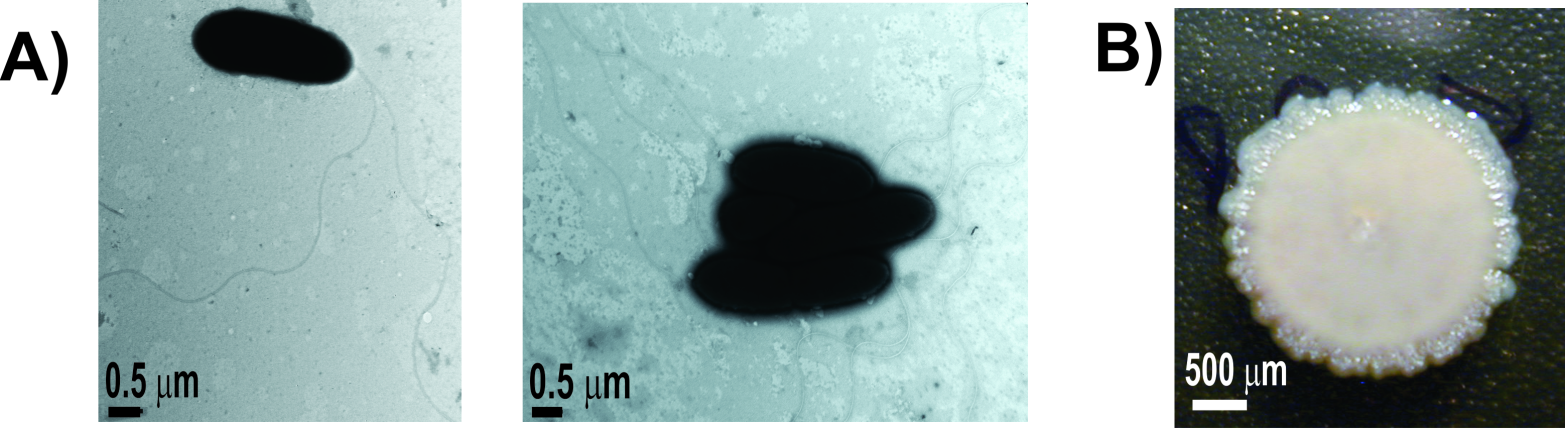
Morphological features of SVBP6 strain. SVBP6 was isolated from a bulk soil sample (0–10 cm depth) of an agricultural plot located in Viale, Entre Rios province, Argentine (31° 52’ 59,6” S; 59° 40’ 07” W). Morphological features, *i*.*e*. bacillar structure and polar flagella, of *P*. *donghuensis* SVBP6 seen with TEM **(A)**. Colony morphology on a NA plate after 48 h of growth at 28°C **(B)**.

SVBP6 is a Gram-negative, rod-shaped (between 1.3–3.2 μm in length and 0.5–0.9 μm in width) and polar-flagellated bacterium (Fig 1A) of the order *Pseudomonadales*, belonging to the *Gammaproteobacteria* class. It is oxidase, catalase and gelatinase positive, and nonsporulating. SVBP6 can grow between 4°C and 35°C in rich media, like NA, even though the optimal growth temperature is 28°C, and it belongs to the *r*-strategist (copiotrophs) group of microorganisms [8]. After 48 h on nutrient agar (NA) plates, SVBP6 colonies are white opaque, rough (non-mucoid) and have irregular borders (Fig 1B). Besides, this strain can grow in a pH range from 6.0 to 10.0 and in a salinity range from 0% to 5% NaCl in NYB medium. SVBP6 can reduce nitrate but not nitrite, so it could not participate in the soil denitrification process. Its respiration is strictly aerobic and it does not produce any fluorescent pigment, neither pyocyanin or pyoverdine, in King’s A or B or Gould’s S1 media [70]. Finally, SVBP6 strain is naturally resistant to chloramphenicol (Cm, 20 μg/ml), ampicillin (Amp, 100 μg/ml) and gentamicin (Gm, 10 μg/ml) in both liquid NYB and solid NA media. Five copies of beta-lactamase proteins, a unique copy of an aminoglycoside 6'-N-acetyltransferase, which could be responsible for the Gm resistance [71], and the efflux operon TtgABC, which give Cm resistance in *P*. *putida* KT2440 [72], were found in the SVBP6 genome. Nevertheless, these genes were not associated to mobile genetic elements and usually found in environmental isolates [73–75]. Besides, knowledge about natural antibiotics’ resistance allowed us to correctly select the mutagenesis tools for the molecular biology studies on the SVBP6 metabolism, as we confirmed we could employ plasmids marked with tetracycline and kanamycin resistance genes in the Tn5 approach.

With regards to carbon source utilization, SVBP6 was able to respire the following substrates: pyruvic acid methyl ester, Tween 40, Tween 80, glycogen, D-xylose, D-mannitol, N-acetyl-D-glucosamine, D-galactonic acid γ-lactone, 4-hydroxy benzoic acid, itaconic acid, D-malic acid, L-arginine, L-asparagine, L-serine, L-threonine, glycyl-L-glutamic acid and putrescine. Growth on L-threonine and D-mannitol can differentiate *P*. *donghuesis* species from the recently describe *P*. *wadenswilerensis* sp. nov. [24,26]. Unlike HYS strain, SVBP6 can respire itaconic acid after 108h of incubation [24]. The group of compounds that SVBP6 can respire contains representatives from all tested guilds: polymers, carbohydrates, carboxylic or acetic acids, amines and amino acids. Therefore, this strain seems to have the potential to inhabit the rhizosphere of multiple plants, as those types of compounds are generally exuded by roots from different species [76–79].

### MIDI and MALDI-TOF analyses did not allow identifying SVBP6 at the *Pseudomonas* species level

In our previous studies, phylogenetic analyses placed isolate SVBP6 within the *P*. *putida* complex. Partial sequencing of 16S rDNA, *oprF* and *rpoB* genes positioned SVBP6 close to isolates *P*. *vranovensis* T-16 and *P*. *alkylphenolica* KL28, but with similarity percentages lower than 96% [6]. Maldi-TOF Biotyper analysis gave ID score values ranging 1.75–1.84 (n = 4) with the closest match being *P*. *graminis* DSM 11363T (a member of the *P*. *lutea* group) [80]; however, such i.d. scores only correspond to identification at the level of genus. Besides, MIDI analysis did not find any match between the fatty acid profile of SVBP6 and those from the Sherlock libraries. Therefore, we compared the SVBP6 profile with those reported for *P*. *donghuensis* HYS, *P*. *putida* ATCC 12633, *P*. *vranovensis* DSM 16006 [24] and *P*. *alkylphenolica* KL28 [25], and we found the typical fatty acids associated with the *Pseudomonas* genus like C_10:0_ 3-OH, C_12:0_ and C_12:0_ 2-OH [13], as well as several unique fatty acid species for each strain (S4 Table). Particularly, SVBP6 strain contains a unique fatty acid, C13:1 Δ^12^, that was not previously described for pseudomonads, but only for *Vibrionaceae* and *Clostridium* species [81,82].

### Genome sequencing information and gene annotation

In parallel with the biochemical characterization, we decided to sequence the genome of SVBP6 strain with the aims of: 1) getting additional support for the taxonomic positioning of this isolate at the species level; 2) deciphering the molecular determinants of the strong and broad spectrum antifungal trait of this bacterium. With the protocol previously described, we sequenced 93% of the genome based on the data from the type strain *P*. *donghuensis* HYS [24]. The average GC content was 62.4% matching values described for pseudomonads (58%-69%, [13]). A total of 7,136,473 reads were assembled *de novo* into 40 scaffolds, with a mean scaffold size of 142534 bases (N_50_ scaffold length of 288994 bases). Minimum Information about the Genome Sequence [83] of strain SVBP6 is summarized in Table 1. We confirmed that SVBP6 does not contain any plasmid.

**Table 1:** Classification and minimum information about the genome sequence of *P*. *donghuensis* strain SVBP6.

MIGS ID	Property	Term	Evidence code^1^ [84]
***General features***			
	*Classification*	Domain: Bacteria	TAS
		Phylum: Proteobacteria	TAS
		Class: Gammaproteobacteria	TAS
		Order: Pseudomonadales	TAS
		Family: *Pseudomonadaceae*	TAS
		Genus: *Pseudomonas*	IDA
		Species: *Pseudomonas donghuensis*	IDA
		strain: SVBP6	TAS
	*Gram stain*	negative	IDA
	*Cell shape*	Rod	IDA
	*Motility*	Motile	IDA
	*Sporulation*	None	IDA
	*Temperature range*	Mesophilic (4–35°C)	IDA
	*Optimum temperature*	28°C	IDA
	*pH range*	6–10	IDA
	*Carbon source*	Heterotrophic	IDA
MIGS-22	*Oxygen requirement*	Aerobic	IDA
MIGS-14	*Pathogenicity*	Unknown	NAS
MIGS-6	*Habitat*	Soil	TAS
MIGS-5	*Sample collection*	February 2010	TAS
MIGS-15	*Biotic relationship*	free-living/rhizospheric	NAS
MIGS-4	*Geographic location*	Viale, Entre Ríos province	TAS
MIGS-4.1	Latitude	S 31° 52’	IDA
MIGS-4.2	Longitude	W 59° 41’	IDA
MIGS-4.3	Depth	0–10cm layer	TAS
MIGS-4.4	*Altitude*	80m above sea level	TAS
MIGS-23.1	*Isolation*	Bulk soil from an agricultural plot	TAS
***Project information***			
MIGS-31	*Finishing quality*	High-quality draft	
MIGS-31.2	*Fold coverage*	125 ×	
MIGS-28	*Libraries used*	Illumina paired-end library (Nextera® XT)	
MIGS-29	*Sequencing platforms*	Sanger / Illumina 1.9 (Illumina 1500 HiSeq)	
MIGS-30	*Assemblers*	A5 pipeline (Phred)	
MIGS-32	*Gene calling method*	RAST v. 2.0	
	*BioProject ID*	PRJNA407750	
	*GenBank accession number*	NWCB00000000	
	*GenBank Date of Release*	December 1st, 2017	
	*Project relevance*	Biocontrol, Plant growth promotion	
MIGS-13	*Source material identifier*	SVBP6	

^1^Evidence codes—**IDA** Inferred from Direct Assay, **TAS** Traceable Author Statement (i.e., a direct report exists in the literature), **NAS** Non-traceable Author Statement (i.e., not directly observed for the living, isolated sample but based on a generally accepted property for the species or anecdotal evidence). These evidence codes are from the Gene Ontology project.

RAST analysis predicted 5253 genes in total: 5179 protein encoding genes (PEGs) and 74 RNAs, of which 65 are tRNAs. 54.0% of PEGs (2757) were arranged into 527 subsystems, and a total of 1125 (21.72%) were assigned as hypothetical proteins. RAST analysis estimated a number of 90 missing genes, *i*.*e*. undercalled PEGs in the remaining gaps between features. Genome properties and statistics are summarized in Table 2. Genes with signal peptides were detected with the SignalP 4.1 Server [85], and the CRISPR repeats, with the CRISPR reconigtion tool v1.0 [86]. Details of clusters of orthologs groups are shown in S5 Table.

**Table 2:** Genome statistics.

Attribute	Value	Percentage
Genome size (bp)	5,701,342	100
DNA coding (bp)	5,005,841	87.8
DNA G+C (bp)	3,557,637	62.4
DNA scaffolds	40	-
Total genes	5253	100
Protein encoding genes (PEGs)	5179	98.6
RNA genes	74	1.4
Genes in internal clusters	118	2.2
Genes with function prediction	4054	78.3
Genes assigned to COGs	4865	92.6
Genes with Pfam domains ^a^	4450	85.9
Genes with signal peptides	512	9.9
Genes with transmembrane helices ^b^	1170	22.6
CRISPR repeats	0	0

^a^ Detected with Pfam v.31.0 (*http://pfam.xfam.org*)

^b^ Detected with TMHMM Server v. 2.0 (http://www.cbs.dtu.dk/services/TMHMM/)

### Phylogenetic identification with ANI analysis showed that strain SVBP6 belongs to the recently described species *Pseudomonas donghuensis*

**Fig 2:**
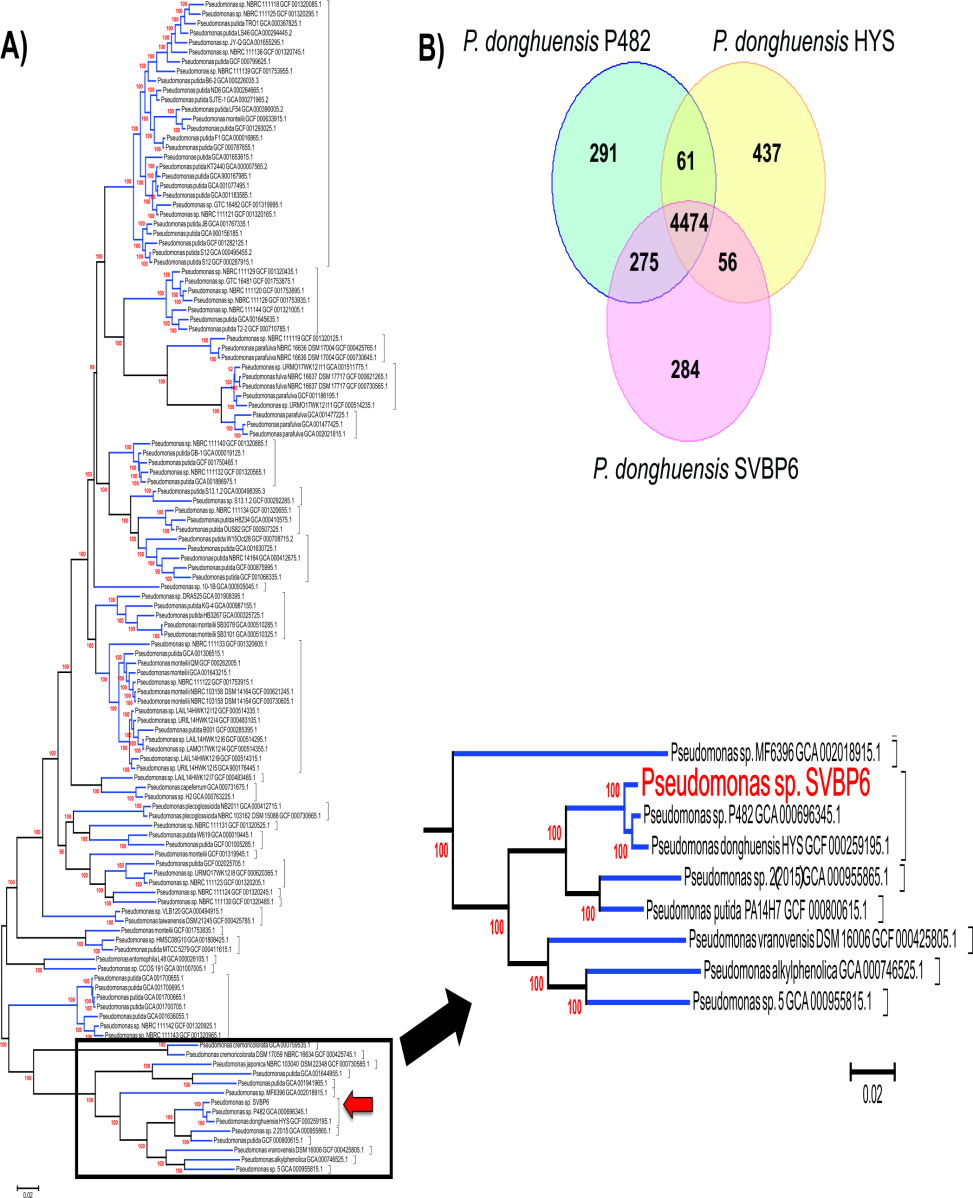
Phylogenetic identification and genome comparison. Approximate maximum likelihood phylogenetic tree of SVBP6 and closely reated *Pseudomonas* assemblies **(A)** and Venn diagram for the comparison of genomes from the three known *P*. *donghuensis* isolates, HYS (China), P482 (Poland) and SVBP6 (Argentina) **(B)**. **A)** Phylogenetic tree of the *P*. *putida* complex based on 676 putative orthologous genes. The tree was inferred using FastTree version 2.1. The SH-like test was used to evaluate branch supports. Genomes from the same species based on two-way ANI score (> 95%) were indicated with brackets. The position of SVBP6 strain is indicated by a red arrow, and this sub-cluster was zoomed to distinguish the *Pseudomonas* species around SVBP6 (in red font). Indeed, *P*. *donghuensis* strains are grouped in a sub cluster with other environmental isolates, like *P*. *alkylphenolica* KL28 [89], a biocontrol *P*. *putida* strain isolated from potato rhizosphere in France (PA14H7 strain, [90]), *P*. *vranovensis* DSM 16006 isolated from soil in the Czech Republic [91], and unidentified *Pseudomonas* sp. isolated from soil in the USA (2(2015) and 5 strains); **B)** The calculated core genome of *P*. *donghuensis* species is 4474 PEGs, between 87.7% and 89.0% of each representative. Particular PEGs of strains HYS, P482 and SVBP6 are 8.7%, 5.7% and 5.6%, respectively, and SVBP6 shares more PEGs with P482 (5.4% of total SVBP6 PEGs) than with HYS type strain (1.1% of total SVBP6 PEGs), suggesting that strains P482 and SVBP6 are more closely related than with HYS strain.

One hundred and forty genomes were selected from a first dataset as representatives of the genetic diversity of the genus *Pseudomonas* (see Methods). The phylogenetic position of SVBP6 was established among these genomes (S2 Fig). Despite minor differences, the obtained phylogenetic tree was in good agreement with previous ones reported for the genus [27]. *Pseudomonas* sp. SVBP6 was clearly positioned within the *P*. *putida* complex. One hundred and twenty-two closely related genomes of SVBP6 were selected and included in a second phylogenetic analysis. SVBP6 belongs to a basal lineage within the *P*. *putida* complex (Fig 2A), which includes at least 12 different species, according to the estimated ANI score (S6 Table). SVBP6 was closely related to *Pseudomonas* sp. 482 and *P*. *donghuensis* HYS (Fig 2A and S6 Table). The siderophore producing isolate HYS, which served to define the type species *P*. *donghuensis*, was isolated from the water of East Lake of Wuhan, China [24], whereas the bactericidal strain *Pseudomonas* sp. P482 was isolated from the rhizosphere of a garden-cultivated tomato in Gdynia, Poland [87]. ANI score supports the phylogenetic result and suggests that these three strains belong to the same species, as SVBP6 showed similarity values of 99.60% ± 0.70 and 99.52% ± 0.99 with *Pseudomonas* sp. P482 and *P*. *donghuensis* HYS, respectively [24,88]. This result was supported by a Multi-Locus Phylogenetic Analysis (MLPA) performed with concatenated 16s rDNA, *gyrB*, *rpoB* and *rpoD* sequences obtained from different genome sequencing projects of reference strains [21,27]. When we compared ORFs from the three *P*. *donghuensis* representative’s genomes by a homologous cluster analysis (minimum 50% identity, 75% coverage), we found that SVBP6 shares a higher number of ORFs with strain P482 than with strain HYS (Fig 2B). From the 284 ORFs found only in *P*. *donghuensis* SVBP6 (5.6% of the total), we could detect several mobile genetic elements (9.9%), membrane components (8.2%), putative secreted elements (3.4%), genes involved in regulation and/or metabolism (23.7%) and an important number of hypothetical proteins (54.7%).

### Genome mining: Analysis of putative PGPM traits

#### Probiotic traits tested *in vitro*

When it was isolated, the SVBP6 strain was characterized together with other 18 pseudomonads with fungal antagonistic activity. In co-cultivation experiments of phytopathogenic fungi and pseudomonad isolates, SVBP6 inhibited the growth of 12 fungal pathogens including members of the *Fusarium* and *Colletotrichum* genera, *Macrophomina phaseolina*, *Phomopsis* sp. *and Cercospora sojina* isolates [6]. The inhibitory activity against all these fungal isolates seems to be associated to a water-soluble diffusible compound that radiates in the agarized media from the SVBP6 streaks, and not to a volatile compound because SVBP6 was not able to inhibit the growth of fungi when the bacterial streaks and the fungal inocula were physically separated in the same partitioned Petri dish. Additionally, we suggest that growth inhibition potential is due to a diffusible compound because we could evaluate the antagonism of several isolates in the same Petri dish without any interference (data not shown).

From our plant-probiotic pseudomonads collection, SVBP6 was the only isolate with a high biocontrol potential index (BPI) that was not a member of the *P*. *chlororaphis* group [6]. *In vitro* assays showed that SVBP6 produces extracellular hydrolytic enzymes like proteases (relative activity: 142.4 ± 5.3 halo/colony on milk agar), chitinase (relative activity: 0.196 ± 0.034 nmol/(min*OD_600_)) and phospholipases (relative activity: 70.6 ± 4.2 halo/colony on egg yolk agar). Chitinase activity could be also referred to the ability of this strain to growth on N-acetyl-D-glucosamine (see the biochemical characterization). Besides, SVBP6 synthetizes a low quantity of hydrogen cyanide (HCN, 37.5 ± 19.6 μM/OD_600_) and a high quantity of siderophores (relative production: 145.8 ± 7.2 halo/colony on CAS agar), when compared to the reference PGPM strain *P*. *protegens* CHA0 [6]. When screening for the presence of genes and operons related to the *in vitro* antagonism of fungal pathogens (*phlD* for DAPG, *phzF* for phenazines, *pltB* for pyoluteorin and *prnD* for pyrrolnitrin [92]), we failed to detect by PCR any of the genes involved in the synthesis of broadly characterized antibiotics of biocontrol pseudomonads [6]. We could neither detect production of surfactants by the drop collapse test in different growth media, thus suggesting that strain SVBP6 does not produce lipopeptides nor rhamnolipids.

In addition to its potential as a biocontrol strain, isolate SVBP6 also displayed direct plant-probiotic activities *in vitro*, like production of IAA-like compounds, ACC deaminase, and solubilization of tricalcium phosphate [6]. SVBP6 genome did not contain the *nifH* gene encoding the nitrogenase protein necessary for this activity.

In agreement with previous *in vitro* assays [6], genome inspection did not reveal the presence of genes for biosynthesis of typical pseudomonad antibiotics, lipopeptides or quorum-sensing signals of the AHL type. However, we identified genes that are functionally linked to the PGP activities mentioned above, that were previously detected *in vitro* (Table 3).

**Table 3:** PGPR traits that were detected in vitro and *in silico* in *P*. *donghuensis* SVBP6.

Traits detected *in vitro* ^a^	Genes detected in SVBP6 genome ^b^	Sequence similarity with other *Pseudomonas* ^c^
*Exoprotease activity*	Secreted alkaline metalloprotease PrtA/B/C/G homolog (EC 3.4.24.-)	68.0% with Zn-dependent metalloprotease from *P*. *fluorescens* SBW25 (CAY49374.1)
*Phospholipase activity*	Phosphatidylcholine-hydrolyzing phospholipase C (EC 3.1.4.3)	67.5% with the phosphatidylcholine-hydrolyzing phospholipase C from *P*. *fluorescens* SBW25 (CAY47115.1)
*HCN synthesis*	Hydrogen cyanide synthases HcnA, HcnB and HcnC (Opine oxidase subunits C, A and B, respectively) in a cluster arrangement	82.0% with *hcnABC* operon from *P*. *aeruginosa* PA01 (AF208523.2)
*Chitinase activity*	Chitinase (EC 3.2.1.14)	72.7% with chitinase from *P*. *aeruginosa* PA01 (NP_250990.1)
	Chitin-binding protein	31.4% with the chitin-binding protein CbpD from *P*. *aeruginosa* PA01 (NP_249543.1)
	PTS system: N-acetylglucosamine (Nac-Glc)-specific IIA component (EC 2.7.1.69), NAcGluc-specific IIB component (EC 2.7.1.69) and NAcGlc-specific IIC component (EC 2.7.1.69)	73.4% with the N-acetyl-D-glucosamine phosphotransferase system transporter from *P*. *aeruginosa* PA01 (NP_252450.1)
	Glucosamine-6-phosphate deaminase (EC 3.5.99.6)	76.5% with a putative phosphosugar-binding protein from *P*. *fluorescens* SBW25 (CAY52003.1)
	N-acetylglucosamine-6-phosphate deacetylase (EC 3.5.1.25)	81.6% with the N-acetylglucosamine-6-phosphate deacetylase NagA from *P*. *fluorescens* SBW25 (CAY52002.1)
	Predicted transcriptional regulator of N-acetylglucosamine utilization, GntR family	80.4% with the DNA-binding transcriptional regulator (GntR family) from *P*. *aeruginosa* PA01 (NP_252446.1)
*Siderophore synthesis* ^d^^,^^e^	Putative pyoverdine cluster I (NRPS, 47.9kbp, 24 genes included)	17 genes with similarity values between 51% and 90% with the pyoverdine biosynthetic gene cluster—Locus 1 (NRPS) from *P*. *protegens* Pf-5 (CP000076.1)
	Putative pyoverdine cluster II (NRPS, 29.8kbp, 16 genes involved)	13 genes with similarity values between 46% and 89% from the pyoverdine biosynthetic gene cluster (NRPS) of *P*. *protegens* Pf-5 (NC_004129.6)
*Indole acetic acid production*	Aromatic-L-amino-acid decarboxylase (EC 4.1.1.28)	82.0% with an aromatic-L-amino-acid decarboxylase from *P*. *putida* F1 (ABQ79291.1)
*Growth with ACC as the sole nitrogen source (putative ACC deaminase synthesis)*	1-aminocyclopropane-1-carboxylate deaminase (EC 3.5.99.7)	69.0% with a putative deaminase from *P*. *fluorescens* SBW25 (CAY50289.1)
*Inorganic phosphate solubilization*	Operon for the pyrroloquinoline quinone (*pqq*) coenzyme biosynthesis (6 genes)	81.0% with the *pqq* operon (*pqqA-F*) from *P*. *putida* KT2440 (AE015451.2)
	Glucose dehydrogenase, PQQ-dependent (EC 1.1.5.2)	96.4% with the glucose dehydrogenase membrane-bound PQQ-dependent from *P*. *alkylphenolica* KL28 (AIL60333.1)
	Putative exported phosphodiesterase/ alkaline phosphatase D (PhoD)	72.2% identity with a hypothetical protein from *P*. *fluorescens* SBW25 with a metallophosphatase domain Pho-D like (CAY52149.1)
*Swimming motility*	*fli*, *flg* and *che* genes for flagellar biosynthesis and chemotaxis	84.6% with *che* operon from *P*. *fluorescens* SBW25 (AM181176.4) 84.8% with *fli* operon from *P*. *putida* KT2440 (AE015451.2) 84.1% with the *flg* cluster from *P*. *putida* KT2440 (AE015451.2)

^a^ *In vitro* results were described in Agaras et al (2015).

^b^ RAST annotation of protein functions are shown. Enzyme Commission (E.C.) numbers are described in parenthesis when they are asignated.

^c^ Values refers to gene or protein sequences, as appropriate, from a reference *Pseudomonas* strain available at the *Pseudomonas* genome database (www.pseudomonas.com). Genbank codes of those sequences are shown in parenthesis.

^d^ Clusters detected by the Antibiotics and Secondary Metabolism Analysis Shell (antiSMASH) software v. 3.0.4

^e^ Genes included in the pyoverdine gene cluster I of *P*. *donghuensis* SVBP6 showed 100% of similarity with the *P*. *putida* KT2440 genome, but without synteny.

#### Complementary putative PGP properties detected in the SVBP6 genome related with antibiotic and antifungal activities

Based on bibliography data about biocontrol-related activities demonstrated for different *Pseudomonas* members, we looked for several genes, clusters and operons in the SVBP6 genome. Although we could not find any FitD-like homologous CDS that could be involved in the production of the insecticidal protein described in other *Pseudomonas* species [93], neither the *dar* operon involved in the production of the antibiotic 2-hexyl, 5-propyl resorcinol [94], nor any of the toxin complex (Tc) clusters that were detected in several pseudomonads with insecticidal activity [95], we found a putative cluster for bacteriocin production, some elements of a type VI secretion system (T6SS), a putative toxoflavin production cluster, a monalysin-homologue gene and a *pvdQ* gene (Table 4).

**Table 4:** Genes detected *in silico* in the *P*. *donghuensis* SVBP6 genome potentially involved in plant growth promotion.

Feature	Genes detected in SVBP6 genome^a^	Sequence identity (%) with other *Pseudomonas*^b^
*Acetoin metabolism*	2,3-butanediol dehydrogenase (EC 1.1.1.4), dihydrolipoamide acetyltransferase component (E2) of acetoin dehydrogenase complex (EC 2.3.1.-), E1 component of acetoin deshydrogenase α and β subunit (EC 1.2.4.-), protein X for acetoin catabolims and transcriptional activator of *aco*operon AcoR in a cluster arrangement	85.0% with *P*. *putida* KT2440 genome sequences for the 2,3-butanediol dehydrogenase gene *bdhA* (AAN66179.1), and 82.0% with genes for an acetyltransferase component of acetoin cleaving system (AAN66180.1), an acetoin:2,6 dichlorophenolindophenol oxidoreductase β subunit (AAN66181.1)
	Acetolactate synthase small and large subunits in a cluster arrangement (EC 2.2.1.6)	88.2% with *ilvBH* genes from *P*. *chlororaphis* subsp. *aureofaciends* 30–84 WP_007924621.1 and WP_007924620.1)
*Acylase activity*	Acyl-homoserine lactone acylase PvdQ (EC 3.5.1.-)	76.1% with the *pvdQ* gene and 70.7% with the protein sequence of *Pseudomonas chlororaphis* O6 (WP_009048838.1)
*Aryl polyene synthesis*	Gene cluster for aryl-polyene biosynthesis containing 26 genes, 9 of them with known functions (EC 2.3.1.41, EC 1.1.1.100, EC 4.2.1.60, EC 4.2.1.-)	55.0% similarity with genes from resorcisol-arylpolyene cluster in *P*. *fulva* 12-X genome (YP_004472533.1, gene symbols Psefu_0453 to Psefu_0081)
*Bacteriocin synthesis*	Amidophosphoribosyltransferase (EC 2.4.2.14), colicin V production protein, DedD, bifunctional dihydrofolate synthase (EC 6.3.2.12) and folylpolyglutamate synthase (EC 6.3.2.17), acetyl-coenzyme A carboxyl transferase β chain (EC 6.4.1.2) in a cluster arrangement	89% with genes from *P*. *alkylphenolica* KL28 genome in sinteny with SVBP6 cluster (CDS from AIL60753.1 to AIL60757.1)
	tRNA pseudouridine synthase A (EC 4.2.1.70)	84.1% with tRNA pseudouridine (positions 38–40) synthase TruA from *P*. *azotoformans* (WP_078049422.1)
	DedA proteín (two copies)	87.0% with DedA phosphoesterase from *P*. *putida* AA7 (WP_079226402.1, copy 1) and 90.3% with membrane protein DedA from *P*. *mosselii* SJ10 (WP_023629651.1, copy 2)
	R-like pyocins (two holin-like proteins and three lytic enzzymes)	From 84.0% to 88.0% protein identities with R pyocins from *P*. *protegens* Pf-5 (AAY91304.1 and AAY91277.1)
	Pyocin S5-like protein and immunity protein CreA	44% protein homology with S5 pyocin from *P*. *aeruginosa* PA01 (AAG04374.1) and 100% nucleotide identity with an upstream region of the pyocin gene from *P*. *aeruginosa* PA01 (NZ_LN871187)
*Type VI secretion system (T6SS)*^*c*^	Cluster of 17 genes: a serine/threonine kinase (EC 2.7.11.1) PpkA, a phosphatase PppA, ImpM, IcmF, ImpK, ImpJ, VasD, ImpI in one group; ImpA, ImpB, ImpC, ImpD, ImpF, ImpG, ImpH, a ClpB chaperone and VgrG-like protein in another group	83.8% with nucleotide sequence of *P*. *putida* strain PC2 genome (CP011789.1, 91.6% coverage)
	TagJ1-like transmembrane protein	37% of homology with the protein sequence (93.2% of coverage) of TagJ1 from *P*. *aeruginosa* PA01 (AAG03476.1)
	Rhs-family proteins (3 putative ORFs in 4426 bp located downstream the VgrG protein from the cluster)	89.2% with the nucleotide sequence of a Rhs protein from *P*. *mosselii* SJ10 (O165_017525, 53.1% coverage)
	Rhs-family protein (4287 bp in lenght)	90.5% with a Rhs protein from *P*. *mosselii* SJ10 (WP_023630106.1, 97.7% coverage)
	VgrG-like protein	77.3% with the protein sequence of VgrG from *P*. *frederiksbergensis* (WP_086944925.1, 100% coverage)
*Toxoflavin synthesis* ^d^	Cluster of 13 genes, containing 7 genes related with toxoflavin synthesis: a membrane protein (ToxG), a RND-like transporter (ToxH), a glyoxalase (ToxM), a serine/threonine kinase (EC 2.7.11.1, ToxD), a cyclohidrolase (ToxB), a protein with unknown function (ToxC), and an O-methyltranserase (ToxA)	62.8% identity with ToxH (AAY90315.1), 47.1% with ToxG (AAY90316.1), 59.3% with ToxC (AAY90320.1), 54.6 with ToxB (AAY90321.1), 55.4% with ToxD (AAY90322.1), 50.4% with ToxA (AAY90323.1), all proteins from the toxoflavin cluster of *P*. *protegens* Pf-5. ToxM has 20.6% identity with toxoflavin degrading enzyme TflA from *Paenibacillus polymyxa* (ADK47414.1).
	Diaminohydroxyphosphoribosylaminopyrimidine deaminase (EC 3.5.4.26, ToxE)	87.5% with the RibD protein from *P*. *monteilii* CD10_2 (OAH52709.1, 100% coverage)
*Monalysin*	Hypothetical protein	34.0% of aa identity (51.4% positives, 96% coverage) with the monalysin precursor of P. entomophila L48 (WP_011534324.1)

^a^ RAST annotation of protein functions are shown. Enzyme Commission (E.C.) numbers are described in parenthesis when they are assigned.

^b^ Values refers to gene or protein sequences, as appropriate, from a reference *Pseudomonas* strain available at the *Pseudomonas* genome database (www.pseudomonas.com). Genbank codes are shown in parenthesis.

^c^ The correlation between the gene nomenclature of the T6SS cluster from SVBP6 and those from *P*. *putida* genomes was based on Cascales (2008) [112].

^d^ We included a non-pseudomonads sequence in the comparison because it is a sequence reference for the toxoflavin degrading enzymes [114].

Bacteriocins are antibiotic peptides secreted by some members of the *Enterobacteriaceae* to kill closely related bacterial cells, thereby reducing competition for essential nutrients. [96]. The SVBP6 genome revealed 8 genes that were identified by RAST as members of a bacteriocin synthesis/tolerance subsystem of the Colicin V type, which are typically arranged in a single cluster. However, in the SVBP6 genome, 5 genes related to Colicin V were clustered, and a tRNA pseudouridine synthase A gene is 2016 nucleotides upstream in the same scaffold, but two other genes encoding putative DedA proteins are located in a different scaffold (Table 4). The nucleotide sequence of the cluster is 99% identical in *P*. *donghuensis* HYS and P482 (100% coverage), including the tRNA pseudouridine synthase A and the phosphoribosylanthranilate isomerase gene (involved in tryptophan biosynthesis, EC 5.3.1.24) located in between. This group of genes establishes a synteny block not only among *P*. *donghuensis* species, but also within *P*. *alkylphenolica* (Table 4), *P*. *fluorescens*, *P*. *chlororaphis*, *P*. *brassicacearum* and *P*. *putida* representatives with more than 80% of similarity in the nucleotide sequences. It was demonstrated in *E*. *coli* that this cluster is essential for colicin production [97], but it is not involved in bacteriocin synthesis *per se*. Thus, we looked for pyocin-like genes, as this kind of bacteriocins were described in the *Pseudomonas* genus [98]. We found several genes similar to R-type pyocins, found not only in *P*. *aeruginosa* PA01, but also in *P*. *protegens* Pf-5 genome, that are considered to have evolved from phage tails to bacteriocins, performing as lytic enzymes or holins [98]. Additionally, a cluster of two CDS, one with 44% of similarity with the pyocin S5 from *P*. *aeruginosa* PA01, and an immunity protein of the CreA family, were found in the SVBP6 genome (Table 4) [99]. The CreA colicin E2 immunity protein is conserved in different *P*. *putida* genomes [100]. We also found a CDS described as a lytic enzyme, that has 57% of amino acid identity with the CvaC protein from *E*. *coli* [96]. Strains HYS and P482 were described for their antibacterial potential against the plant pathogens *Dickeya solani* and *Pectobacterium carotovorum* subsp. *brasiliense* [88], and *Xanthomonas campestris* pv. *badrii* [24], respectively. For SVBP6, we detected antibacterial activity against the Gram positive representative *Bacillus subtilis* and, to a lesser extent, against the Gram negative representative *Escherichia coli* K12 in co-cultures assays, but not to the genus-related *P*. *fluorescens* 1008 (S3 Fig).

**Fig 3:**
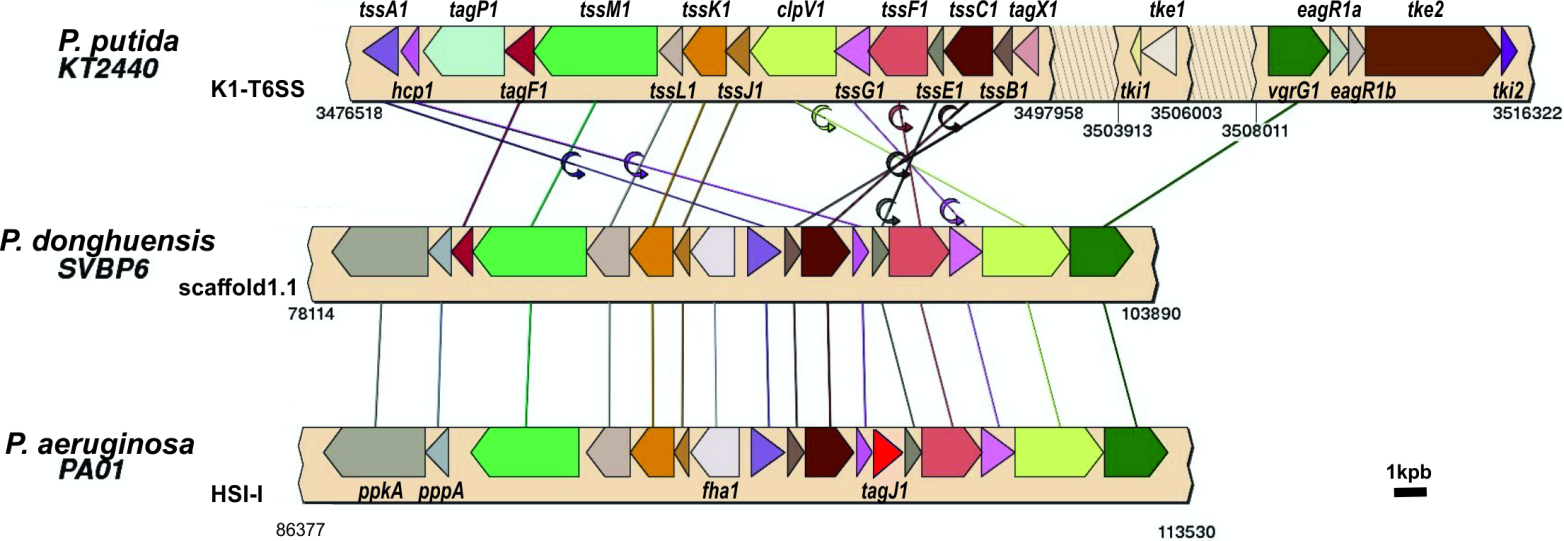
Synteny analysis between the T6SS from *P*. *putida* KT2440, *P*. *donghuensis* SVBP6 and *P*. *aeruginosa* PA01. Numbers below every cluster indicate the genome position of the cluster in each strain, although for SVBP6 the numbers indicated the position in scaffold1.1 (Genome accession numbers: NC_002516.2 for PA01 and NC_002947.4 for KT2440). Homologues in the three clusters are depicted with identical color arrows and are connected with straight lines. Changes in gene orientation are shown by a rotating arrow on the connecting lines. Gene labels of KT2440 K1 cluster are alternated above and below the corresponding arrow based on bibliography [103]. For HIS-1 of PA01, only genes not included in K1 cluster are named. Although some genes are named differently in bibliography, we unified the nomenclature, as suggested [112]. The T6SS arrangement in SVBP6 strain showed high synteny with the HSI-1 cluster from *P*. *aeruginosa* PA01, although it belongs to the *P*. *putida* group, where the *P*. *putida* is the reference strain.

The T6SS is a complex secretory apparatus with the ability to translocate effector proteins between Gram-negative bacterial cells, or to inject virulence factors into eukaryotic cells, in a contact-dependent way [101]. It has been demonstrated that this system is a powerful tool for killing competitor and phytopathogen bacteria and for shaping the bacterial community, also in soil environments [102,103]. The presence of a T6SS operon (Table 4) would confer a beneficial fitness to strain SVBP6 in the root colonization process, besides its contribution to the antibacterial activity described previously. SVBP6 contains all the structural elements of the T6SS machinery (Table 4), except for the accessory element *tagJ1*, in synteny with the T6SS cluster HSI-I of *P*. *aeruginosa* [104,105] (Fig 3). Similarly, the TagJ1 lipoprotein is also absent in several genomes from the *P*. *fluorescens* complex, indicating that it is not an essential element [106]. There is, however, one CDS with 37% of homology with TagJ1 upstream the the SVBP6 bacteriocin cluster, which was also identified as a transmembrane protein by RAST (Table 4). Indeed, the SVBP6 genome contains the six T6SS proteins–TssA, TssE, TssF, TssG, TssK and VgrG–that are required for the proper assembly of the T6SS tail tube and system functioning [107]. On the other hand, there is no synteny between the HSI-I-like cluster of SVBP6 and the K1-T6SS cluster found in the reference strain of the *P*. *putida* complex, KT2440 [103], although they contain the same genes (Fig 3). Besides, there is an additional VgrG-like protein in the SVBP6 genome, as it occurs in *P*. *aeruginosa* [104]. Although it appears that SVBP6 could construct the complete T6SS structure, homologous proteins to the effectors Tse and Tke described in *P*. *aeruginosa* PA01 and *P*. *putida* KT2440, respectively, were not found in the SVBP6 genome [101,103]. Downstream the *vgrG* gene of the HSI-I cluster, there are 3 ORFs recognized as Rhs-family proteins by RAST and the first two *rhs*-like genes had been exclusively detected in the SVBP6 genome compared with the HYS and P482 *P*. *donghuensis* isolates (Fig 2B). This kind of protein was previously described as putative effectors in the T6SS of different bacteria [108]. Also, we found a copy of a Rhs-family protein of 1428 aa (Table 4) that contains a typical PAAR (proline-alanine-alanine-arginine) domain in the N terminal region, which could help effectors to be attached to the VgrG spike [102]. A recent report describes the relevant role of the *rhsA* gene from *P*. *protegens* Pf-5 on its competitiveness against a target *P*. *putida* KT2440 strain, as encoding a polypeptide with putative DNAse activity, [109]. Besides the RhsA domain and the Rhs repeat-associated core, we detected 3 transmembrane helices in the N-terminal region of this Rhs-family protein with the Philius transmembrane prediction tool (http://www.yeastrc.org/philius/runPhilius.do) and it was also detected with the TMHMM tool as one of the SVBP6 genome features with a transmembrane portion (Table 2). A similar transmembrane region was described for the TccC insecticidal toxin protein of *P*. *taiwanensis* BCRC 17751 [110]. Thus, although we did not find a putative Tc cluster, SVBP6 contains a CDS that could perform as an insecticidal protein like the TccC toxin, which has been demonstrated to be sufficient for an insecticidal activity [110,111]. Downstream the *rhsA*-like gene, from position 476325 to 476603 of the scaffold 2.1, we found a putative CDS that was not annotated by RAST. We detected this region by its similarity (95.3% sequence identity, 100% coverage) with a gene annotated as a hypothetical protein (CDS WP023630107.1), which is also located downstream the effector protein of *P*. *mosselii* SJ10 to which the RhsA-like protein was similar (Table 4). Usually, immunity proteins against T6SS effectors are located immediately downstream the toxin gene [103,104,109]. Thus, this still unknown protein could have this putative function for SVBP6. Further experimental work is required to prove the functionality of the T6SS genes found in the SVBP6 genome, to identify the putative delivered proteins in this strain and to confirm the function of these Rhs family proteins.

**Fig 4:**
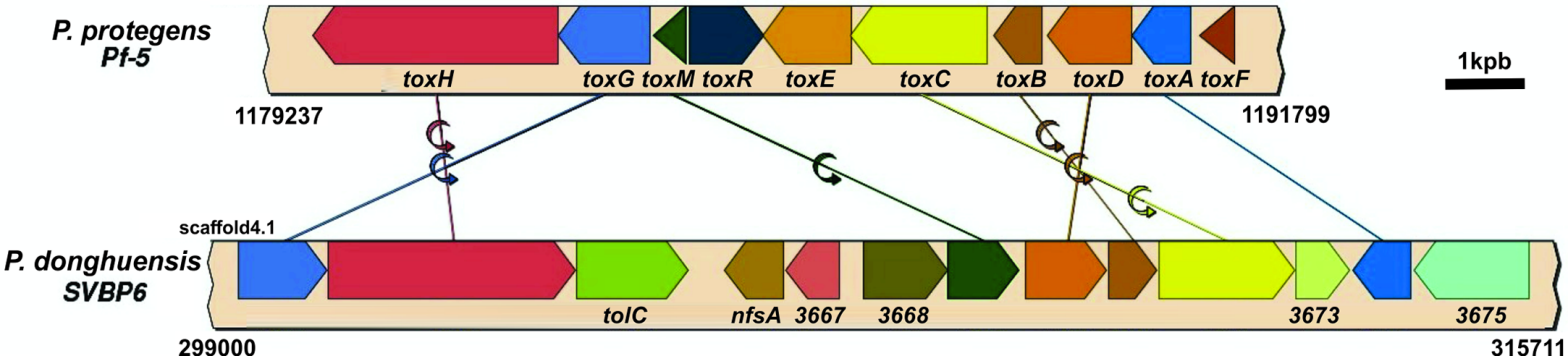
Synteny analysis between the toxoflavin cluster from *P*. *protegens* Pf-5 and *P*. *donghuensis* SVBP6. Numbers below every cluster indicate the genome position of the cluster in each strain, although for SVBP6 the numbers indicated the position in scaffold4.1. Homologues in the two clusters are depicted with identical color arrows and are connected with straight lines. Changes in gene orientation are shown by a rotating arrow on the connecting lines. Gene labels of Pf-5 toxoflavin cluster are below the corresponding arrow. Additional genes were named below the SVBP6 cluster. Although most of the Tox genes are present in the SVBP6 genome, the arrangement is different from previously described in the Pf-5 genome (Acession number NC_004129.6).

Toxoflavin has been demonstrated to be an effective antibiotic compound against bacteria and fungi. First discovered in *Burkholderia gladioli* and described as a phytotoxic compound [113], toxoflavin produced by the probiotic strain *P*. *protegens* Pf-5 was reported to inhibit the growth of several plant-pathogenic bacteria in microaerobic conditions [114]. In the SVBP6 genome, there is a cluster of 13 genes that contains 7 genes homologous with those from the *P*. *protegens* Pf-5 cluster (Table 4). Interestingly, there is no synteny between both clusters (Fig 4); moreover, we found a group of 4 genes between the ToxH-ToxG homologous transport proteins and the ToxA-ToxD biosynthetic proteins that seems not to be related with the toxoflavin biosynthesis (Fig 4). ToxM is a protein essential for toxoflavin resistance and a putative antivirulence compound against some phytopathogenic bacteria due to its toxoflavin-degrading activity [114,115]. The SVBP6 homologue of this protein has low identity with the corresponding Pf-5 amino acid (aa) sequence (14.5% identity with gaps) and a disparity in their size (137 aa for ToxM and 296 aa for the SVBP6 version). However, it has a moderate similarity with the well-known toxoflavin lyase TflA from *Paenibacillus polymyxa* JH2, which has 222 aa (20.6% of identity, 38.2% of positives) [115]. On the other hand, feature 3667 is a transcriptional regulator of the ArsR family, and it could act as the lacking LysR-like regulator ToxR; TolC is a transport protein that could help with the toxoflavin efflux; 3668 and 3673 features seem to have a methyltransferase activity, like the ToxA protein; and the 3675 feature has a moderate aa homology (24% identities, 44% positives) with the ToxI protein from *Burkholderia gladioli*, which acts as a multidrug transporter [113]. An important aspect is the lack of the ToxE protein in the SVBP6 cluster, encoding a RibD synthetase of the riboflavin biosynthesis that is essential for the toxoflavin production [114]. We found in the SVBP6 genome a CDS with 42% of aa identity and 59% of aa similarity, with the ToxE protein of *P*. *protegens* Pf-5, located in the riboflavin biosynthetic cluster (Table 4). Although it could be involved in the toxoflavin synthesis, this particular arrangement of the Tox homologous genes in the SVBP6 genome does not allow us to predict any putative toxoflavin production without any functional assay. However, the antibacterial activity we detected against the Enterobacteria *E*. *coli* K12 could be attributed to toxoflavin, as it has been demonstrated for *P*. *protegens* Pf-5 against *E*. *coli* DH5α [114].

Monalysin is a toxin described in the entomopathogenic strain *P*. *entomophila* L48 as one of the main virulence factors against *Drosophila*. This protein causes intestinal cell damage in *Drosophila melanogaster* via the formation of β-barrel membrane pores [116]. In the SVBP6 genome, we found a sequence with moderate homology with a precursor of this toxin (Table 4). Opota and collegues have demonstrated that this protein requires a N-terminal cleavage to become fully active, and this post-translational modification of monalysin is due to the AprA metalloprotease [116]. Indeed, we have described a exoprotease activity *in vitro* of SVBP6 isolate, and we have found a gene codifying an alkaline protease homologue to AprA in its genome (Table 3), thus a mature and active monalysin could be produced by SVBP6. Therefore, this strain could act also as a pest control agent [117].

The *pvdQ* gene encodes an acylase that is probably involved in a mechanism for acyl group removal before release of pyoverdine outside the cell [118]. This gene was located separately of the two putative pyoverdine clusters found In the SVBP6 genome (Table 3). Interestingly, PvdQ also hydrolyzes the amide bond connecting the acyl group to the lactone ring in several long-chain N-acyl homoserine lactones [119]. Although PvdQ might have a pyoverdine intermediate as the natural substrate, its periplasmic location has led to the suggestion that it might also behave as a quorum quencher (QQ), which has been demonstrated to be a biocontrol mechanism against bacterial pathogens [120].

#### Additional traits present in its genome that could help SVBP6 performing better as an agricultural bio-input

In addition to biocontrol traits, genome mining allowed us to find other putative PGPM genetic determinants in the SVBP6 draft genome: genes related with acetoin metabolism, a putative aryl-polyene biosynthesis cluster, and a recombinase involved in colonization (Table 4).

Acetoin-related genes present in the SVBP6 genome are probably involved in synthesis and utilization of this organic compound [121]. As in other probiotic pseudomonads, this metabolic pathway seems not to include genes described in the *bud* operon of *Bacillus* spp. and *Enterobacteriaceae* [106]. Instead, SVBP6 showed a putative *aco* operon, as it was described in *P*. *putida* PpG2 [122], and the *ilvBN* genes for the acetoin synthesis (Table 4). This alternative pathway for the acetoin and 2,3-butanediol metabolism was also found in several probiotic pseudomonads from the *P*. *fluorescens* complex [106]. The *aco* operon, that was identified also with AntiSMASH as a putative cluster, includes 6 genes identified by RAST as the three subunits of an acetoin deshydrogenase, the regulatory component AcoR, an AcoX protein of unknown function, and an acetoin reductase, also known as butanediol deshydrogenase *bdh*, that could be involved in 2,3-butanediol synthesis, as it occurs in *P*. *chlororaphis* O6 [106]. *ilvBN* genes encode an α-acetohydroxyacid synthase that could produce the acetoin via the spontaneous decomposition of α-acetolactate in acetoin and diacetyl. These reactions would supply the *budAB* operon described in *Bacillus* and *Enterobacteriaceae*. Acetoin and 2,3-butanediol can trigger the induced systemic resistance (ISR) system in plants and also promote its growth [123–126]. Besides, both compounds, but specially 2,3-butanediol, have also several industrial applications and their microbial production, with a biotechnological improvement, is increasingly interesting [127].

Aryl-polyenes (APEs) are lipids with an aryl head group conjugated to a polyene tail, that are produced by all subphyla of Proteobacteria and some genera from the *Cytophaga*-*Flavobacterium*-*Bacteroides* group [128]. These compounds have the ability to protect cells against oxidative stress, scavenging reactive oxygen species (ROS) such as peroxy radicals or singlet oxygen due to their conjugated double bond systems [129]. In the cluster found in SVBP6 genome, 40% of genes show similarity with the APE cluster from *Vibrio fischeri* ES114 chromosome I, including some putative 3-oxoacyl-[ACP]-synthase and reductase, acyl/glycosyltransferase and ammonia lyase genes. Therefore, SVBP6 strain might produce an APE with a biochemical structure more similar to APE_Vf_ (subfamily 2) than to xanthomonadins, arcuflavins or flexirubins [128,129].

Another beneficial trait of strain SVBP6 related with the colonization competence is the presence of a genomic island (GI, 64.2% GC, detected by the IslandPath-DIMOB prediction method of IslandViewer) that contains a recombinase gene in a specific genetic arrangement that has been previously described to be essential in the competition process of root colonization of tomato and potato plants [130], and to improve the colonization competence and biocontrol activity against *Fusarium oxysporum* f. sp. *radicis-lycopersici* in tomato of other two *P*. *fluorescens* strains, F113 and WCS307 [131]. Root colonization is often a limiting step in biocontrol processes, thus the presence of this gene would provide SVBP6 with a putative advantage at the colonization level and a better control of disease development, beyond the antagonistic mechanism carried out specifically against every pathogen. Although we found genomic islands in the SVBP6 genome, we did not find any gene from a type IV secretion system (T4SS), generally involved in the horizontal gene transfer [132] and recently described in *P*. *putida* W15Oct28 [100]. This result correlates with the low twitching motility observed for SVBP6 *in vitro* [6], a movement mediated by the type IV pili [133].

The genome exploration with the antiSMASH tool also revealed the presence of a cluster of mangotoxin-related genes. Mangotoxin is the molecular determinant of the bacterial apical necrosis caused by different *P*. *syringae* strains [134]. Nevertheless, we only identified an *mgo* operon in the SVBP6 genome, which has a regulatory function [134], but we could not detect homologs of the *mboB* and *mboC* genes that are directly involved in the synthesis of this toxin [135]. Besides, this operon was described to produce a signal molecule involved in *P*. *entomophila* pathogenesis in *Drosophila melanogaster* via the non-ribosomal peptide synthetase (NRPS) PvfC, an homologous protein of MgoA [116,136]. The lack of key virulence genes present in diverse pathogenic *Pseudomonas* species [137] and the implication of this operon in a regulatory system, supports the development of SVBP6 strain as a safe biocontrol agent for its application to seeds or plants. Moreover, the analysis of GI with IslandViewer 3 confirmed the absence of virulence factors, resistance genes and pathogen-associated genes in this kind of arrangement.

An additional mechanism involved in cell survival is present in the SVBP6 genome. We found a cluster containing genes for the synthesis, regulation and degradation of polyhydroxyalkanoates (PHAs), and two phasin genes (*phaI* and *phaF*), with a syntenic organization with respect to those described previously in the *Pseudomonas* genus [138]. Under certain starvation circumstances, like those found in the soil environment, bacterial cells with a higher content of PHAs may survive better than those with a lower PHA content because they can utilize their storage material for longer periods and more efficiently. Besides, soil conditions (particularly in the rhizosphere) are optimal for bacterial PHA production because a high C:N ratio prevails [139]. Therefore, the genetic potential to synthetize and degrade PHAs would allow SVBP6 to better persist in the rhizosphere, and improve its survival also in inoculant formulations until the product is applied by the farmer [140].

All the set of those putative PGPM-related traits present in the SVBP6 genome previously described *in vitro* and/or *in silico*, suggest that this strain is well prepared to survive in a complex and competitive environment such the plant rhizosphere, where a continuous war between microbes happens [141].

Besides all these activities described above, *P*. *donghuensis* SVBP6 possesses several metabolic pathways for the catabolism of organic compounds, being some of them known industrial wastes and environmental pollutants. Genes included in the widely distributed phenylacetyl-CoA catabolon are encoded in the SVBP6 genome. The entire PhAc catabolic pathway is located within a 16 kb fragment and is composed of 16 genes with an arrangement similar to the cluster found in *P*. *putida* U [142]. The degradation of those polymers, which have plastic properties, is carried out when the carbon source has been exhausted from the media, thus providing this bacterium with advantages for its survival [143]. Also, some putative substrates of this pathway, like styrene, are toxic compounds [142]. Benzoate is the simplest aromatic salt and it is an intermediate of the biodegradation of many aromatic compounds, such as toluene. The SVBP6 genome contains genes involved in benzoate and catechol degradation, possibly via the *ortho* pathway because of the presence of a muconolactone putative gene [144]. Thus, the ability to mineralize some complex organic compounds is an additional interesting feature of strain SVBP6 for exploration of possible bioremediation/biodegradation purposes.

The whole set of putative PGP traits found in the SVBP6 genome by means of the genome mining we performed represents a start point to investigate if these putative activities are in fact relevant for the strain’s performance *in vitro* and, particularly, *in planta*, an also for discovering new probiotic mechanisms [28].

### The GacS-GacA two component system controls expression of biocontrol related traits in strain SVBP6

**Fig 5:**
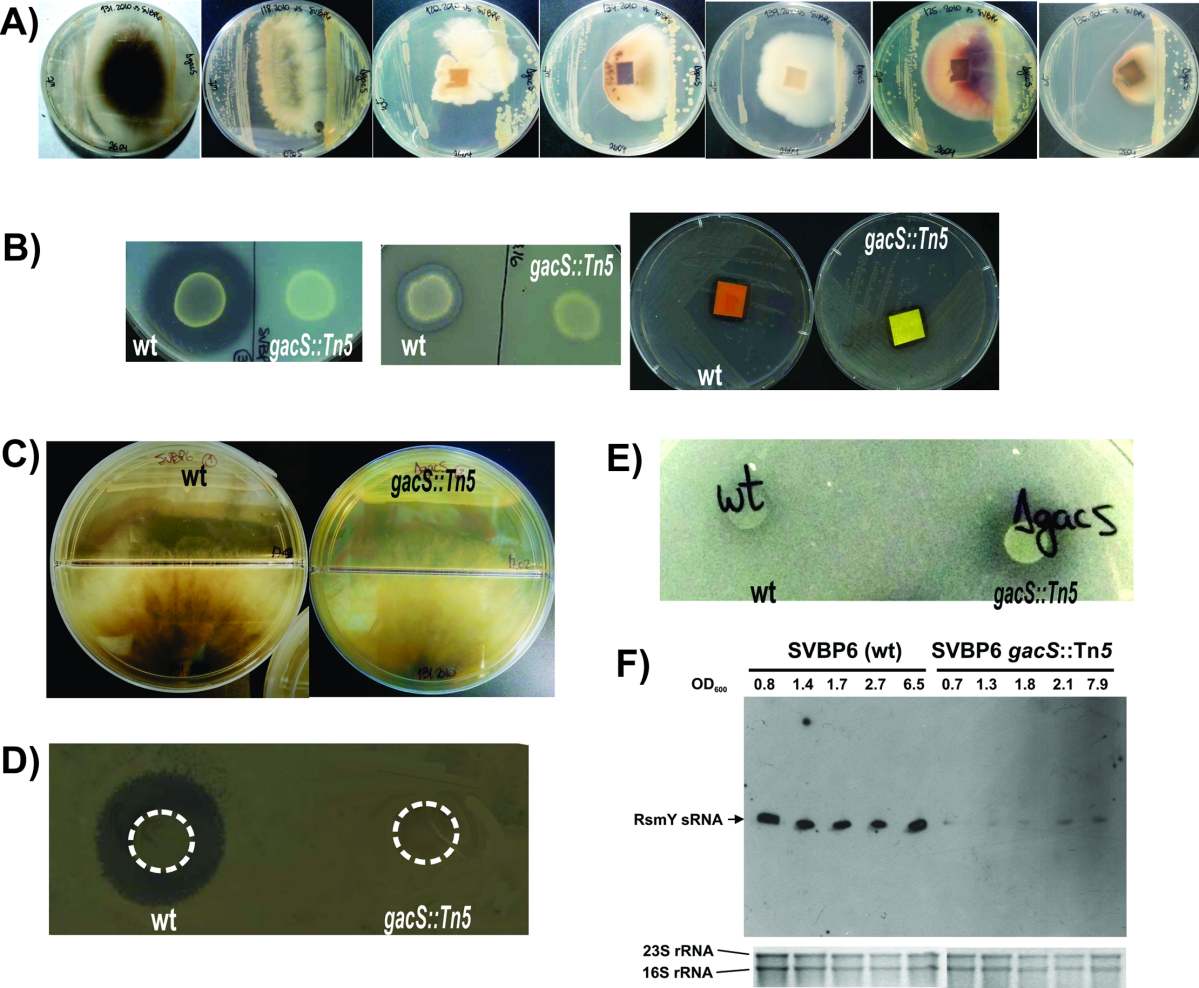
Phenotypic *in vitro* assays of the *gacS*::Tn*5* mutant compared with the wild type SVBP6 strain. **A)** Dual plate assays on PDA medium of wild-type SVBP6 (left strike) and the *gacS*::Tn*5* clone (right strike) against 7 fungal pathogens (from left to right): *Macrophomina phaseolina*, *Colletotrichum truncatum*, *Fusarium semitectum*, *Phomopsis sp*., *Fusarium solani*, *Fusarium oxysporum* and *Colletotrichum graminicola*, being all isolates reported elsewhere [6]. In all cases, the *gacS*::Tn*5* mutant showed a marked reduction in its antagonistic potential against those phytopathogens; **B)** Exoprotease, phospholipase, HCN production The *gacS*::Tn*5* mutant lost its activity, in agreement with the reported activation by the GacS/GacA system in other plant-probiotic pseudomonads; **C)** Antagonism evaluated in partitioned Petri dishes to evaluate the inhibition by VOCs. In this experiment, VOCs production seemed not to be involved in the SVBP6 antagonism against *M*. *phaseolina* 131.2010; **D)** Antibacterial activity against *B*. *subtilis* in an overlaid assay. The *gacS*::Tn*5* mutant lost its activity in both (shown in picture), overlaid and co-culture assays (data not shown); colony edge is marked with a white dotted line; **E)** Inorganic phosphate solubilization assay performed in NBRIP medium. The *gacS*::Tn*5* mutant showed a bigger solubilization halo than the wild type strain; **F)** The transcription of the sRNA RsmY was analyzed by Northern blot, as previously described [68]. While rRNA 23S and 16S levels kept constant between wild type strain and *gacS*::Tn5 mutant, RsmY abundance is markedly reduced in *gacS*::Tn*5* mutant, even in RNA samples from a saturated culture with a high OD_600_ value.

To elucidate the mechanisms involved in the antagonistic potential of SVBP6, we decided to construct a collection of Tn*5* mutants and to screen for those clones that had lost their antagonistic potential against the fungal phytopathogenic isolate *M*. *phaseolina* 131.2010 [6]. From more than 2500 Tn*5* mutants, we selected a set of 50 clones that lost their antifungal potential against the indicator fungal strain 131.2010 and therefore, they are under characterization. Here, we report the features of one SVBP6 Tn*5* mutant clone that lost its antagonistic potential not only against 131.2010, but also against all the other 11 fungal phytopathogens that were inhibited by the SVBP6 wild type strain (Fig 5A). The Tn*5* insertion site was determined by arbitrary PCR [66] and the disrupted gene was found to be a *gacS* homologue. Like in other plant probiotic *Pseudomonas* strains [145], it seems like the GacS-GacA two component system would be regulating the expression of genes involved in the antagonistic potential of SVBP6 strain. Moreover, we confirmed that this *gacS*::Tn*5* mutant could not produce HCN, neither it showed exoprotease nor phospholipase activities (Fig 5B), all of which have been described to be controlled by this regulatory system in other plant-probiotic pseudomonads [146,147]. Contrary to what has been previously observed for other biocontrol pseudomonads [148] and for the close relative *P*. *donghuensis* P482 strain, in which the GacS-GacA system positively controls the production of several VOCs involved in antagonism against fungi and oomycetes [149], we did not detect any VOCs involved in the antagonistic potential of SVBP6 against *M*. *phaseolina* growth, as SVBP6 was not able to inhibit the growth of fungi when the bacterial streaks and the fungal inocula were physically separated in the same partitioned Petri dish (Fig 5C). However, the antibacterial activity of SVBP6 resulted to be strongly dependent on the presence of a functional GacS-GacA system, because the *gacS*::Tn*5* mutant could no longer inhibit the growth of any tested bacterial targets (Fig 5D). Accordingly, a *P*. *protegens* Pf-5 with an in-frame knockout of *gacA* produced negligible amounts of toxoflavin [114], although the toxicity of this compound was not demonstrated against Gram positive bacteria. Besides the impact on biocontrol-related activities, we detected that the SVBP6 *gacS*::Tn*5* mutant was able to better solubilize inorganic phosphate in NBRIP medium (Fig 5E), in agreement with the phenotype reported for a *P*. *fluorescens* SBW25 Δ*gacS* mutant that showed deregulation of the PQQ operon [150].

Bioinformatic analysis of the SVBP6 genome allowed us to detect the presence of all genetic elements of a typical Gac/Rsm global regulatory cascade [151]: a *gacS* gene encoding the membrane associated histidine kinase sensor, which was one of the targets of *Tn5* in our mutagenesis approach (Fig 5); the *gacA* gene encoding the GacS cognate transcriptional activator of expression of the dedicated regulatory sRNA genes; three homologue genes encoding members of the CsrA/RsmA family of RNA-binding and translational regulators; and one copy of each of the *rsmZ* and *rsmY* sRNA gene homologues whose transcripts are regulatory sponges that titrate CsrA/RsmA proteins and therefore activate translation of different mRNAs [151]. The sequence similarity of the SVBP6 *rsmY* and *rsmZ* homologues were found to be 91% and 93% with those from *P*. *protegens* CHA0, respectively [67,151]. Such degree of identity allowed us to confirm expression of the SVBP6 *rsmY* homologue in the wild type strain by Northern blot using the *P*. *protegens* CHA0 dsDNA probe. The SVBP6 RsmY was strongly expressed all along the growth curve, but its cellular abundance was markedly downregulated in the *gacS*::Tn*5* mutant clone (Fig 5E). Further directed mutagenesis is required to verify expression and roles of all identified members of the Gac/Rsm cascade in the control of SVBP6 antagonistic traits.

## Conclusions

As classical phylogenetic assays did not allow us to assign SVBP6 to any of the known *Pseudomonas* species, we carried out its genome sequencing and biochemical characterization. The assembled draft genome of SVBP6 consisted of 5,701,342 bases, for which 5253 PEGS were assigned, being 78.3% of those coding sequences related to a known subsystem (Table 2). The phylogenetic comparison based on ANI between the strain SVBP6 and other sequenced *Pseudomonas* spp. strains, revealed the closest relationship with strains HYS and P482 of the recently described *P*. *donghuensis* species (S6 Table). These three *P*. *donghuensis* isolates formed a discrete clade within a larger group conformed by numerous *P*. *putida* strains (Fig 2; S2 Fig). Genome prospecting allowed detection of genes and operons supporting previous experimental data reporting different PGPR traits, such as siderophore production, exoprotease, phospholipase and ACC deaminase activities, and biosynthesis of IAA-like compounds (Table 3). Nonetheless, genome mining revealed several additional potential plant-probiotic and root colonization traits that would be powerful tools for SVBP6 strain to act as a biocontrol agent (Table 4), including T6SS, bacteriocin, toxoflavin, monalysin, and aryl polyene syntheses, acetoin metabolism and acylase activity. Besides, we detected several genetic determinants for metabolic pathways related to degradation of organic compounds that could be interesting for bioremediation purposes. Finally, we confirmed that, regardless the chemical nature of the broad antifungal activity, the GacS sensor and most likely all the downstream elements of the identified Gac/Rsm cascade, has a prominent role in activating the biosynthesis of the diffusible inhibitory molecule(s) (Fig 5). Thus, both the genome mining and the Tn*5* mutant library, will allow us to deeply investigate the potential of SVBP6, and hence to found new mechanisms that could explain its broad antifungal activity.

This is the first report of a *P*. *donghuensis* isolate obtained from a bulk soil sample, and from the Southern hemisphere. Similarly to what has been recently described for the Polish tomato-rhizospheric isolate *P*. *donghuensis* P482, SVBP6 showed a strong antagonistic activity against a variety of fungal pathogens, but, in contrast to isolate P482, it appears that the antagonistic mechanism is not based on the production of VOCs. Besides, SVBP6 displayed antibacterial activity against a Gram-positive representative (*B*. *subtilis*) and an Enterobacterial representative (*E*. *coli*), a feature that could be attributed to the expression of identified bacteriocin or toxoflavin production genes. The availability of the genome sequence of this isolate, as well as the successful generation of a random Tn*5* mutagenic clone library, will provide the basis for the elucidation of the genetic basis responsible for the broad antifungal activity of *P*. *donghuensis* SVBP6 isolate.

## Supporting information

S1 Table *Pseudomonas* species included in the MALDI Biotyper database. List of bacterial strains of the *Pseudomonas* genus employed by the MALDI software to compare and identify a sample.
(PDF)

S2 Table List of conserved genes used for sampling genomes based on genetic diversity. Genes in the reference genome of *Pseudomonas aeruginosa* PAO1 (Assembly Acc. GCA_000006765.1) are indicated.
(PDF)

S3 Table Pseudomonas strains' sequences analyzed in this study. (PDF)

S4 Table Fatty acids patterns of *P*. *donghuensis* SVBP6 and some related pseudomonads. The MIDI profile of SVBP6 was compared with bibliography data of closely related *Pseudomonas* species.
(PDF)

S5 Table Number of genes associated with general COG functional categories in the SVBP6 genome. Classification of the COGs by functional categories with one-letter abbreviations for the functional categories was based in the COG database [152].
(PDF)

S6 Table ANI scores. Detailed values of the ANI analysis of the 142 more closely related *Pseudomonas* genomes that were chosen to perform the analysis (See materials and methods for further explanation). ANI score is a result from a whole genome comparison. Values higher than 97% of similarity among strains are shown in red. *P*. *donghuensis* HYS and *Pseudomonas* sp. P482 showed high similarity values with SVBP6 strain.
(PDF)

S1 Fig Workflow of the phylogenetic analyses done in the present study. (PDF)

S2 Fig Phylogenetic tree of *Pseudomonas* based on 67 putative orthologous genes. Tree was inferred using an approximate maximum-likelihood method with default parameters by means of FastTree version 2.1. The SH test was used to evaluate branch supports. The 140 genomes other than SVBP6 that were included in this analysis were selected as representatives of the genetic diversity of the genus (see text). Red arrow indicates the position of SVBP6 strain. The monophyletic group selected for further phylogenetic analysis is indicated within a blue box.
(TIF)

S3 Fig Antibacterial activity of *P*. *donghuensis* SVBP6 against a Gram-positive prey (*B*. *subtilis*) and two Gram-negative preys, one from the same genus (*P*. *fluorescens* 1008) and from the Enterobacteriacea group (*E*. *coli K12*). **A)** Overlaid layer assay, showing a putative release metabolite that could exert the antibacterial activity; **B)** Co-culture assay, showing a putative secreted metabolite or even a contact-dependent strategy, as T6SS.
(TIF)
