# Supplementary material for: Genomic insights into the broad antifungal activity, plant-probiotic properties, and their regulation, in Pseudomonas donghuensis strain SVBP6
Source: PLoS One. 2018 Mar 14;13(3):e0194088. doi: 10.1371/journal.pone.0194088 (PMC5851621; doi:10.1371/journal.pone.0194088)
Supplement: S1 Table — List of bacterial strains of the Pseudomonas genus employed by the MALDI software to compare and identify a sample. (PDF) [file pone.0194088.s001.pdf]

MSP Name	Creation Date
Pseudomonas abietaniphila CIP 106708T HAM	17/01/2007 16:27
Pseudomonas aeruginosa 19955 1 CHB	24/01/2007 15:07
Pseudomonas aeruginosa 8147 2 CHB	17/01/2007 16:28
Pseudomonas aeruginosa A07 08 Pudu FLR	30/03/2009 13:26
Pseudomonas aeruginosa ATCC 27853 THL	17/01/2007 16:28
Pseudomonas aeruginosa DSM 1117 DSM	28/04/2011 15:17
Pseudomonas aeruginosa DSM 1128 DSM	06/03/2013 16:52
Pseudomonas aeruginosa DSM 50071T HAM	17/01/2007 16:29
Pseudomonas aeruginosa DSM 50071T QC D...	22/01/2013 8:43
Pseudomonas aeruginosa LMG 8029 LMG	21/09/2012 11:59
Pseudomonas agarici DSM 11810T HAM	17/01/2007 16:29
Pseudomonas alcaligenes 4 3 A TUB	16/02/2010 13:07
Pseudomonas alcaligenes DSM 50342T HAM	17/01/2007 16:30
Pseudomonas alcaliphila DSM 17744T LGL	02/02/2012 8:59
Pseudomonas anguilliseptica DSM 12111T HAM	17/01/2007 16:31
Pseudomonas antarctica DSM 15318T HAM	17/01/2007 16:32
Pseudomonas asplenii LMG 2137T HAM	17/01/2007 16:32
Pseudomonas avellanae DSM 11809T DSM	02/09/2009 9:04
Pseudomonas azotifigens DSM 17556T LGL	01/02/2012 10:17
Pseudomonas azotoformans CIP 106744T HAM	17/01/2007 16:34
Pseudomonas balearica B397 UFL	30/01/2007 18:07
Pseudomonas balearica DSM 6083T HAM	17/01/2007 16:35
Pseudomonas boreopolis LMG 979T HAM	17/01/2007 16:36
Pseudomonas brassicacearum DSM 13227T H...	17/01/2007 16:37
Pseudomonas brenneri CIP 106646T HAM	17/01/2007 16:38
Pseudomonas caricapapayae LMG 2152T HAM	17/01/2007 16:38
Pseudomonas cedrina ssp cedrina CIP 105541...	17/01/2007 16:39
Pseudomonas chlororaphis ssp aurantiaca CIP ...	17/01/2007 16:33
Pseudomonas chlororaphis ssp chlororaphis D...	17/01/2007 16:40
Pseudomonas cichorii DSM 50259T HAM	17/01/2007 16:41
Pseudomonas citronellolis 993700254 LBK	02/02/2010 14:35
Pseudomonas citronellolis DSM 50332T HAM	17/01/2007 16:42
Pseudomonas congelans DSM 14939T HAM	17/01/2007 16:43
Pseudomonas corrugata DSM 7228T HAM	17/01/2007 16:43
Pseudomonas extremorientalis DSM 15824T H...	17/01/2007 16:44
Pseudomonas flavescens DSM 12071T HAM	17/01/2007 16:45
Pseudomonas fluorescens 06 1151 EGS	17/06/2008 10:05
Pseudomonas fluorescens B148 UFL	30/01/2007 18:07
Pseudomonas fluorescens DSM 1976 DSM	21/09/2012 11:59
Pseudomonas fluorescens DSM 50090T HAM	17/01/2007 16:47
Pseudomonas fluorescens DSM 50154 DSM	21/09/2012 11:59
Pseudomonas fluorescens Group 5 PIM	16/07/2010 9:33
Pseudomonas fragi DSM 3456T HAM	17/01/2007 16:48
Pseudomonas frederiksbergensis DSM 13022T...	17/01/2007 16:48
Pseudomonas fulva 013 W30 NFI	17/06/2008 10:06
Pseudomonas fulva LMG 11722T HAM	17/01/2007 16:49
Pseudomonas fuscovaginae DSM 7231T HAM	17/01/2007 16:50
Pseudomonas gessardii CIP 105469T HAM	17/01/2007 16:51

MSP Name	Creation Date
Pseudomonas graminis DSM 11363T HAM	17/01/2007 16:51
Pseudomonas grimontii CIP 106645T HAM	17/01/2007 16:52
Pseudomonas indica DSM 14015T HAM	17/01/2007 16:54
Pseudomonas jessenii CIP 105274T HAM	17/01/2007 16:55
Pseudomonas jinjuensis LMG 21316T HAM	17/01/2007 16:56
Pseudomonas kilonensis DSM 13647T HAM	17/01/2007 16:56
Pseudomonas koreensis 037 W01 NFI	17/06/2008 10:06
Pseudomonas koreensis 2 2 TUB	16/02/2010 13:07
Pseudomonas koreensis LMG 21318T HAM	17/01/2007 16:57
Pseudomonas libanensis CIP 105460T HAM	17/01/2007 16:57
Pseudomonas lundensis DSM 6252T HAM	17/01/2007 16:58
Pseudomonas lutea LMG 21974T HAM	17/01/2007 16:59
Pseudomonas luteola 0807M14008901 IBS	30/03/2009 13:27
Pseudomonas luteola DSM 6975T HAM	17/01/2007 16:59
Pseudomonas luteola VA 00501 09 ERL	01/09/2009 13:26
Pseudomonas mandelii CIP 105273T HAM	17/01/2007 17:00
Pseudomonas marginalis DSM 13124T HAM	17/01/2007 17:00
Pseudomonas mendocina DSM 50017T HAM	17/01/2007 17:01
Pseudomonas migulae CIP 105470T HAM	17/01/2007 17:03
Pseudomonas monteilii 014 W29 NFI	17/06/2008 10:06
Pseudomonas monteilii 025 W23 NFI	17/06/2008 10:06
Pseudomonas monteilii 035 W14 NFI	17/06/2008 10:06
Pseudomonas monteilii 041 W10 NFI	17/06/2008 10:06
Pseudomonas monteilii 044 W06 NFI	17/06/2008 10:07
Pseudomonas monteilii DSM 14164T HAM	17/01/2007 17:03
Pseudomonas mosselii CIP 105259T HAM	17/01/2007 17:04
Pseudomonas mucidolens LMG 2223T HAM	17/01/2007 17:05
Pseudomonas nitroreducens DSM 14399T HAM	17/01/2007 17:06
Pseudomonas nitroreducens LMG 20221T HAM	17/01/2007 17:05
Pseudomonas oleovorans 062 Galv10 NFI	17/06/2008 10:07
Pseudomonas oleovorans B396 UFL	30/01/2007 18:08
Pseudomonas oleovorans DSM 1045T HAM	17/01/2007 17:07
Pseudomonas orientalis CIP 105540T HAM	17/01/2007 17:07
Pseudomonas oryzihabitans CCUG 31383 CCUG	21/09/2012 11:59
Pseudomonas oryzihabitans CCUG 46912 CCUG	21/09/2012 11:59
Pseudomonas oryzihabitans CCUG 51430 CCUG	21/09/2012 11:59
Pseudomonas oryzihabitans CCUG 60244 CCUG	21/09/2012 11:59
Pseudomonas oryzihabitans CCUG 9468 CCUG	21/09/2012 11:59
Pseudomonas oryzihabitans DSM 6835T DSM	21/09/2012 11:59
Pseudomonas oryzihabitans DSM 6835T HAM	17/01/2007 17:08
Pseudomonas otitidis 18 PIM	16/07/2010 9:19
Pseudomonas panipatensis DSM 21819T LGL	02/02/2012 8:04
Pseudomonas pertucinogena LMG 1874T HAM	17/01/2007 17:09
Pseudomonas pictorum LMG 981T HAM	17/01/2007 17:09
Pseudomonas plecoglossicida 023 W22 NFI	17/06/2008 10:07
Pseudomonas plecoglossicida 027 W28 NFI	17/06/2008 10:07
Pseudomonas plecoglossicida 042 W03 NFI	17/06/2008 10:07
Pseudomonas plecoglossicida CIP 106494 CIP	21/09/2012 11:59

MSP Name	Creation Date
Pseudomonas plecoglossicida CIP 106495 CIP	21/09/2012 11:59
Pseudomonas plecoglossicida DSM 15088T H...	17/01/2007 17:10
Pseudomonas poae DSM 14936T HAM	17/01/2007 17:11
Pseudomonas pohangensis DSM 17875T DSM	17/06/2008 10:07
Pseudomonas proteolytica DSM 15321T HAM	17/01/2007 17:11
Pseudomonas pseudoalcaligenes ssp pseudoal...	17/01/2007 17:12
Pseudomonas putida 039 W07 NFI	17/06/2008 10:07
Pseudomonas putida ATCC 49128 THL	17/01/2007 17:12
Pseudomonas putida B223 UFL	30/01/2007 18:09
Pseudomonas putida B317 UFL	30/01/2007 18:09
Pseudomonas putida B318 UFL	30/01/2007 18:09
Pseudomonas putida B319 UFL	30/01/2007 18:10
Pseudomonas putida B320 UFL	30/01/2007 18:10
Pseudomonas putida B342T UFL	17/01/2007 17:13
Pseudomonas putida B400 UFL	17/01/2007 17:14
Pseudomonas putida B401 UFL	17/01/2007 17:14
Pseudomonas putida B402 UFL	30/01/2007 18:10
Pseudomonas putida B404 UFL	30/01/2007 18:11
Pseudomonas putida B409 UFL	17/01/2007 17:14
Pseudomonas putida B410 UFL	09/05/2007 12:19
Pseudomonas putida B411 UFL	17/01/2007 17:15
Pseudomonas putida B524 UFL	30/01/2007 18:11
Pseudomonas putida B535 Mut of B534 UFL	30/01/2007 18:12
Pseudomonas putida B565 UFL	30/01/2007 18:12
Pseudomonas putida DSM 291T HAM	17/01/2007 17:17
Pseudomonas putida DSM 3226 DSM	29/02/2012 12:29
Pseudomonas putida DSM 50198 DSM	21/09/2012 11:59
Pseudomonas putida DSM 50198 HAM	17/01/2007 17:18
Pseudomonas putida DSM 6125 DSM	21/09/2012 11:59
Pseudomonas putida Mu15117 1 CHB	24/01/2007 15:07
Pseudomonas putida Group 53 PIM	16/07/2010 8:48
Pseudomonas putida Group 7 PIM	16/07/2010 9:34
Pseudomonas resinovorans LMG 2274T HAM	17/01/2007 17:18
Pseudomonas rhizosphaerae LMG 21640T HAM	17/01/2007 17:19
Pseudomonas rhodesiae DSM 14020T HAM	17/01/2007 17:20
Pseudomonas savastanoi ssp savastanoi LMG ...	17/01/2007 17:21
Pseudomonas savastanoi ssp savastanoi LMG ...	17/01/2007 17:21
Pseudomonas segetis DSM 18913T DSM	17/06/2008 10:08
Pseudomonas sp 01 12605128 MVK	12/12/2007 16:42
Pseudomonas sp 057 Galv13 NFI	17/06/2008 10:08
Pseudomonas sp 107 Neb26 NFI	17/06/2008 10:08
Pseudomonas sp 10w369720 RLH	10/03/2011 15:56
Pseudomonas sp B153 UFL	30/01/2007 18:12
Pseudomonas sp B538 UFL	17/01/2007 17:22
Pseudomonas sp B627 UFL	17/01/2007 17:23
Pseudomonas sp. SVBP6	15/12/2015 21:52
Pseudomonas sp[2] 991600314 LBK	01/09/2009 12:09
Pseudomonas straminea CIP 106745T HAM	17/01/2007 17:23

MSP Name	Creation Date
Pseudomonas stutzeri 040 W09 NFI	17/06/2008 10:08
Pseudomonas stutzeri 043 W05 NFI	17/06/2008 10:08
Pseudomonas stutzeri B367 UFL	17/01/2007 17:24
Pseudomonas stutzeri BK 02099 09 ERL	01/09/2009 14:23
Pseudomonas stutzeri DSM 13592 HAM	17/01/2007 16:40
Pseudomonas stutzeri DSM 17083 DSM	21/09/2012 11:59
Pseudomonas stutzeri DSM 5190T HAM	17/01/2007 17:25
Pseudomonas stutzeri DSM 6082 DSM	21/09/2012 11:59
Pseudomonas stutzeri DSM 6084 DSM	21/09/2012 11:59
Pseudomonas stutzeri V319 MCRF	04/03/2012 9:37
Pseudomonas synxantha DSM 18928T DSM	04/08/2010 16:27
Pseudomonas syringae ssp syringae DSM 669...	17/01/2007 17:26
Pseudomonas syringae ssp syringae LMG 1247...	17/01/2007 17:26
Pseudomonas taetrolens LMG 2336T HAM	17/01/2007 17:27
Pseudomonas thermotolerans DSM 14292T H...	17/01/2007 17:28
Pseudomonas thivervalensis DSM 13194T HAM	17/01/2007 17:28
Pseudomonas tolaasii LMG 2342T HAM	17/01/2007 17:29
Pseudomonas trivialis DSM 14937T HAM	17/01/2007 17:29
Pseudomonas umsongensis LMG 21317T HAM	17/01/2007 17:30
Pseudomonas vancouverensis CIP 106707T H...	17/01/2007 17:31
Pseudomonas veronii B559 UFL	17/01/2007 17:31
Pseudomonas veronii B560 UFL	17/01/2007 17:32
Pseudomonas veronii B561 UFL	17/01/2007 17:32
Pseudomonas veronii DSM 11331T HAM	17/01/2007 17:33
Pseudomonas viridiflava DSM 11124T HAM	17/01/2007 17:33
Pseudomonas xanthomarina DSM 18231T LGL	02/02/2012 8:05
