# Supplementary material for: Genomic insights into the broad antifungal activity, plant-probiotic properties, and their regulation, in Pseudomonas donghuensis strain SVBP6
Source: PLoS One. 2018 Mar 14;13(3):e0194088. doi: 10.1371/journal.pone.0194088 (PMC5851621; doi:10.1371/journal.pone.0194088)
Supplement: S2 Table — Genes in the reference genome of Pseudomonas aeruginosa PAO1 (Assembly Acc. GCA_000006765.1) are indicated. (PDF) [file pone.0194088.s002.pdf]

Gene	*Pseudomonas aeruginosa* PAO1 (GCA_000006765.1)		
	**Protein Id**	**Location**	**Locus tag**
rpsA	*AAG06550.1*	*complement(3548110..3549789)*	PA3162
rpsB	*AAG07044.1*	*complement(4094167..4094907)*	PA3656
rpsC	*AAG07645.1*	*complement(4763544..4764230)*	PA4257
rpsD	*AAG07627.1*	*complement(4755447..4756067)*	PA4239
rpsE	*AAG07634.1*	*complement(4759070..4759570)*	PA4246
rpsF	*AAG08320.1*	*complement(5537319..5537738)*	PA4935
rpsG	*AAG07655.1*	*complement(4771186..4771656)*	PA4267
rpsH	*AAG07637.1*	*complement(4760480..4760872)*	PA4249
rpsI	*AAG07820.1*	*complement(4965109..4965501)*	PA4432
rpsJ	*AAG07652.1*	*complement(4767343..4767654)*	PA4264
rpsK	*AAG07628.1*	*complement(4756084..4756473)*	PA4240
rpsM	*AAG07629.1*	*complement(4756492..4756848)*	PA4241
rpsN	*AAG07638.1*	*complement(4761062..4761367)*	PA4250
rpsO	*AAG08127.1*	*complement(5325653..5325922)*	PA4741
rpsP	*AAG07132.1*	*complement(4196708..4196959)*	PA3745
rpsQ	*AAG07642.1*	*complement(4762659..4762925)*	PA4254
rpsR	*AAG08319.1*	*complement(5537059..5537289)*	PA4934
rpsS	*AAG07647.1*	*complement(4764588..4764863)*	PA4259
rpsT	*AAG07951.1*	*5112663..5112938*	PA4563
rplA	*AAG07661.1*	*complement(4781985..4782680)*	PA4273
rplB	*AAG07648.1*	*complement(4764880..4765701)*	PA4260
rplC	*AAG07651.1*	*complement(4766625..4767260)*	PA4263
rplD	*AAG07650.1*	*complement(4766009..4766611)*	PA4262
rplE	*AAG07639.1*	*complement(4761381..4761920)*	PA4251
rplF	*AAG07636.1*	*complement(4759935..4760468)*	PA4248
rplI	*AAG08317.1*	*complement(5535685..5536131)*	PA4932

rplJ	*AAG07660.1*	*complement(4781286..4781786)*	PA4272
rplK	*AAG07662.1*	*complement(4782680..4783111)*	PA4274
rplL	*AAG07659.1*	*complement(4780839..4781207)*	PA4271
rplM	*AAG07821.1*	*complement(4965516..4965944)*	PA4433
rplN	*AAG07641.1*	*complement(4762267..4762635)*	PA4253
rplP	*AAG07644.1*	*complement(4763119..4763532)*	PA4256
rplQ	*AAG07625.1*	*complement(4753990..4754379)*	PA4237
rplR	*AAG07635.1*	*complement(4759574..4759924)*	PA4247
rplS	*AAG07129.1*	*complement(4195008..4195358)*	PA3742
rplT	*AAG06129.1*	*complement(3103643..3103999)*	PA2741
rplU	*AAG07956.1*	*complement(5116313..5116624)*	PA4568
rplX	*AAG07640.1*	*complement(4761940..4762254)*	PA4252
rpmA	*AAG07955.1*	*complement(5116032..5116289)*	PA4567
rpmB	*AAG08701.1*	*complement(5985883..5986119)*	PA5316
rpmC	*AAG07643.1*	*complement(4762928..4763119)*	PA4255
rpmE	*AAG08434.1*	*complement(5687105..5687320)*	PA5049
rpmG	*AAG08700.1*	*complement(5985716..5985871)*	PA5315
rpmI	*AAG06130.1*	*complement(3104023..3104217)*	PA2742
