# Supplementary material for: Genomic insights into the broad antifungal activity, plant-probiotic properties, and their regulation, in Pseudomonas donghuensis strain SVBP6
Source: PLoS One. 2018 Mar 14;13(3):e0194088. doi: 10.1371/journal.pone.0194088 (PMC5851621; doi:10.1371/journal.pone.0194088)
Supplement: S3 Table — (PDF) [file pone.0194088.s003.pdf]

Strain Name	Assembly Accession Code	Cluster	Sampled Genomes (Representatives of genetic diversity) [a]	Closely related genomes to SVBP6 (Putida group) [b]
Pseudomonas sp. SVBP6	-		x	x
Pseudomonas_otitidis	*GCF_000632755.1*	*0*	x	
Pseudomonas_aeruginosa	*GCA_000698765.1*	*0*		
Pseudomonas_aeruginosa	*GCA_000709285.1*	*0*		
Pseudomonas_aeruginosa	*GCA_000710625.1*	*0*		
Pseudomonas_aeruginosa	*GCA_000743405.1*	*0*		
Pseudomonas_aeruginosa	*GCA_000756575.1*	*0*		
Pseudomonas_aeruginosa	*GCA_000760505.2*	*0*		
Pseudomonas_aeruginosa	*GCA_000786565.1*	*0*		
Pseudomonas_aeruginosa	*GCA_000816985.1*	*0*		
Pseudomonas_aeruginosa	*GCA_000829885.1*	*0*		
Pseudomonas_aeruginosa	*GCA_000937465.2*	*0*		
Pseudomonas_aeruginosa	*GCA_000937495.2*	*0*		
Pseudomonas_aeruginosa	*GCA_000981825.1*	*0*		
Pseudomonas_aeruginosa	*GCA_000988505.1*	*0*		
Pseudomonas_aeruginosa	*GCA_001013395.1*	*0*		
Pseudomonas_aeruginosa	*GCA_001028745.1*	*0*		
Pseudomonas_aeruginosa	*GCA_001042925.1*	*0*		
Pseudomonas_aeruginosa	*GCA_001077475.1*	*0*		
Pseudomonas_aeruginosa	*GCA_001180345.1*	*0*		
Pseudomonas_aeruginosa	*GCA_001180365.1*	*0*		
Pseudomonas_aeruginosa	*GCA_001180385.1*	*0*		
Pseudomonas_aeruginosa	*GCA_001180405.1*	*0*		
Pseudomonas_aeruginosa	*GCA_001180445.1*	*0*		
Pseudomonas_aeruginosa	*GCA_001180465.1*	*0*		
Pseudomonas_aeruginosa	*GCA_001180485.1*	*0*		
Pseudomonas_aeruginosa	*GCA_001180505.1*	*0*		
Pseudomonas_aeruginosa	*GCA_001180525.1*	*0*		
Pseudomonas_aeruginosa	*GCA_001180545.1*	*0*		
Pseudomonas_aeruginosa	*GCA_001180565.1*	*0*		
Pseudomonas_aeruginosa	*GCA_001180585.1*	*0*		
Pseudomonas_aeruginosa	*GCA_001180605.1*	*0*		
Pseudomonas_aeruginosa	*GCA_001180625.1*	*0*		
Pseudomonas_aeruginosa	*GCA_001180645.1*	*0*		
Pseudomonas_aeruginosa	*GCA_001180665.1*	*0*		
Pseudomonas_aeruginosa	*GCA_001180685.1*	*0*		
Pseudomonas_aeruginosa	*GCA_001180705.1*	*0*		
Pseudomonas_aeruginosa	*GCA_001180725.1*	*0*		
Pseudomonas_aeruginosa	*GCA_001180745.1*	*0*		
Pseudomonas_aeruginosa	*GCA_001180785.1*	*0*		
Pseudomonas_aeruginosa	*GCA_001180805.1*	*0*		
Pseudomonas_aeruginosa	*GCA_001180825.1*	*0*		
Pseudomonas_aeruginosa	*GCA_001180845.1*	*0*		
Pseudomonas_aeruginosa	*GCA_001180865.1*	*0*		
Pseudomonas_aeruginosa	*GCA_001180885.1*	*0*		
Pseudomonas_aeruginosa	*GCA_001180905.1*	*0*		
Pseudomonas_aeruginosa	*GCA_001180925.1*	*0*		
Pseudomonas_aeruginosa	*GCA_001180945.1*	*0*		
Pseudomonas_aeruginosa	*GCA_001180965.1*	*0*		
Pseudomonas_aeruginosa	*GCA_001180985.1*	*0*		
Pseudomonas_aeruginosa	*GCA_001181005.1*	*0*		
Pseudomonas_aeruginosa	*GCA_001181025.1*	*0*		

Pseudomonas_aeruginosa	*GCA_001181045.1*	*0*	
Pseudomonas_aeruginosa	*GCA_001181065.1*	*0*	
Pseudomonas_aeruginosa	*GCA_001181085.1*	*0*	
Pseudomonas_aeruginosa	*GCA_001181105.1*	*0*	
Pseudomonas_aeruginosa	*GCA_001181125.1*	*0*	
Pseudomonas_aeruginosa	*GCA_001181145.1*	*0*	
Pseudomonas_aeruginosa	*GCA_001181165.1*	*0*	
Pseudomonas_aeruginosa	*GCA_001181185.1*	*0*	
Pseudomonas_aeruginosa	*GCA_001181205.1*	*0*	
Pseudomonas_aeruginosa	*GCA_001181225.1*	*0*	
Pseudomonas_aeruginosa	*GCA_001181245.1*	*0*	
Pseudomonas_aeruginosa	*GCA_001181265.1*	*0*	
Pseudomonas_aeruginosa	*GCA_001181285.1*	*0*	
Pseudomonas_aeruginosa	*GCA_001181305.1*	*0*	
Pseudomonas_aeruginosa	*GCA_001181345.1*	*0*	
Pseudomonas_aeruginosa	*GCA_001181365.1*	*0*	
Pseudomonas_aeruginosa	*GCA_001181385.1*	*0*	
Pseudomonas_aeruginosa	*GCA_001181425.1*	*0*	
Pseudomonas_aeruginosa	*GCA_001181445.1*	*0*	
Pseudomonas_aeruginosa	*GCA_001181465.1*	*0*	
Pseudomonas_aeruginosa	*GCA_001181485.1*	*0*	
Pseudomonas_aeruginosa	*GCA_001181525.1*	*0*	
Pseudomonas_aeruginosa	*GCA_001181545.1*	*0*	
Pseudomonas_aeruginosa	*GCA_001181565.1*	*0*	
Pseudomonas_aeruginosa	*GCA_001181585.1*	*0*	
Pseudomonas_aeruginosa	*GCA_001181645.1*	*0*	
Pseudomonas_aeruginosa	*GCA_001181665.1*	*0*	
Pseudomonas_aeruginosa	*GCA_001181685.1*	*0*	
Pseudomonas_aeruginosa	*GCA_001181725.1*	*0*	
Pseudomonas_aeruginosa	*GCA_001181745.1*	*0*	
Pseudomonas_aeruginosa	*GCA_001181765.1*	*0*	
Pseudomonas_aeruginosa	*GCA_001181785.1*	*0*	
Pseudomonas_aeruginosa	*GCA_001181805.1*	*0*	
Pseudomonas_aeruginosa	*GCA_001181825.1*	*0*	
Pseudomonas_aeruginosa	*GCA_001181845.1*	*0*	
Pseudomonas_aeruginosa	*GCA_001181865.1*	*0*	
Pseudomonas_aeruginosa	*GCA_001181885.1*	*0*	
Pseudomonas_aeruginosa	*GCA_001181905.1*	*0*	
Pseudomonas_aeruginosa	*GCA_001181925.1*	*0*	
Pseudomonas_aeruginosa	*GCA_001181945.1*	*0*	
Pseudomonas_aeruginosa	*GCA_001181965.1*	*0*	
Pseudomonas_aeruginosa	*GCA_001182005.1*	*0*	
Pseudomonas_aeruginosa	*GCA_001182025.1*	*0*	
Pseudomonas_aeruginosa	*GCA_001182945.1*	*0*	
Pseudomonas_aeruginosa	*GCA_001293085.1*	*0*	
Pseudomonas_aeruginosa	*GCA_001294675.1*	*0*	
Pseudomonas_aeruginosa	*GCA_001295275.1*	*0*	
Pseudomonas_aeruginosa	*GCA_001295285.1*	*0*	
Pseudomonas_aeruginosa	*GCA_001295295.1*	*0*	
Pseudomonas_aeruginosa	*GCA_001295345.1*	*0*	
Pseudomonas_aeruginosa	*GCA_001402955.1*	*0*	
Pseudomonas_aeruginosa	*GCA_001414085.1*	*0*	
Pseudomonas_aeruginosa	*GCA_001414105.1*	*0*	
Pseudomonas_aeruginosa	*GCA_001414155.1*	*0*	
Pseudomonas_aeruginosa	*GCA_001414165.1*	*0*	

Pseudomonas_aeruginosa	GCA_001414175.1	0	
Pseudomonas_aeruginosa	GCA_001420185.1	0	
Pseudomonas_aeruginosa	GCA_001420205.1	0	
Pseudomonas_aeruginosa	GCA_001420225.1	0	
Pseudomonas_aeruginosa	GCA_001420245.1	0	
Pseudomonas_aeruginosa	GCA_001420265.1	0	
Pseudomonas_aeruginosa	GCA_001420525.1	0	
Pseudomonas_aeruginosa	GCA_001420535.1	0	
Pseudomonas_aeruginosa	GCA_001444745.1	0	
Pseudomonas_aeruginosa	GCA_001444755.1	0	
Pseudomonas_aeruginosa	GCA_001444765.1	0	
Pseudomonas_aeruginosa	GCA_001444815.1	0	
Pseudomonas_aeruginosa	GCA_001444835.1	0	
Pseudomonas_aeruginosa	GCA_001444845.1	0	
Pseudomonas_aeruginosa	GCA_001444865.1	0	
Pseudomonas_aeruginosa	GCA_001444895.1	0	
Pseudomonas_aeruginosa	GCA_001444915.1	0	
Pseudomonas_aeruginosa	GCA_001444925.1	0	
Pseudomonas_aeruginosa	GCA_001444955.1	0	
Pseudomonas_aeruginosa	GCA_001444975.1	0	
Pseudomonas_aeruginosa	GCA_001444995.1	0	
Pseudomonas_aeruginosa	GCA_001445005.1	0	
Pseudomonas_aeruginosa	GCA_001445035.1	0	
Pseudomonas_aeruginosa	GCA_001447845.1	0	
Pseudomonas_aeruginosa	GCA_001449145.1	0	
Pseudomonas_aeruginosa	GCA_001449155.1	0	
Pseudomonas_aeruginosa	GCA_001449165.1	0	
Pseudomonas_aeruginosa	GCA_001449175.1	0	
Pseudomonas_aeruginosa	GCA_001449225.1	0	
Pseudomonas_aeruginosa	GCA_001449245.1	0	
Pseudomonas_aeruginosa	GCA_001449255.1	0	
Pseudomonas_aeruginosa	GCA_001449275.1	0	
Pseudomonas_aeruginosa	GCA_001449305.1	0	
Pseudomonas_aeruginosa	GCA_001449325.1	0	
Pseudomonas_aeruginosa	GCA_001449345.1	0	
Pseudomonas_aeruginosa	GCA_001449365.1	0	
Pseudomonas_aeruginosa	GCA_001449385.1	0	
Pseudomonas_aeruginosa	GCA_001449395.1	0	
Pseudomonas_aeruginosa	GCA_001449425.1	0	
Pseudomonas_aeruginosa	GCA_001449435.1	0	
Pseudomonas_aeruginosa	GCA_001449465.1	0	
Pseudomonas_aeruginosa	GCA_001449485.1	0	
Pseudomonas_aeruginosa	GCA_001449505.1	0	
Pseudomonas_aeruginosa	GCA_001449515.1	0	
Pseudomonas_aeruginosa	GCA_001449545.1	0	
Pseudomonas_aeruginosa	GCA_001449555.1	0	
Pseudomonas_aeruginosa	GCA_001449585.1	0	
Pseudomonas_aeruginosa	GCA_001449605.1	0	
Pseudomonas_aeruginosa	GCA_001449625.1	0	
Pseudomonas_aeruginosa	GCA_001449645.1	0	
Pseudomonas_aeruginosa	GCA_001449655.1	0	
Pseudomonas_aeruginosa	GCA_001449675.1	0	
Pseudomonas_aeruginosa	GCA_001449705.1	0	
Pseudomonas_aeruginosa	GCA_001449725.1	0	
Pseudomonas_aeruginosa	GCA_001449735.1	0	

Pseudomonas_aeruginosa	GCA_001449765.1	0	
Pseudomonas_aeruginosa	GCA_001449785.1	0	
Pseudomonas_aeruginosa	GCA_001449805.1	0	
Pseudomonas_aeruginosa	GCA_001449825.1	0	
Pseudomonas_aeruginosa	GCA_001449835.1	0	
Pseudomonas_aeruginosa	GCA_001449865.1	0	
Pseudomonas_aeruginosa	GCA_001449885.1	0	
Pseudomonas_aeruginosa	GCA_001449905.1	0	
Pseudomonas_aeruginosa	GCA_001449925.1	0	
Pseudomonas_aeruginosa	GCA_001449935.1	0	
Pseudomonas_aeruginosa	GCA_001449965.1	0	
Pseudomonas_aeruginosa	GCA_001449985.1	0	
Pseudomonas_aeruginosa	GCA_001450005.1	0	
Pseudomonas_aeruginosa	GCA_001450015.1	0	
Pseudomonas_aeruginosa	GCA_001450045.1	0	
Pseudomonas_aeruginosa	GCA_001450065.1	0	
Pseudomonas_aeruginosa	GCA_001450085.1	0	
Pseudomonas_aeruginosa	GCA_001450095.1	0	
Pseudomonas_aeruginosa	GCA_001450115.1	0	
Pseudomonas_aeruginosa	GCA_001450145.1	0	
Pseudomonas_aeruginosa	GCA_001450165.1	0	
Pseudomonas_aeruginosa	GCA_001450185.1	0	
Pseudomonas_aeruginosa	GCA_001450195.1	0	
Pseudomonas_aeruginosa	GCA_001450225.1	0	
Pseudomonas_aeruginosa	GCA_001450245.1	0	
Pseudomonas_aeruginosa	GCA_001450265.1	0	
Pseudomonas_aeruginosa	GCA_001450275.1	0	
Pseudomonas_aeruginosa	GCA_001450305.1	0	
Pseudomonas_aeruginosa	GCA_001450325.1	0	
Pseudomonas_aeruginosa	GCA_001450345.1	0	
Pseudomonas_aeruginosa	GCA_001450355.1	0	
Pseudomonas_aeruginosa	GCA_001450385.1	0	
Pseudomonas_aeruginosa	GCA_001450405.1	0	
Pseudomonas_aeruginosa	GCA_001450425.1	0	
Pseudomonas_aeruginosa	GCA_001450435.1	0	
Pseudomonas_aeruginosa	GCA_001450465.1	0	
Pseudomonas_aeruginosa	GCA_001450485.1	0	
Pseudomonas_aeruginosa	GCA_001450505.1	0	
Pseudomonas_aeruginosa	GCA_001450525.1	0	
Pseudomonas_aeruginosa	GCA_001450545.1	0	
Pseudomonas_aeruginosa	GCA_001450555.1	0	
Pseudomonas_aeruginosa	GCA_001450585.1	0	
Pseudomonas_aeruginosa	GCA_001450605.1	0	
Pseudomonas_aeruginosa	GCA_001450625.1	0	
Pseudomonas_aeruginosa	GCA_001450635.1	0	
Pseudomonas_aeruginosa	GCA_001450665.1	0	
Pseudomonas_aeruginosa	GCA_001450675.1	0	
Pseudomonas_aeruginosa	GCA_001450705.1	0	
Pseudomonas_aeruginosa	GCA_001450715.1	0	
Pseudomonas_aeruginosa	GCA_001450745.1	0	
Pseudomonas_aeruginosa	GCA_001450755.1	0	
Pseudomonas_aeruginosa	GCA_001450785.1	0	
Pseudomonas_aeruginosa	GCA_001450795.1	0	
Pseudomonas_aeruginosa	GCA_001450825.1	0	
Pseudomonas_aeruginosa	GCA_001450835.1	0	

Pseudomonas_aeruginosa	GCA_001450865.1	0
Pseudomonas_aeruginosa	GCA_001450875.1	0
Pseudomonas_aeruginosa	GCA_001450905.1	0
Pseudomonas_aeruginosa	GCA_001450915.1	0
Pseudomonas_aeruginosa	GCA_001450925.1	0
Pseudomonas_aeruginosa	GCA_001450935.1	0
Pseudomonas_aeruginosa	GCA_001450985.1	0
Pseudomonas_aeruginosa	GCA_001450995.1	0
Pseudomonas_aeruginosa	GCA_001451015.1	0
Pseudomonas_aeruginosa	GCA_001451025.1	0
Pseudomonas_aeruginosa	GCA_001451065.1	0
Pseudomonas_aeruginosa	GCA_001451075.1	0
Pseudomonas_aeruginosa	GCA_001451105.1	0
Pseudomonas_aeruginosa	GCA_001451125.1	0
Pseudomonas_aeruginosa	GCA_001451145.1	0
Pseudomonas_aeruginosa	GCA_001451165.1	0
Pseudomonas_aeruginosa	GCA_001451175.1	0
Pseudomonas_aeruginosa	GCA_001451205.1	0
Pseudomonas_aeruginosa	GCA_001451225.1	0
Pseudomonas_aeruginosa	GCA_001451245.1	0
Pseudomonas_aeruginosa	GCA_001451255.1	0
Pseudomonas_aeruginosa	GCA_001451265.1	0
Pseudomonas_aeruginosa	GCA_001451295.1	0
Pseudomonas_aeruginosa	GCA_001451325.1	0
Pseudomonas_aeruginosa	GCA_001451335.1	0
Pseudomonas_aeruginosa	GCA_001451355.1	0
Pseudomonas_aeruginosa	GCA_001451375.1	0
Pseudomonas_aeruginosa	GCA_001451405.1	0
Pseudomonas_aeruginosa	GCA_001451425.1	0
Pseudomonas_aeruginosa	GCA_001451445.1	0
Pseudomonas_aeruginosa	GCA_001451455.1	0
Pseudomonas_aeruginosa	GCA_001451485.1	0
Pseudomonas_aeruginosa	GCA_001451505.1	0
Pseudomonas_aeruginosa	GCA_001451525.1	0
Pseudomonas_aeruginosa	GCA_001451535.1	0
Pseudomonas_aeruginosa	GCA_001451565.1	0
Pseudomonas_aeruginosa	GCA_001451575.1	0
Pseudomonas_aeruginosa	GCA_001451605.1	0
Pseudomonas_aeruginosa	GCA_001451625.1	0
Pseudomonas_aeruginosa	GCA_001451655.1	0
Pseudomonas_aeruginosa	GCA_001451685.1	0
Pseudomonas_aeruginosa	GCA_001451705.1	0
Pseudomonas_aeruginosa	GCA_001451725.1	0
Pseudomonas_aeruginosa	GCA_001451745.1	0
Pseudomonas_aeruginosa	GCA_001451755.1	0
Pseudomonas_aeruginosa	GCA_001451785.1	0
Pseudomonas_aeruginosa	GCA_001451805.1	0
Pseudomonas_aeruginosa	GCA_001451815.1	0
Pseudomonas_aeruginosa	GCA_001451835.1	0
Pseudomonas_aeruginosa	GCA_001451865.1	0
Pseudomonas_aeruginosa	GCA_001451885.1	0
Pseudomonas_aeruginosa	GCA_001451895.1	0
Pseudomonas_aeruginosa	GCA_001451905.1	0
Pseudomonas_aeruginosa	GCA_001451945.1	0
Pseudomonas_aeruginosa	GCA_001451965.1	0

Pseudomonas_aeruginosa	*GCA_001451975.1*	*0*
Pseudomonas_aeruginosa	*GCA_001452025.1*	*0*
Pseudomonas_aeruginosa	*GCA_001452045.1*	*0*
Pseudomonas_aeruginosa	*GCA_001452055.1*	*0*
Pseudomonas_aeruginosa	*GCA_001452075.1*	*0*
Pseudomonas_aeruginosa	*GCA_001452105.1*	*0*
Pseudomonas_aeruginosa	*GCA_001452125.1*	*0*
Pseudomonas_aeruginosa	*GCA_001452135.1*	*0*
Pseudomonas_aeruginosa	*GCA_001452155.1*	*0*
Pseudomonas_aeruginosa	*GCA_001452185.1*	*0*
Pseudomonas_aeruginosa	*GCA_001452205.1*	*0*
Pseudomonas_aeruginosa	*GCA_001452215.1*	*0*
Pseudomonas_aeruginosa	*GCA_001452235.1*	*0*
Pseudomonas_aeruginosa	*GCA_001452255.1*	*0*
Pseudomonas_aeruginosa	*GCA_001452285.1*	*0*
Pseudomonas_aeruginosa	*GCA_001452305.1*	*0*
Pseudomonas_aeruginosa	*GCA_001452325.1*	*0*
Pseudomonas_aeruginosa	*GCA_001452335.1*	*0*
Pseudomonas_aeruginosa	*GCA_001452365.1*	*0*
Pseudomonas_aeruginosa	*GCA_001452385.1*	*0*
Pseudomonas_aeruginosa	*GCA_001452405.1*	*0*
Pseudomonas_aeruginosa	*GCA_001452415.1*	*0*
Pseudomonas_aeruginosa	*GCA_001452445.1*	*0*
Pseudomonas_aeruginosa	*GCA_001452465.1*	*0*
Pseudomonas_aeruginosa	*GCA_001452485.1*	*0*
Pseudomonas_aeruginosa	*GCA_001452495.1*	*0*
Pseudomonas_aeruginosa	*GCA_001452525.1*	*0*
Pseudomonas_aeruginosa	*GCA_001452545.1*	*0*
Pseudomonas_aeruginosa	*GCA_001452565.1*	*0*
Pseudomonas_aeruginosa	*GCA_001452575.1*	*0*
Pseudomonas_aeruginosa	*GCA_001452605.1*	*0*
Pseudomonas_aeruginosa	*GCA_001452615.1*	*0*
Pseudomonas_aeruginosa	*GCA_001452645.1*	*0*
Pseudomonas_aeruginosa	*GCA_001452665.1*	*0*
Pseudomonas_aeruginosa	*GCA_001452675.1*	*0*
Pseudomonas_aeruginosa	*GCA_001452725.1*	*0*
Pseudomonas_aeruginosa	*GCA_001452745.1*	*0*
Pseudomonas_aeruginosa	*GCA_001452755.1*	*0*
Pseudomonas_aeruginosa	*GCA_001452765.1*	*0*
Pseudomonas_aeruginosa	*GCA_001452805.1*	*0*
Pseudomonas_aeruginosa	*GCA_001452825.1*	*0*
Pseudomonas_aeruginosa	*GCA_001452835.1*	*0*
Pseudomonas_aeruginosa	*GCA_001452865.1*	*0*
Pseudomonas_aeruginosa	*GCA_001452885.1*	*0*
Pseudomonas_aeruginosa	*GCA_001452905.1*	*0*
Pseudomonas_aeruginosa	*GCA_001452915.1*	*0*
Pseudomonas_aeruginosa	*GCA_001452935.1*	*0*
Pseudomonas_aeruginosa	*GCA_001452965.1*	*0*
Pseudomonas_aeruginosa	*GCA_001452985.1*	*0*
Pseudomonas_aeruginosa	*GCA_001452995.1*	*0*
Pseudomonas_aeruginosa	*GCA_001453015.1*	*0*
Pseudomonas_aeruginosa	*GCA_001453065.1*	*0*
Pseudomonas_aeruginosa	*GCA_001453075.1*	*0*
Pseudomonas_aeruginosa	*GCA_001453095.1*	*0*
Pseudomonas_aeruginosa	*GCA_001453125.1*	*0*

Pseudomonas_aeruginosa	*GCA_001453145.1*	*0*	
Pseudomonas_aeruginosa	*GCA_001453155.1*	*0*	
Pseudomonas_aeruginosa	*GCA_001453185.1*	*0*	
Pseudomonas_aeruginosa	*GCA_001453205.1*	*0*	
Pseudomonas_aeruginosa	*GCA_001453225.1*	*0*	
Pseudomonas_aeruginosa	*GCA_001453235.1*	*0*	
Pseudomonas_aeruginosa	*GCA_001453265.1*	*0*	
Pseudomonas_aeruginosa	*GCA_001453285.1*	*0*	
Pseudomonas_aeruginosa	*GCA_001453305.1*	*0*	
Pseudomonas_aeruginosa	*GCA_001453315.1*	*0*	
Pseudomonas_aeruginosa	*GCA_001453345.1*	*0*	
Pseudomonas_aeruginosa	*GCA_001453365.1*	*0*	
Pseudomonas_aeruginosa	*GCA_001453385.1*	*0*	
Pseudomonas_aeruginosa	*GCA_001453405.1*	*0*	
Pseudomonas_aeruginosa	*GCA_001453415.1*	*0*	
Pseudomonas_aeruginosa	*GCA_001453435.1*	*0*	
Pseudomonas_aeruginosa	*GCA_001453465.1*	*0*	
Pseudomonas_aeruginosa	*GCA_001453485.1*	*0*	
Pseudomonas_aeruginosa	*GCA_001453495.1*	*0*	
Pseudomonas_aeruginosa	*GCA_001453515.1*	*0*	
Pseudomonas_aeruginosa	*GCA_001453545.1*	*0*	
Pseudomonas_aeruginosa	*GCA_001453565.1*	*0*	
Pseudomonas_aeruginosa	*GCA_001453585.1*	*0*	
Pseudomonas_aeruginosa	*GCA_001453595.1*	*0*	
Pseudomonas_aeruginosa	*GCA_001453615.1*	*0*	
Pseudomonas_aeruginosa	*GCA_001453645.1*	*0*	
Pseudomonas_aeruginosa	*GCA_001453665.1*	*0*	
Pseudomonas_aeruginosa	*GCA_001453675.1*	*0*	
Pseudomonas_aeruginosa	*GCA_001453705.1*	*0*	
Pseudomonas_aeruginosa	*GCA_001453725.1*	*0*	
Pseudomonas_aeruginosa	*GCA_001453735.1*	*0*	
Pseudomonas_aeruginosa	*GCA_001453755.1*	*0*	
Pseudomonas_aeruginosa	*GCA_001453775.1*	*0*	
Pseudomonas_aeruginosa	*GCA_001453805.1*	*0*	
Pseudomonas_aeruginosa	*GCA_001453825.1*	*0*	
Pseudomonas_aeruginosa	*GCA_001453835.1*	*0*	
Pseudomonas_aeruginosa	*GCA_001453855.1*	*0*	
Pseudomonas_aeruginosa	*GCA_001453885.1*	*0*	
Pseudomonas_aeruginosa	*GCA_001453905.1*	*0*	
Pseudomonas_aeruginosa	*GCA_001453915.1*	*0*	
Pseudomonas_aeruginosa	*GCA_001453945.1*	*0*	
Pseudomonas_aeruginosa	*GCA_001453985.1*	*0*	
Pseudomonas_aeruginosa	*GCA_001454045.1*	*0*	
Pseudomonas_aeruginosa	*GCA_001454065.1*	*0*	
Pseudomonas_aeruginosa	*GCA_001454085.1*	*0*	
Pseudomonas_aeruginosa	*GCA_001454095.1*	*0*	
Pseudomonas_aeruginosa	*GCA_001454125.1*	*0*	
Pseudomonas_aeruginosa	*GCA_001454145.1*	*0*	
Pseudomonas_aeruginosa	*GCA_001454165.1*	*0*	
Pseudomonas_aeruginosa	*GCA_001454175.1*	*0*	
Pseudomonas_aeruginosa	*GCA_001454205.1*	*0*	
Pseudomonas_aeruginosa	*GCA_001454225.1*	*0*	
Pseudomonas_aeruginosa	*GCA_001454235.1*	*0*	
Pseudomonas_aeruginosa	*GCA_001454285.1*	*0*	
Pseudomonas_aeruginosa	*GCA_001454305.1*	*0*	

Pseudomonas_aeruginosa	GCA_001454325.1	0
Pseudomonas_aeruginosa	GCA_001454335.1	0
Pseudomonas_aeruginosa	GCA_001454365.1	0
Pseudomonas_aeruginosa	GCA_001454385.1	0
Pseudomonas_aeruginosa	GCA_001454395.1	0
Pseudomonas_aeruginosa	GCA_001454415.1	0
Pseudomonas_aeruginosa	GCA_001454445.1	0
Pseudomonas_aeruginosa	GCA_001454495.1	0
Pseudomonas_aeruginosa	GCA_001454515.1	0
Pseudomonas_aeruginosa	GCA_001454545.1	0
Pseudomonas_aeruginosa	GCA_001454565.1	0
Pseudomonas_aeruginosa	GCA_001454585.1	0
Pseudomonas_aeruginosa	GCA_001454595.1	0
Pseudomonas_aeruginosa	GCA_001454625.1	0
Pseudomonas_aeruginosa	GCA_001454645.1	0
Pseudomonas_aeruginosa	GCA_001454665.1	0
Pseudomonas_aeruginosa	GCA_001454675.1	0
Pseudomonas_aeruginosa	GCA_001454705.1	0
Pseudomonas_aeruginosa	GCA_001454725.1	0
Pseudomonas_aeruginosa	GCA_001454745.1	0
Pseudomonas_aeruginosa	GCA_001454755.1	0
Pseudomonas_aeruginosa	GCA_001454785.1	0
Pseudomonas_aeruginosa	GCA_001454805.1	0
Pseudomonas_aeruginosa	GCA_001454825.1	0
Pseudomonas_aeruginosa	GCA_001469695.1	0
Pseudomonas_aeruginosa	GCA_001482325.1	0
Pseudomonas_aeruginosa	GCA_001483235.1	0
Pseudomonas_aeruginosa	GCA_001500245.1	0
Pseudomonas_aeruginosa	GCA_001515845.2	0
Pseudomonas_aeruginosa	GCA_001515915.2	0
Pseudomonas_aeruginosa	GCA_001516005.1	0
Pseudomonas_aeruginosa	GCA_001516105.1	0
Pseudomonas_aeruginosa	GCA_001516165.2	0
Pseudomonas_aeruginosa	GCA_001516185.2	0
Pseudomonas_aeruginosa	GCA_001516205.2	0
Pseudomonas_aeruginosa	GCA_001516225.2	0
Pseudomonas_aeruginosa	GCA_001516245.2	0
Pseudomonas_aeruginosa	GCA_001516265.1	0
Pseudomonas_aeruginosa	GCA_001516305.2	0
Pseudomonas_aeruginosa	GCA_001516325.2	0
Pseudomonas_aeruginosa	GCA_001516345.1	0
Pseudomonas_aeruginosa	GCA_001516365.2	0
Pseudomonas_aeruginosa	GCA_001516385.1	0
Pseudomonas_aeruginosa	GCA_001518975.1	0
Pseudomonas_aeruginosa	GCA_001542795.2	0
Pseudomonas_aeruginosa	GCA_001542835.1	0
Pseudomonas_aeruginosa	GCA_001545175.1	0
Pseudomonas_aeruginosa	GCA_001545185.1	0
Pseudomonas_aeruginosa	GCA_001545195.1	0
Pseudomonas_aeruginosa	GCA_001545205.1	0
Pseudomonas_aeruginosa	GCA_001545255.1	0
Pseudomonas_aeruginosa	GCA_001547955.1	0
Pseudomonas_aeruginosa	GCA_001548135.1	0
Pseudomonas_aeruginosa	GCA_001554305.1	0
Pseudomonas_aeruginosa	GCA_001554315.1	0

Pseudomonas_aeruginosa	*GCA_001554325.1*	*0*
Pseudomonas_aeruginosa	*GCA_001554375.1*	*0*
Pseudomonas_aeruginosa	*GCA_001554385.1*	*0*
Pseudomonas_aeruginosa	*GCA_001554395.1*	*0*
Pseudomonas_aeruginosa	*GCA_001554425.1*	*0*
Pseudomonas_aeruginosa	*GCA_001554455.1*	*0*
Pseudomonas_aeruginosa	*GCA_001554465.1*	*0*
Pseudomonas_aeruginosa	*GCA_001554495.1*	*0*
Pseudomonas_aeruginosa	*GCA_001554505.1*	*0*
Pseudomonas_aeruginosa	*GCA_001554535.1*	*0*
Pseudomonas_aeruginosa	*GCA_001554545.1*	*0*
Pseudomonas_aeruginosa	*GCA_001554575.1*	*0*
Pseudomonas_aeruginosa	*GCA_001554585.1*	*0*
Pseudomonas_aeruginosa	*GCA_001554615.1*	*0*
Pseudomonas_aeruginosa	*GCA_001554625.1*	*0*
Pseudomonas_aeruginosa	*GCA_001554655.1*	*0*
Pseudomonas_aeruginosa	*GCA_001554665.1*	*0*
Pseudomonas_aeruginosa	*GCA_001554695.1*	*0*
Pseudomonas_aeruginosa	*GCA_001554705.1*	*0*
Pseudomonas_aeruginosa	*GCA_001554735.1*	*0*
Pseudomonas_aeruginosa	*GCA_001554745.1*	*0*
Pseudomonas_aeruginosa	*GCA_001554775.1*	*0*
Pseudomonas_aeruginosa	*GCA_001554785.1*	*0*
Pseudomonas_aeruginosa	*GCA_001554805.1*	*0*
Pseudomonas_aeruginosa	*GCA_001554825.1*	*0*
Pseudomonas_aeruginosa	*GCA_001554855.1*	*0*
Pseudomonas_aeruginosa	*GCA_001554875.1*	*0*
Pseudomonas_aeruginosa	*GCA_001554895.1*	*0*
Pseudomonas_aeruginosa	*GCA_001554915.1*	*0*
Pseudomonas_aeruginosa	*GCA_001554935.1*	*0*
Pseudomonas_aeruginosa	*GCA_001554955.1*	*0*
Pseudomonas_aeruginosa	*GCA_001554975.1*	*0*
Pseudomonas_aeruginosa	*GCA_001554995.1*	*0*
Pseudomonas_aeruginosa	*GCA_001555015.1*	*0*
Pseudomonas_aeruginosa	*GCA_001555035.1*	*0*
Pseudomonas_aeruginosa	*GCA_001555055.1*	*0*
Pseudomonas_aeruginosa	*GCA_001555075.1*	*0*
Pseudomonas_aeruginosa	*GCA_001555095.1*	*0*
Pseudomonas_aeruginosa	*GCA_001555115.1*	*0*
Pseudomonas_aeruginosa	*GCA_001555135.1*	*0*
Pseudomonas_aeruginosa	*GCA_001555145.1*	*0*
Pseudomonas_aeruginosa	*GCA_001555155.1*	*0*
Pseudomonas_aeruginosa	*GCA_001555165.1*	*0*
Pseudomonas_aeruginosa	*GCA_001555215.1*	*0*
Pseudomonas_aeruginosa	*GCA_001555225.1*	*0*
Pseudomonas_aeruginosa	*GCA_001555235.1*	*0*
Pseudomonas_aeruginosa	*GCA_001555255.1*	*0*
Pseudomonas_aeruginosa	*GCA_001555295.1*	*0*
Pseudomonas_aeruginosa	*GCA_001555315.1*	*0*
Pseudomonas_aeruginosa	*GCA_001555325.1*	*0*
Pseudomonas_aeruginosa	*GCA_001555335.1*	*0*
Pseudomonas_aeruginosa	*GCA_001555375.1*	*0*
Pseudomonas_aeruginosa	*GCA_001555395.1*	*0*
Pseudomonas_aeruginosa	*GCA_001555415.1*	*0*
Pseudomonas_aeruginosa	*GCA_001555425.1*	*0*

Pseudomonas_aeruginosa	GCA_001555455.1	0
Pseudomonas_aeruginosa	GCA_001560865.1	0
Pseudomonas_aeruginosa	GCA_001594325.1	0
Pseudomonas_aeruginosa	GCA_001601585.1	0
Pseudomonas_aeruginosa	GCA_001601595.1	0
Pseudomonas_aeruginosa	GCA_001601665.1	0
Pseudomonas_aeruginosa	GCA_001601745.1	0
Pseudomonas_aeruginosa	GCA_001606045.1	0
Pseudomonas_aeruginosa	GCA_001618925.1	0
Pseudomonas_aeruginosa	GCA_001619935.1	0
Pseudomonas_aeruginosa	GCA_001623945.1	0
Pseudomonas_aeruginosa	GCA_001623955.1	0
Pseudomonas_aeruginosa	GCA_001623985.1	0
Pseudomonas_aeruginosa	GCA_001632245.1	0
Pseudomonas_aeruginosa	GCA_001679685.1	0
Pseudomonas_aeruginosa	GCA_001679905.1	0
Pseudomonas_aeruginosa	GCA_001680745.1	0
Pseudomonas_aeruginosa	GCA_001721745.1	0
Pseudomonas_aeruginosa	GCA_001721765.1	0
Pseudomonas_aeruginosa	GCA_001721785.1	0
Pseudomonas_aeruginosa	GCA_001721805.1	0
Pseudomonas_aeruginosa	GCA_001721825.1	0
Pseudomonas_aeruginosa	GCA_001721845.1	0
Pseudomonas_aeruginosa	GCA_001722005.2	0
Pseudomonas_aeruginosa	GCA_001722025.1	0
Pseudomonas_aeruginosa	GCA_001722045.1	0
Pseudomonas_aeruginosa	GCA_001729505.1	0
Pseudomonas_aeruginosa	GCA_001750205.1	0
Pseudomonas_aeruginosa	GCA_001756355.1	0
Pseudomonas_aeruginosa	GCA_001756405.1	0
Pseudomonas_aeruginosa	GCA_001756425.1	0
Pseudomonas_aeruginosa	GCA_001756435.1	0
Pseudomonas_aeruginosa	GCA_001756445.1	0
Pseudomonas_aeruginosa	GCA_001756485.1	0
Pseudomonas_aeruginosa	GCA_001756495.1	0
Pseudomonas_aeruginosa	GCA_001756515.1	0
Pseudomonas_aeruginosa	GCA_001756525.1	0
Pseudomonas_aeruginosa	GCA_001756585.1	0
Pseudomonas_aeruginosa	GCA_001792855.1	0
Pseudomonas_aeruginosa	GCA_001806505.1	0
Pseudomonas_aeruginosa	GCA_001870265.1	0
Pseudomonas_aeruginosa	GCA_001874465.1	0
Pseudomonas_aeruginosa	GCA_001879525.1	0
Pseudomonas_aeruginosa	GCA_001900195.1	0
Pseudomonas_aeruginosa	GCA_001900225.1	0
Pseudomonas_aeruginosa	GCA_001900265.1	0
Pseudomonas_aeruginosa	GCA_001909445.1	0
Pseudomonas_aeruginosa	GCA_001909455.1	0
Pseudomonas_aeruginosa	GCA_001909485.1	0
Pseudomonas_aeruginosa	GCA_001909495.1	0
Pseudomonas_aeruginosa	GCA_001909585.1	0
Pseudomonas_aeruginosa	GCA_001909605.1	0
Pseudomonas_aeruginosa	GCA_001909615.1	0
Pseudomonas_aeruginosa	GCA_001909645.1	0
Pseudomonas_aeruginosa	GCA_001909655.1	0

Pseudomonas_aeruginosa	*GCA_001909675.1*	*0*	
Pseudomonas_aeruginosa	*GCA_001909775.1*	*0*	
Pseudomonas_aeruginosa	*GCA_001909795.1*	*0*	
Pseudomonas_aeruginosa	*GCA_001909825.1*	*0*	
Pseudomonas_aeruginosa	*GCA_001909945.1*	*0*	
Pseudomonas_aeruginosa	*GCA_001910045.1*	*0*	
Pseudomonas_aeruginosa	*GCA_001910055.1*	*0*	
Pseudomonas_aeruginosa	*GCA_001910105.1*	*0*	
Pseudomonas_aeruginosa	*GCA_001910115.1*	*0*	
Pseudomonas_aeruginosa	*GCA_001910215.1*	*0*	
Pseudomonas_aeruginosa	*GCA_001910265.1*	*0*	
Pseudomonas_aeruginosa	*GCA_001910285.1*	*0*	
Pseudomonas_aeruginosa	*GCA_001910305.1*	*0*	
Pseudomonas_aeruginosa	*GCA_001910345.1*	*0*	
Pseudomonas_aeruginosa	*GCA_001910365.1*	*0*	
Pseudomonas_aeruginosa	*GCA_001910375.1*	*0*	
Pseudomonas_aeruginosa	*GCA_001910425.1*	*0*	
Pseudomonas_aeruginosa	*GCA_001990285.1*	*0*	
Pseudomonas_aeruginosa	*GCA_001990295.1*	*0*	
Pseudomonas_aeruginosa	*GCA_001990325.1*	*0*	
Pseudomonas_aeruginosa	*GCA_001990335.1*	*0*	
Pseudomonas_aeruginosa	*GCA_001990345.1*	*0*	
Pseudomonas_aeruginosa	*GCA_001990375.1*	*0*	
Pseudomonas_aeruginosa	*GCA_001990405.1*	*0*	
Pseudomonas_aeruginosa	*GCA_001990425.1*	*0*	
Pseudomonas_aeruginosa	*GCA_001990445.1*	*0*	
Pseudomonas_aeruginosa	*GCA_001990525.1*	*0*	
Pseudomonas_aeruginosa	*GCA_001997195.1*	*0*	
Pseudomonas_aeruginosa	*GCA_002001225.1*	*0*	
Pseudomonas_aeruginosa	*GCA_002001235.1*	*0*	
Pseudomonas_aeruginosa	*GCA_002001265.1*	*0*	
Pseudomonas_aeruginosa	*GCA_002001275.1*	*0*	
Pseudomonas_aeruginosa	*GCA_002001305.1*	*0*	
Pseudomonas_aeruginosa	*GCA_002001315.1*	*0*	
Pseudomonas_aeruginosa	*GCA_002001385.1*	*0*	
Pseudomonas_aeruginosa	*GCA_002002365.1*	*0*	
Pseudomonas_aeruginosa	*GCA_002002765.1*	*0*	
Pseudomonas_aeruginosa	*GCA_002003595.1*	*0*	
Pseudomonas_aeruginosa	*GCA_002005755.1*	*0*	
Pseudomonas_aeruginosa	*GCA_002005765.1*	*0*	
Pseudomonas_aeruginosa	*GCA_002021595.1*	*0*	
Pseudomonas_aeruginosa	*GCA_002021605.1*	*0*	
Pseudomonas_aeruginosa	*GCA_002021635.1*	*0*	
Pseudomonas_aeruginosa	*GCA_002021645.1*	*0*	
Pseudomonas_aeruginosa	*GCA_002029925.1*	*0*	
Pseudomonas_aeruginosa	*GCA_002075065.1*	*0*	
Pseudomonas_aeruginosa	*GCA_002085605.1*	*0*	
Pseudomonas_aeruginosa	*GCA_002085755.1*	*0*	
Pseudomonas_aeruginosa	*GCA_002088145.1*	*0*	
Pseudomonas_aeruginosa	*GCA_002088215.1*	*0*	
Pseudomonas_aeruginosa	*GCA_002088225.1*	*0*	
Pseudomonas_aeruginosa	*GCA_900070375.1*	*0*	
Pseudomonas_aeruginosa	*GCA_900095805.1*	*0*	
Pseudomonas_aeruginosa	*GCA_900149285.1*	*0*	
Pseudomonas_aeruginosa	*GCA_900168095.1*	*0*	

Pseudomonas_aeruginosa	*GCF_000737795.1*	*0*
Pseudomonas_aeruginosa	*GCF_000751715.1*	*0*
Pseudomonas_aeruginosa	*GCF_000763245.1*	*0*
Pseudomonas_aeruginosa	*GCF_000783275.1*	*0*
Pseudomonas_aeruginosa	*GCF_000789485.1*	*0*
Pseudomonas_aeruginosa	*GCF_000789495.1*	*0*
Pseudomonas_aeruginosa	*GCF_000789525.1*	*0*
Pseudomonas_aeruginosa	*GCF_000789535.1*	*0*
Pseudomonas_aeruginosa	*GCF_000789545.1*	*0*
Pseudomonas_aeruginosa	*GCF_000789555.1*	*0*
Pseudomonas_aeruginosa	*GCF_000789605.1*	*0*
Pseudomonas_aeruginosa	*GCF_000789625.1*	*0*
Pseudomonas_aeruginosa	*GCF_000789635.1*	*0*
Pseudomonas_aeruginosa	*GCF_000789645.1*	*0*
Pseudomonas_aeruginosa	*GCF_000789685.1*	*0*
Pseudomonas_aeruginosa	*GCF_000789705.1*	*0*
Pseudomonas_aeruginosa	*GCF_000789725.1*	*0*
Pseudomonas_aeruginosa	*GCF_000789745.1*	*0*
Pseudomonas_aeruginosa	*GCF_000789755.1*	*0*
Pseudomonas_aeruginosa	*GCF_000789785.1*	*0*
Pseudomonas_aeruginosa	*GCF_000789805.1*	*0*
Pseudomonas_aeruginosa	*GCF_000789815.1*	*0*
Pseudomonas_aeruginosa	*GCF_000789835.1*	*0*
Pseudomonas_aeruginosa	*GCF_000789845.1*	*0*
Pseudomonas_aeruginosa	*GCF_000789885.1*	*0*
Pseudomonas_aeruginosa	*GCF_000789905.1*	*0*
Pseudomonas_aeruginosa	*GCF_000789925.1*	*0*
Pseudomonas_aeruginosa	*GCF_000789935.1*	*0*
Pseudomonas_aeruginosa	*GCF_000789965.1*	*0*
Pseudomonas_aeruginosa	*GCF_000789975.1*	*0*
Pseudomonas_aeruginosa	*GCF_000789995.1*	*0*
Pseudomonas_aeruginosa	*GCF_000790025.1*	*0*
Pseudomonas_aeruginosa	*GCF_000790035.1*	*0*
Pseudomonas_aeruginosa	*GCF_000790065.1*	*0*
Pseudomonas_aeruginosa	*GCF_000790085.1*	*0*
Pseudomonas_aeruginosa	*GCF_000790105.1*	*0*
Pseudomonas_aeruginosa	*GCF_000790115.1*	*0*
Pseudomonas_aeruginosa	*GCF_000790145.1*	*0*
Pseudomonas_aeruginosa	*GCF_000790155.1*	*0*
Pseudomonas_aeruginosa	*GCF_000790185.1*	*0*
Pseudomonas_aeruginosa	*GCF_000790205.1*	*0*
Pseudomonas_aeruginosa	*GCF_000790215.1*	*0*
Pseudomonas_aeruginosa	*GCF_000790245.1*	*0*
Pseudomonas_aeruginosa	*GCF_000790265.1*	*0*
Pseudomonas_aeruginosa	*GCF_000790285.1*	*0*
Pseudomonas_aeruginosa	*GCF_000790305.1*	*0*
Pseudomonas_aeruginosa	*GCF_000790325.1*	*0*
Pseudomonas_aeruginosa	*GCF_000790345.1*	*0*
Pseudomonas_aeruginosa	*GCF_000790355.1*	*0*
Pseudomonas_aeruginosa	*GCF_000790385.1*	*0*
Pseudomonas_aeruginosa	*GCF_000790425.1*	*0*
Pseudomonas_aeruginosa	*GCF_000790445.1*	*0*
Pseudomonas_aeruginosa	*GCF_000790465.1*	*0*
Pseudomonas_aeruginosa	*GCF_000790485.1*	*0*
Pseudomonas_aeruginosa	*GCF_000790505.1*	*0*

Pseudomonas_aeruginosa	GCF_000790525.1	0	
Pseudomonas_aeruginosa	GCF_000790545.1	0	
Pseudomonas_aeruginosa	GCF_000790565.1	0	
Pseudomonas_aeruginosa	GCF_000790585.1	0	
Pseudomonas_aeruginosa	GCF_000790605.1	0	
Pseudomonas_aeruginosa	GCF_000790625.1	0	
Pseudomonas_aeruginosa	GCF_000790645.1	0	
Pseudomonas_aeruginosa	GCF_000790655.1	0	
Pseudomonas_aeruginosa	GCF_000790685.1	0	
Pseudomonas_aeruginosa	GCF_000790705.1	0	
Pseudomonas_aeruginosa	GCF_000790725.1	0	
Pseudomonas_aeruginosa	GCF_000790735.1	0	
Pseudomonas_aeruginosa	GCF_000790765.1	0	
Pseudomonas_aeruginosa	GCF_000790785.1	0	
Pseudomonas_aeruginosa	GCF_000790805.1	0	
Pseudomonas_aeruginosa	GCF_000790825.1	0	
Pseudomonas_aeruginosa	GCF_000790835.1	0	
Pseudomonas_aeruginosa	GCF_000790865.1	0	
Pseudomonas_aeruginosa	GCF_000790885.1	0	
Pseudomonas_aeruginosa	GCF_000790905.1	0	
Pseudomonas_aeruginosa	GCF_000790915.1	0	
Pseudomonas_aeruginosa	GCF_000790935.1	0	
Pseudomonas_aeruginosa	GCF_000790965.1	0	
Pseudomonas_aeruginosa	GCF_000790985.1	0	
Pseudomonas_aeruginosa	GCF_000791005.1	0	
Pseudomonas_aeruginosa	GCF_000791025.1	0	
Pseudomonas_aeruginosa	GCF_000791035.1	0	
Pseudomonas_aeruginosa	GCF_000791065.1	0	
Pseudomonas_aeruginosa	GCF_000791085.1	0	
Pseudomonas_aeruginosa	GCF_000791105.1	0	
Pseudomonas_aeruginosa	GCF_000791125.1	0	
Pseudomonas_aeruginosa	GCF_000791145.1	0	
Pseudomonas_aeruginosa	GCF_000791155.1	0	
Pseudomonas_aeruginosa	GCF_000791185.1	0	
Pseudomonas_aeruginosa	GCF_000791205.1	0	
Pseudomonas_aeruginosa	GCF_000791225.1	0	
Pseudomonas_aeruginosa	GCF_000791235.1	0	
Pseudomonas_aeruginosa	GCF_000791265.1	0	
Pseudomonas_aeruginosa	GCF_000791275.1	0	
Pseudomonas_aeruginosa	GCF_000791305.1	0	
Pseudomonas_aeruginosa	GCF_000791325.1	0	
Pseudomonas_aeruginosa	GCF_000791345.1	0	
Pseudomonas_aeruginosa	GCF_000791365.1	0	
Pseudomonas_aeruginosa	GCF_000791385.1	0	
Pseudomonas_aeruginosa	GCF_000791405.1	0	
Pseudomonas_aeruginosa	GCF_000791425.1	0	
Pseudomonas_aeruginosa	GCF_000791445.1	0	
Pseudomonas_aeruginosa	GCF_000791465.1	0	
Pseudomonas_aeruginosa	GCF_000791485.1	0	
Pseudomonas_aeruginosa	GCF_000791495.1	0	
Pseudomonas_aeruginosa	GCF_000791525.1	0	
Pseudomonas_aeruginosa	GCF_000791545.1	0	
Pseudomonas_aeruginosa	GCF_000791565.1	0	
Pseudomonas_aeruginosa	GCF_000791585.1	0	
Pseudomonas_aeruginosa	GCF_000791605.1	0	

Pseudomonas_aeruginosa	GCF_000791625.1	0
Pseudomonas_aeruginosa	GCF_000791635.1	0
Pseudomonas_aeruginosa	GCF_000791665.1	0
Pseudomonas_aeruginosa	GCF_000791685.1	0
Pseudomonas_aeruginosa	GCF_000791705.1	0
Pseudomonas_aeruginosa	GCF_000791725.1	0
Pseudomonas_aeruginosa	GCF_000791735.1	0
Pseudomonas_aeruginosa	GCF_000791765.1	0
Pseudomonas_aeruginosa	GCF_000791785.1	0
Pseudomonas_aeruginosa	GCF_000791805.1	0
Pseudomonas_aeruginosa	GCF_000791825.1	0
Pseudomonas_aeruginosa	GCF_000791835.1	0
Pseudomonas_aeruginosa	GCF_000791855.1	0
Pseudomonas_aeruginosa	GCF_000791885.1	0
Pseudomonas_aeruginosa	GCF_000791905.1	0
Pseudomonas_aeruginosa	GCF_000791915.1	0
Pseudomonas_aeruginosa	GCF_000791945.1	0
Pseudomonas_aeruginosa	GCF_000791965.1	0
Pseudomonas_aeruginosa	GCF_000791985.1	0
Pseudomonas_aeruginosa	GCF_000792005.1	0
Pseudomonas_aeruginosa	GCF_000792025.1	0
Pseudomonas_aeruginosa	GCF_000792045.1	0
Pseudomonas_aeruginosa	GCF_000792065.1	0
Pseudomonas_aeruginosa	GCF_000792085.1	0
Pseudomonas_aeruginosa	GCF_000792095.1	0
Pseudomonas_aeruginosa	GCF_000792115.1	0
Pseudomonas_aeruginosa	GCF_000792145.1	0
Pseudomonas_aeruginosa	GCF_000792155.1	0
Pseudomonas_aeruginosa	GCF_000792165.1	0
Pseudomonas_aeruginosa	GCF_000792205.1	0
Pseudomonas_aeruginosa	GCF_000792225.1	0
Pseudomonas_aeruginosa	GCF_000792245.1	0
Pseudomonas_aeruginosa	GCF_000792265.1	0
Pseudomonas_aeruginosa	GCF_000792285.1	0
Pseudomonas_aeruginosa	GCF_000792305.1	0
Pseudomonas_aeruginosa	GCF_000792325.1	0
Pseudomonas_aeruginosa	GCF_000792345.1	0
Pseudomonas_aeruginosa	GCF_000792365.1	0
Pseudomonas_aeruginosa	GCF_000792385.1	0
Pseudomonas_aeruginosa	GCF_000792395.1	0
Pseudomonas_aeruginosa	GCF_000792425.1	0
Pseudomonas_aeruginosa	GCF_000792445.1	0
Pseudomonas_aeruginosa	GCF_000792465.1	0
Pseudomonas_aeruginosa	GCF_000792475.1	0
Pseudomonas_aeruginosa	GCF_000792505.1	0
Pseudomonas_aeruginosa	GCF_000792515.1	0
Pseudomonas_aeruginosa	GCF_000792545.1	0
Pseudomonas_aeruginosa	GCF_000792565.1	0
Pseudomonas_aeruginosa	GCF_000792575.1	0
Pseudomonas_aeruginosa	GCF_000792605.1	0
Pseudomonas_aeruginosa	GCF_000792625.1	0
Pseudomonas_aeruginosa	GCF_000792635.1	0
Pseudomonas_aeruginosa	GCF_000792665.1	0
Pseudomonas_aeruginosa	GCF_000792675.1	0
Pseudomonas_aeruginosa	GCF_000792705.1	0

Pseudomonas_aeruginosa	*GCF_000792725.1*	*0*
Pseudomonas_aeruginosa	*GCF_000792745.1*	*0*
Pseudomonas_aeruginosa	*GCF_000792765.1*	*0*
Pseudomonas_aeruginosa	*GCF_000792785.1*	*0*
Pseudomonas_aeruginosa	*GCF_000792805.1*	*0*
Pseudomonas_aeruginosa	*GCF_000792825.1*	*0*
Pseudomonas_aeruginosa	*GCF_000792845.1*	*0*
Pseudomonas_aeruginosa	*GCF_000792855.1*	*0*
Pseudomonas_aeruginosa	*GCF_000792885.1*	*0*
Pseudomonas_aeruginosa	*GCF_000792895.1*	*0*
Pseudomonas_aeruginosa	*GCF_000792925.1*	*0*
Pseudomonas_aeruginosa	*GCF_000792945.1*	*0*
Pseudomonas_aeruginosa	*GCF_000792965.1*	*0*
Pseudomonas_aeruginosa	*GCF_000792975.1*	*0*
Pseudomonas_aeruginosa	*GCF_000793005.1*	*0*
Pseudomonas_aeruginosa	*GCF_000793025.1*	*0*
Pseudomonas_aeruginosa	*GCF_000793045.1*	*0*
Pseudomonas_aeruginosa	*GCF_000793055.1*	*0*
Pseudomonas_aeruginosa	*GCF_000793085.1*	*0*
Pseudomonas_aeruginosa	*GCF_000793105.1*	*0*
Pseudomonas_aeruginosa	*GCF_000793125.1*	*0*
Pseudomonas_aeruginosa	*GCF_000793135.1*	*0*
Pseudomonas_aeruginosa	*GCF_000793165.1*	*0*
Pseudomonas_aeruginosa	*GCF_000793185.1*	*0*
Pseudomonas_aeruginosa	*GCF_000793205.1*	*0*
Pseudomonas_aeruginosa	*GCF_000793225.1*	*0*
Pseudomonas_aeruginosa	*GCF_000793245.1*	*0*
Pseudomonas_aeruginosa	*GCF_000793255.1*	*0*
Pseudomonas_aeruginosa	*GCF_000793285.1*	*0*
Pseudomonas_aeruginosa	*GCF_000793295.1*	*0*
Pseudomonas_aeruginosa	*GCF_000793315.1*	*0*
Pseudomonas_aeruginosa	*GCF_000793345.1*	*0*
Pseudomonas_aeruginosa	*GCF_000793365.1*	*0*
Pseudomonas_aeruginosa	*GCF_000793385.1*	*0*
Pseudomonas_aeruginosa	*GCF_000793395.1*	*0*
Pseudomonas_aeruginosa	*GCF_000793415.1*	*0*
Pseudomonas_aeruginosa	*GCF_000793445.1*	*0*
Pseudomonas_aeruginosa	*GCF_000793455.1*	*0*
Pseudomonas_aeruginosa	*GCF_000793485.1*	*0*
Pseudomonas_aeruginosa	*GCF_000793505.1*	*0*
Pseudomonas_aeruginosa	*GCF_000793585.1*	*0*
Pseudomonas_aeruginosa	*GCF_000793595.1*	*0*
Pseudomonas_aeruginosa	*GCF_000793615.1*	*0*
Pseudomonas_aeruginosa	*GCF_000793645.1*	*0*
Pseudomonas_aeruginosa	*GCF_000793665.1*	*0*
Pseudomonas_aeruginosa	*GCF_000793685.1*	*0*
Pseudomonas_aeruginosa	*GCF_000793705.1*	*0*
Pseudomonas_aeruginosa	*GCF_000793725.1*	*0*
Pseudomonas_aeruginosa	*GCF_000793745.1*	*0*
Pseudomonas_aeruginosa	*GCF_000793765.1*	*0*
Pseudomonas_aeruginosa	*GCF_000793785.1*	*0*
Pseudomonas_aeruginosa	*GCF_000793805.1*	*0*
Pseudomonas_aeruginosa	*GCF_000793825.1*	*0*
Pseudomonas_aeruginosa	*GCF_000793845.1*	*0*
Pseudomonas_aeruginosa	*GCF_000793865.1*	*0*

Pseudomonas_aeruginosa	*GCF_000793885.1*	*0*	
Pseudomonas_aeruginosa	*GCF_000793905.1*	*0*	
Pseudomonas_aeruginosa	*GCF_000793915.1*	*0*	
Pseudomonas_aeruginosa	*GCF_000793945.1*	*0*	
Pseudomonas_aeruginosa	*GCF_000793955.1*	*0*	
Pseudomonas_aeruginosa	*GCF_000793985.1*	*0*	
Pseudomonas_aeruginosa	*GCF_000794005.1*	*0*	
Pseudomonas_aeruginosa	*GCF_000794025.1*	*0*	
Pseudomonas_aeruginosa	*GCF_000794045.1*	*0*	
Pseudomonas_aeruginosa	*GCF_000794055.1*	*0*	
Pseudomonas_aeruginosa	*GCF_000794085.1*	*0*	
Pseudomonas_aeruginosa	*GCF_000794095.1*	*0*	
Pseudomonas_aeruginosa	*GCF_000794165.1*	*0*	
Pseudomonas_aeruginosa	*GCF_000794175.1*	*0*	
Pseudomonas_aeruginosa	*GCF_000794185.1*	*0*	
Pseudomonas_aeruginosa	*GCF_000794195.1*	*0*	
Pseudomonas_aeruginosa	*GCF_000794245.1*	*0*	
Pseudomonas_aeruginosa	*GCF_000794265.1*	*0*	
Pseudomonas_aeruginosa	*GCF_000794285.1*	*0*	
Pseudomonas_aeruginosa	*GCF_000794305.1*	*0*	
Pseudomonas_aeruginosa	*GCF_000794325.1*	*0*	
Pseudomonas_aeruginosa	*GCF_000794335.1*	*0*	
Pseudomonas_aeruginosa	*GCF_000794365.1*	*0*	
Pseudomonas_aeruginosa	*GCF_000794405.1*	*0*	
Pseudomonas_aeruginosa	*GCF_000794425.1*	*0*	
Pseudomonas_aeruginosa	*GCF_000794445.1*	*0*	
Pseudomonas_aeruginosa	*GCF_000794455.1*	*0*	
Pseudomonas_aeruginosa	*GCF_000794485.1*	*0*	
Pseudomonas_aeruginosa	*GCF_000794505.1*	*0*	
Pseudomonas_aeruginosa	*GCF_000794515.1*	*0*	
Pseudomonas_aeruginosa	*GCF_000794545.1*	*0*	
Pseudomonas_aeruginosa	*GCF_000794555.1*	*0*	
Pseudomonas_aeruginosa	*GCF_000794585.1*	*0*	
Pseudomonas_aeruginosa	*GCF_000794605.1*	*0*	
Pseudomonas_aeruginosa	*GCF_000794625.1*	*0*	
Pseudomonas_aeruginosa	*GCF_000794645.1*	*0*	
Pseudomonas_aeruginosa	*GCF_000794665.1*	*0*	
Pseudomonas_aeruginosa	*GCF_000794675.1*	*0*	
Pseudomonas_aeruginosa	*GCF_000794725.1*	*0*	
Pseudomonas_aeruginosa	*GCF_000794735.1*	*0*	
Pseudomonas_aeruginosa	*GCF_000794745.1*	*0*	
Pseudomonas_aeruginosa	*GCF_000794785.1*	*0*	
Pseudomonas_aeruginosa	*GCF_000794805.1*	*0*	
Pseudomonas_aeruginosa	*GCF_000794825.1*	*0*	
Pseudomonas_aeruginosa	*GCF_000794845.1*	*0*	
Pseudomonas_aeruginosa	*GCF_000794865.1*	*0*	
Pseudomonas_aeruginosa	*GCF_000794885.1*	*0*	
Pseudomonas_aeruginosa	*GCF_000794905.1*	*0*	
Pseudomonas_aeruginosa	*GCF_000794925.1*	*0*	
Pseudomonas_aeruginosa	*GCF_000794945.1*	*0*	
Pseudomonas_aeruginosa	*GCF_000794965.1*	*0*	
Pseudomonas_aeruginosa	*GCF_000794985.1*	*0*	
Pseudomonas_aeruginosa	*GCF_000794995.1*	*0*	
Pseudomonas_aeruginosa	*GCF_000795025.1*	*0*	
Pseudomonas_aeruginosa	*GCF_000795045.1*	*0*	

Pseudomonas_aeruginosa	GCF_000795065.1	0
Pseudomonas_aeruginosa	GCF_000795085.1	0
Pseudomonas_aeruginosa	GCF_000795105.1	0
Pseudomonas_aeruginosa	GCF_000795115.1	0
Pseudomonas_aeruginosa	GCF_000795145.1	0
Pseudomonas_aeruginosa	GCF_000795165.1	0
Pseudomonas_aeruginosa	GCF_000795185.1	0
Pseudomonas_aeruginosa	GCF_000795205.1	0
Pseudomonas_aeruginosa	GCF_000795225.1	0
Pseudomonas_aeruginosa	GCF_000795235.1	0
Pseudomonas_aeruginosa	GCF_000795265.1	0
Pseudomonas_aeruginosa	GCF_000795285.1	0
Pseudomonas_aeruginosa	GCF_000795305.1	0
Pseudomonas_aeruginosa	GCF_000795325.1	0
Pseudomonas_aeruginosa	GCF_000795345.1	0
Pseudomonas_aeruginosa	GCF_000795365.1	0
Pseudomonas_aeruginosa	GCF_000795385.1	0
Pseudomonas_aeruginosa	GCF_000795405.1	0
Pseudomonas_aeruginosa	GCF_000795425.1	0
Pseudomonas_aeruginosa	GCF_000795465.1	0
Pseudomonas_aeruginosa	GCF_000795485.1	0
Pseudomonas_aeruginosa	GCF_000795505.1	0
Pseudomonas_aeruginosa	GCF_000795525.1	0
Pseudomonas_aeruginosa	GCF_000795535.1	0
Pseudomonas_aeruginosa	GCF_000795565.1	0
Pseudomonas_aeruginosa	GCF_000795585.1	0
Pseudomonas_aeruginosa	GCF_000795605.1	0
Pseudomonas_aeruginosa	GCF_000795625.1	0
Pseudomonas_aeruginosa	GCF_000795635.1	0
Pseudomonas_aeruginosa	GCF_000795665.1	0
Pseudomonas_aeruginosa	GCF_000795685.1	0
Pseudomonas_aeruginosa	GCF_000795705.1	0
Pseudomonas_aeruginosa	GCF_000795725.1	0
Pseudomonas_aeruginosa	GCF_000795735.1	0
Pseudomonas_aeruginosa	GCF_000795765.1	0
Pseudomonas_aeruginosa	GCF_000795785.1	0
Pseudomonas_aeruginosa	GCF_000795805.1	0
Pseudomonas_aeruginosa	GCF_000795815.1	0
Pseudomonas_aeruginosa	GCF_000795845.1	0
Pseudomonas_aeruginosa	GCF_000795865.1	0
Pseudomonas_aeruginosa	GCF_000795875.1	0
Pseudomonas_aeruginosa	GCF_000795905.1	0
Pseudomonas_aeruginosa	GCF_000795925.1	0
Pseudomonas_aeruginosa	GCF_000795945.1	0
Pseudomonas_aeruginosa	GCF_000795955.1	0
Pseudomonas_aeruginosa	GCF_000795985.1	0
Pseudomonas_aeruginosa	GCF_000796005.1	0
Pseudomonas_aeruginosa	GCF_000796025.1	0
Pseudomonas_aeruginosa	GCF_000796045.1	0
Pseudomonas_aeruginosa	GCF_000796065.1	0
Pseudomonas_aeruginosa	GCF_000796085.1	0
Pseudomonas_aeruginosa	GCF_000796095.1	0
Pseudomonas_aeruginosa	GCF_000796125.1	0
Pseudomonas_aeruginosa	GCF_000796145.1	0
Pseudomonas_aeruginosa	GCF_000796165.1	0

Pseudomonas_aeruginosa	GCF_000796175.1	0
Pseudomonas_aeruginosa	GCF_000796205.1	0
Pseudomonas_aeruginosa	GCF_000796225.1	0
Pseudomonas_aeruginosa	GCF_000796245.1	0
Pseudomonas_aeruginosa	GCF_000796255.1	0
Pseudomonas_aeruginosa	GCF_000796285.1	0
Pseudomonas_aeruginosa	GCF_000796295.1	0
Pseudomonas_aeruginosa	GCF_000796325.1	0
Pseudomonas_aeruginosa	GCF_000796345.1	0
Pseudomonas_aeruginosa	GCF_000796365.1	0
Pseudomonas_aeruginosa	GCF_000796385.1	0
Pseudomonas_aeruginosa	GCF_000796405.1	0
Pseudomonas_aeruginosa	GCF_000796425.1	0
Pseudomonas_aeruginosa	GCF_000796445.1	0
Pseudomonas_aeruginosa	GCF_000796465.1	0
Pseudomonas_aeruginosa	GCF_000796475.1	0
Pseudomonas_aeruginosa	GCF_000796505.1	0
Pseudomonas_aeruginosa	GCF_000796525.1	0
Pseudomonas_aeruginosa	GCF_000796545.1	0
Pseudomonas_aeruginosa	GCF_000796555.1	0
Pseudomonas_aeruginosa	GCF_000796585.1	0
Pseudomonas_aeruginosa	GCF_000796605.1	0
Pseudomonas_aeruginosa	GCF_000796615.1	0
Pseudomonas_aeruginosa	GCF_000796645.1	0
Pseudomonas_aeruginosa	GCF_000796655.1	0
Pseudomonas_aeruginosa	GCF_000796685.1	0
Pseudomonas_aeruginosa	GCF_000796705.1	0
Pseudomonas_aeruginosa	GCF_000796725.1	0
Pseudomonas_aeruginosa	GCF_000796745.1	0
Pseudomonas_aeruginosa	GCF_000796765.1	0
Pseudomonas_aeruginosa	GCF_000796785.1	0
Pseudomonas_aeruginosa	GCF_000796795.1	0
Pseudomonas_aeruginosa	GCF_000796825.1	0
Pseudomonas_aeruginosa	GCF_000796845.1	0
Pseudomonas_aeruginosa	GCF_000796865.1	0
Pseudomonas_aeruginosa	GCF_000796875.1	0
Pseudomonas_aeruginosa	GCF_000796905.1	0
Pseudomonas_aeruginosa	GCF_000796925.1	0
Pseudomonas_aeruginosa	GCF_000796945.1	0
Pseudomonas_aeruginosa	GCF_000796955.1	0
Pseudomonas_aeruginosa	GCF_000796985.1	0
Pseudomonas_aeruginosa	GCF_000797005.1	0
Pseudomonas_aeruginosa	GCF_000797025.1	0
Pseudomonas_aeruginosa	GCF_000797045.1	0
Pseudomonas_aeruginosa	GCF_000797055.1	0
Pseudomonas_aeruginosa	GCF_000797085.1	0
Pseudomonas_aeruginosa	GCF_000797105.1	0
Pseudomonas_aeruginosa	GCF_000797125.1	0
Pseudomonas_aeruginosa	GCF_000797165.1	0
Pseudomonas_aeruginosa	GCF_000797185.1	0
Pseudomonas_aeruginosa	GCF_000797205.1	0
Pseudomonas_aeruginosa	GCF_000797225.1	0
Pseudomonas_aeruginosa	GCF_000797245.1	0
Pseudomonas_aeruginosa	GCF_000797265.1	0
Pseudomonas_aeruginosa	GCF_000797285.1	0

Pseudomonas_aeruginosa	GCF_000797305.1	0	
Pseudomonas_aeruginosa	GCF_000797325.1	0	
Pseudomonas_aeruginosa	GCF_000797345.1	0	
Pseudomonas_aeruginosa	GCF_000797355.1	0	
Pseudomonas_aeruginosa	GCF_000797395.1	0	
Pseudomonas_aeruginosa	GCF_000813565.1	0	
Pseudomonas_aeruginosa	GCF_000817165.1	0	
Pseudomonas_aeruginosa	GCF_000817865.1	0	
Pseudomonas_aeruginosa	GCF_000823905.1	0	
Pseudomonas_aeruginosa	GCF_000823925.1	0	
Pseudomonas_aeruginosa	GCF_000823945.1	0	
Pseudomonas_aeruginosa	GCF_000823965.1	0	
Pseudomonas_aeruginosa	GCF_000823985.1	0	
Pseudomonas_aeruginosa	GCF_000824005.1	0	
Pseudomonas_aeruginosa	GCF_000824025.1	0	
Pseudomonas_aeruginosa	GCF_000824045.1	0	
Pseudomonas_aeruginosa	GCF_000824065.1	0	
Pseudomonas_aeruginosa	GCF_000824085.1	0	
Pseudomonas_aeruginosa	GCF_000824105.1	0	
Pseudomonas_aeruginosa	GCF_000824125.1	0	
Pseudomonas_aeruginosa	GCF_000824145.1	0	
Pseudomonas_aeruginosa	GCF_000824165.1	0	
Pseudomonas_aeruginosa	GCF_000824185.1	0	
Pseudomonas_aeruginosa	GCF_000824205.1	0	
Pseudomonas_aeruginosa	GCF_000824225.1	0	
Pseudomonas_aeruginosa	GCF_000824245.1	0	
Pseudomonas_aeruginosa	GCF_000824265.1	0	
Pseudomonas_aeruginosa	GCF_000824285.1	0	
Pseudomonas_aeruginosa	GCF_000824305.1	0	
Pseudomonas_aeruginosa	GCF_000824325.1	0	
Pseudomonas_aeruginosa	GCF_000824345.1	0	
Pseudomonas_aeruginosa	GCF_000824365.1	0	
Pseudomonas_aeruginosa	GCF_000824385.1	0	
Pseudomonas_aeruginosa	GCF_000824405.1	0	
Pseudomonas_aeruginosa	GCF_000824425.1	0	
Pseudomonas_aeruginosa	GCF_000824445.1	0	
Pseudomonas_aeruginosa	GCF_000824465.1	0	
Pseudomonas_aeruginosa	GCF_000824485.1	0	
Pseudomonas_aeruginosa	GCF_000824505.1	0	
Pseudomonas_aeruginosa	GCF_000824525.1	0	
Pseudomonas_aeruginosa	GCF_000824545.1	0	
Pseudomonas_aeruginosa	GCF_000824565.1	0	
Pseudomonas_aeruginosa	GCF_000824585.1	0	
Pseudomonas_aeruginosa	GCF_000824605.1	0	
Pseudomonas_aeruginosa	GCF_000824625.1	0	
Pseudomonas_aeruginosa	GCF_000824645.1	0	
Pseudomonas_aeruginosa	GCF_000824665.1	0	
Pseudomonas_aeruginosa	GCF_000824685.1	0	
Pseudomonas_aeruginosa	GCF_000950725.1	0	
Pseudomonas_aeruginosa	GCF_000968815.1	0	
Pseudomonas_aeruginosa	GCF_000974565.1	0	
Pseudomonas_aeruginosa	GCF_001007185.1	0	
Pseudomonas_aeruginosa	GCF_001007205.1	0	
Pseudomonas_aeruginosa	GCF_001007215.1	0	
Pseudomonas_aeruginosa	GCF_001007265.1	0	

Pseudomonas_aeruginosa	*GCF_001007275.1*	*0*	
Pseudomonas_aeruginosa	*GCF_001007315.1*	*0*	
Pseudomonas_aeruginosa	*GCF_001007345.1*	*0*	
Pseudomonas_aeruginosa	*GCF_001007395.1*	*0*	
Pseudomonas_aeruginosa	*GCF_001007425.1*	*0*	
Pseudomonas_aeruginosa	*GCF_001007475.1*	*0*	
Pseudomonas_aeruginosa	*GCF_001010525.1*	*0*	
Pseudomonas_aeruginosa	*GCF_001010535.1*	*0*	
Pseudomonas_aeruginosa	*GCF_001010545.1*	*0*	
Pseudomonas_aeruginosa	*GCF_001023615.1*	*0*	
Pseudomonas_aeruginosa	*GCF_001023625.1*	*0*	
Pseudomonas_aeruginosa	*GCF_001023635.1*	*0*	
Pseudomonas_aeruginosa	*GCF_001023675.1*	*0*	
Pseudomonas_aeruginosa	*GCF_001023685.1*	*0*	
Pseudomonas_aeruginosa	*GCF_001023705.1*	*0*	
Pseudomonas_aeruginosa	*GCF_001023735.1*	*0*	
Pseudomonas_aeruginosa	*GCF_001023755.1*	*0*	
Pseudomonas_aeruginosa	*GCF_001023765.1*	*0*	
Pseudomonas_aeruginosa	*GCF_001023775.1*	*0*	
Pseudomonas_aeruginosa	*GCF_001023815.1*	*0*	
Pseudomonas_aeruginosa	*GCF_001023835.1*	*0*	
Pseudomonas_aeruginosa	*GCF_001023845.1*	*0*	
Pseudomonas_aeruginosa	*GCF_001023875.1*	*0*	
Pseudomonas_aeruginosa	*GCF_001023895.1*	*0*	
Pseudomonas_aeruginosa	*GCF_001023915.1*	*0*	
Pseudomonas_aeruginosa	*GCF_001023925.1*	*0*	
Pseudomonas_aeruginosa	*GCF_001023955.1*	*0*	
Pseudomonas_aeruginosa	*GCF_001023975.1*	*0*	
Pseudomonas_aeruginosa	*GCF_001023985.1*	*0*	
Pseudomonas_aeruginosa	*GCF_001023995.1*	*0*	
Pseudomonas_aeruginosa	*GCF_001024035.1*	*0*	
Pseudomonas_aeruginosa	*GCF_001024055.1*	*0*	
Pseudomonas_aeruginosa	*GCF_001024065.1*	*0*	
Pseudomonas_aeruginosa	*GCF_001024075.1*	*0*	
Pseudomonas_aeruginosa	*GCF_001024115.1*	*0*	
Pseudomonas_aeruginosa	*GCF_001024125.1*	*0*	
Pseudomonas_aeruginosa	*GCF_001024135.1*	*0*	
Pseudomonas_aeruginosa	*GCF_001024145.1*	*0*	
Pseudomonas_aeruginosa	*GCF_001024195.1*	*0*	
Pseudomonas_aeruginosa	*GCF_001024205.1*	*0*	
Pseudomonas_aeruginosa	*GCF_001024215.1*	*0*	
Pseudomonas_aeruginosa	*GCF_001024235.1*	*0*	
Pseudomonas_aeruginosa	*GCF_001024275.1*	*0*	
Pseudomonas_aeruginosa	*GCF_001024285.1*	*0*	
Pseudomonas_aeruginosa	*GCF_001024305.1*	*0*	
Pseudomonas_aeruginosa	*GCF_001024325.1*	*0*	
Pseudomonas_aeruginosa	*GCF_001024355.1*	*0*	
Pseudomonas_aeruginosa	*GCF_001024365.1*	*0*	
Pseudomonas_aeruginosa	*GCF_001024375.1*	*0*	
Pseudomonas_aeruginosa	*GCF_001024395.1*	*0*	
Pseudomonas_aeruginosa	*GCF_001024435.1*	*0*	
Pseudomonas_aeruginosa	*GCF_001024445.1*	*0*	
Pseudomonas_aeruginosa	*GCF_001024455.1*	*0*	
Pseudomonas_aeruginosa	*GCF_001024495.1*	*0*	
Pseudomonas_aeruginosa	*GCF_001024515.1*	*0*	

Pseudomonas_aeruginosa	*GCF_001024535.1*	*0*
Pseudomonas_aeruginosa	*GCF_001024545.1*	*0*
Pseudomonas_aeruginosa	*GCF_001024565.1*	*0*
Pseudomonas_aeruginosa	*GCF_001024585.1*	*0*
Pseudomonas_aeruginosa	*GCF_001024615.1*	*0*
Pseudomonas_aeruginosa	*GCF_001024635.1*	*0*
Pseudomonas_aeruginosa	*GCF_001024645.1*	*0*
Pseudomonas_aeruginosa	*GCF_001024655.1*	*0*
Pseudomonas_aeruginosa	*GCF_001024695.1*	*0*
Pseudomonas_aeruginosa	*GCF_001024715.1*	*0*
Pseudomonas_aeruginosa	*GCF_001024725.1*	*0*
Pseudomonas_aeruginosa	*GCF_001024755.1*	*0*
Pseudomonas_aeruginosa	*GCF_001024765.1*	*0*
Pseudomonas_aeruginosa	*GCF_001024775.1*	*0*
Pseudomonas_aeruginosa	*GCF_001024805.1*	*0*
Pseudomonas_aeruginosa	*GCF_001024835.1*	*0*
Pseudomonas_aeruginosa	*GCF_001024845.1*	*0*
Pseudomonas_aeruginosa	*GCF_001024855.1*	*0*
Pseudomonas_aeruginosa	*GCF_001024875.1*	*0*
Pseudomonas_aeruginosa	*GCF_001024915.1*	*0*
Pseudomonas_aeruginosa	*GCF_001024925.1*	*0*
Pseudomonas_aeruginosa	*GCF_001024945.1*	*0*
Pseudomonas_aeruginosa	*GCF_001024955.1*	*0*
Pseudomonas_aeruginosa	*GCF_001024995.1*	*0*
Pseudomonas_aeruginosa	*GCF_001034665.1*	*0*
Pseudomonas_aeruginosa	*GCF_001034675.1*	*0*
Pseudomonas_aeruginosa	*GCF_001034685.1*	*0*
Pseudomonas_aeruginosa	*GCF_001034745.1*	*0*
Pseudomonas_aeruginosa	*GCF_001034755.1*	*0*
Pseudomonas_aeruginosa	*GCF_001034765.1*	*0*
Pseudomonas_aeruginosa	*GCF_001034785.1*	*0*
Pseudomonas_aeruginosa	*GCF_001034825.1*	*0*
Pseudomonas_aeruginosa	*GCF_001034835.1*	*0*
Pseudomonas_aeruginosa	*GCF_001034855.1*	*0*
Pseudomonas_aeruginosa	*GCF_001034875.1*	*0*
Pseudomonas_aeruginosa	*GCF_001034905.1*	*0*
Pseudomonas_aeruginosa	*GCF_001034915.1*	*0*
Pseudomonas_aeruginosa	*GCF_001034945.1*	*0*
Pseudomonas_aeruginosa	*GCF_001034955.1*	*0*
Pseudomonas_aeruginosa	*GCF_001034985.1*	*0*
Pseudomonas_aeruginosa	*GCF_001035005.1*	*0*
Pseudomonas_aeruginosa	*GCF_001035025.1*	*0*
Pseudomonas_aeruginosa	*GCF_001035035.1*	*0*
Pseudomonas_aeruginosa	*GCF_001035065.1*	*0*
Pseudomonas_aeruginosa	*GCF_001035075.1*	*0*
Pseudomonas_aeruginosa	*GCF_001035105.1*	*0*
Pseudomonas_aeruginosa	*GCF_001035115.1*	*0*
Pseudomonas_aeruginosa	*GCF_001035145.1*	*0*
Pseudomonas_aeruginosa	*GCF_001035155.1*	*0*
Pseudomonas_aeruginosa	*GCF_001035175.1*	*0*
Pseudomonas_aeruginosa	*GCF_001035205.1*	*0*
Pseudomonas_aeruginosa	*GCF_001035225.1*	*0*
Pseudomonas_aeruginosa	*GCF_001035235.1*	*0*
Pseudomonas_aeruginosa	*GCF_001035245.1*	*0*
Pseudomonas_aeruginosa	*GCF_001035285.1*	*0*

Pseudomonas_aeruginosa	GCF_001035305.1	0	
Pseudomonas_aeruginosa	GCF_001035315.1	0	
Pseudomonas_aeruginosa	GCF_001035325.1	0	
Pseudomonas_aeruginosa	GCF_001035365.1	0	
Pseudomonas_aeruginosa	GCF_001035385.1	0	
Pseudomonas_aeruginosa	GCF_001035395.1	0	
Pseudomonas_aeruginosa	GCF_001035415.1	0	
Pseudomonas_aeruginosa	GCF_001035445.1	0	
Pseudomonas_aeruginosa	GCF_001035465.1	0	
Pseudomonas_aeruginosa	GCF_001035485.1	0	
Pseudomonas_aeruginosa	GCF_001035495.1	0	
Pseudomonas_aeruginosa	GCF_001035525.1	0	
Pseudomonas_aeruginosa	GCF_001035535.1	0	
Pseudomonas_aeruginosa	GCF_001035565.1	0	
Pseudomonas_aeruginosa	GCF_001035575.1	0	
Pseudomonas_aeruginosa	GCF_001035625.1	0	
Pseudomonas_aeruginosa	GCF_001035635.1	0	
Pseudomonas_aeruginosa	GCF_001035645.1	0	
Pseudomonas_aeruginosa	GCF_001035685.1	0	
Pseudomonas_aeruginosa	GCF_001035705.1	0	
Pseudomonas_aeruginosa	GCF_001035715.1	0	
Pseudomonas_aeruginosa	GCF_001035725.1	0	
Pseudomonas_aeruginosa	GCF_001035765.1	0	
Pseudomonas_aeruginosa	GCF_001035785.1	0	
Pseudomonas_aeruginosa	GCF_001035795.1	0	
Pseudomonas_aeruginosa	GCF_001035805.1	0	
Pseudomonas_aeruginosa	GCF_001035845.1	0	
Pseudomonas_aeruginosa	GCF_001035865.1	0	
Pseudomonas_aeruginosa	GCF_001035875.1	0	
Pseudomonas_aeruginosa	GCF_001035885.1	0	
Pseudomonas_aeruginosa	GCF_001035925.1	0	
Pseudomonas_aeruginosa	GCF_001035945.1	0	
Pseudomonas_aeruginosa	GCF_001035955.1	0	
Pseudomonas_aeruginosa	GCF_001035975.1	0	
Pseudomonas_aeruginosa	GCF_001036005.1	0	
Pseudomonas_aeruginosa	GCF_001036025.1	0	
Pseudomonas_aeruginosa	GCF_001036035.1	0	
Pseudomonas_aeruginosa	GCF_001036065.1	0	
Pseudomonas_aeruginosa	GCF_001036105.1	0	
Pseudomonas_aeruginosa	GCF_001036115.1	0	
Pseudomonas_aeruginosa	GCF_001036145.1	0	
Pseudomonas_aeruginosa	GCF_001036155.1	0	
Pseudomonas_aeruginosa	GCF_001036185.1	0	
Pseudomonas_aeruginosa	GCF_001036195.1	0	
Pseudomonas_aeruginosa	GCF_001036225.1	0	
Pseudomonas_aeruginosa	GCF_001036235.1	0	
Pseudomonas_aeruginosa	GCF_001036255.1	0	
Pseudomonas_aeruginosa	GCF_001036265.1	0	
Pseudomonas_aeruginosa	GCF_001036305.1	0	
Pseudomonas_aeruginosa	GCF_001036335.1	0	
Pseudomonas_aeruginosa	GCF_001036355.1	0	
Pseudomonas_aeruginosa	GCF_001036385.1	0	
Pseudomonas_aeruginosa	GCF_001036405.1	0	
Pseudomonas_aeruginosa	GCF_001036415.1	0	
Pseudomonas_aeruginosa	GCF_001036425.1	0	

Pseudomonas_aeruginosa	GCF_001036465.1	0	
Pseudomonas_aeruginosa	GCF_001036485.1	0	
Pseudomonas_aeruginosa	GCF_001036495.1	0	
Pseudomonas_aeruginosa	GCF_001036505.1	0	
Pseudomonas_aeruginosa	GCF_001036545.1	0	
Pseudomonas_aeruginosa	GCF_001036565.1	0	
Pseudomonas_aeruginosa	GCF_001036575.1	0	
Pseudomonas_aeruginosa	GCF_001036585.1	0	
Pseudomonas_aeruginosa	GCF_001036605.1	0	
Pseudomonas_aeruginosa	GCF_001036645.1	0	
Pseudomonas_aeruginosa	GCF_001036655.1	0	
Pseudomonas_aeruginosa	GCF_001036675.1	0	
Pseudomonas_aeruginosa	GCF_001036685.1	0	
Pseudomonas_aeruginosa	GCF_001036725.1	0	
Pseudomonas_aeruginosa	GCF_001036735.1	0	
Pseudomonas_aeruginosa	GCF_001036765.1	0	
Pseudomonas_aeruginosa	GCF_001036775.1	0	
Pseudomonas_aeruginosa	GCF_001036805.1	0	
Pseudomonas_aeruginosa	GCF_001036815.1	0	
Pseudomonas_aeruginosa	GCF_001036825.1	0	
Pseudomonas_aeruginosa	GCF_001036835.1	0	
Pseudomonas_aeruginosa	GCF_001036895.1	0	
Pseudomonas_aeruginosa	GCF_001036905.1	0	
Pseudomonas_aeruginosa	GCF_001036915.1	0	
Pseudomonas_aeruginosa	GCF_001036965.1	0	
Pseudomonas_aeruginosa	GCF_001036975.1	0	
Pseudomonas_aeruginosa	GCF_001036985.1	0	
Pseudomonas_aeruginosa	GCF_001037025.1	0	
Pseudomonas_aeruginosa	GCF_001037045.1	0	
Pseudomonas_aeruginosa	GCF_001037055.1	0	
Pseudomonas_aeruginosa	GCF_001037065.1	0	
Pseudomonas_aeruginosa	GCF_001037125.1	0	
Pseudomonas_aeruginosa	GCF_001037145.1	0	
Pseudomonas_aeruginosa	GCF_001037165.1	0	
Pseudomonas_aeruginosa	GCF_001037185.1	0	
Pseudomonas_aeruginosa	GCF_001037195.1	0	
Pseudomonas_aeruginosa	GCF_001037225.1	0	
Pseudomonas_aeruginosa	GCF_001037235.1	0	
Pseudomonas_aeruginosa	GCF_001037265.1	0	
Pseudomonas_aeruginosa	GCF_001037275.1	0	
Pseudomonas_aeruginosa	GCF_001037305.1	0	
Pseudomonas_aeruginosa	GCF_001037315.1	0	
Pseudomonas_aeruginosa	GCF_001037345.1	0	
Pseudomonas_aeruginosa	GCF_001037355.1	0	
Pseudomonas_aeruginosa	GCF_001037385.1	0	
Pseudomonas_aeruginosa	GCF_001037395.1	0	
Pseudomonas_aeruginosa	GCF_001039325.1	0	
Pseudomonas_aeruginosa	GCF_001060185.1	0	
Pseudomonas_aeruginosa	GCF_001060435.1	0	
Pseudomonas_aeruginosa	GCF_001060735.1	0	
Pseudomonas_aeruginosa	GCF_001061475.1	0	
Pseudomonas_aeruginosa	GCF_001061585.1	0	
Pseudomonas_aeruginosa	GCF_001061875.1	0	
Pseudomonas_aeruginosa	GCF_001061895.1	0	
Pseudomonas_aeruginosa	GCF_001062565.1	0	

Pseudomonas_aeruginosa	GCF_001062835.1	0
Pseudomonas_aeruginosa	GCF_001062995.1	0
Pseudomonas_aeruginosa	GCF_001063005.1	0
Pseudomonas_aeruginosa	GCF_001063225.1	0
Pseudomonas_aeruginosa	GCF_001063795.1	0
Pseudomonas_aeruginosa	GCF_001063865.1	0
Pseudomonas_aeruginosa	GCF_001064305.1	0
Pseudomonas_aeruginosa	GCF_001064905.1	0
Pseudomonas_aeruginosa	GCF_001065035.1	0
Pseudomonas_aeruginosa	GCF_001065075.1	0
Pseudomonas_aeruginosa	GCF_001065395.1	0
Pseudomonas_aeruginosa	GCF_001065515.1	0
Pseudomonas_aeruginosa	GCF_001065755.1	0
Pseudomonas_aeruginosa	GCF_001065925.1	0
Pseudomonas_aeruginosa	GCF_001066075.1	0
Pseudomonas_aeruginosa	GCF_001066185.1	0
Pseudomonas_aeruginosa	GCF_001066625.1	0
Pseudomonas_aeruginosa	GCF_001066895.1	0
Pseudomonas_aeruginosa	GCF_001067065.1	0
Pseudomonas_aeruginosa	GCF_001067105.1	0
Pseudomonas_aeruginosa	GCF_001067395.1	0
Pseudomonas_aeruginosa	GCF_001067725.1	0
Pseudomonas_aeruginosa	GCF_001067795.1	0
Pseudomonas_aeruginosa	GCF_001067875.1	0
Pseudomonas_aeruginosa	GCF_001067935.1	0
Pseudomonas_aeruginosa	GCF_001067965.1	0
Pseudomonas_aeruginosa	GCF_001068115.1	0
Pseudomonas_aeruginosa	GCF_001068165.1	0
Pseudomonas_aeruginosa	GCF_001068295.1	0
Pseudomonas_aeruginosa	GCF_001068375.1	0
Pseudomonas_aeruginosa	GCF_001068395.1	0
Pseudomonas_aeruginosa	GCF_001069315.1	0
Pseudomonas_aeruginosa	GCF_001076225.1	0
Pseudomonas_aeruginosa	GCF_001076545.1	0
Pseudomonas_aeruginosa	GCF_001076855.1	0
Pseudomonas_aeruginosa	GCF_001077135.1	0
Pseudomonas_aeruginosa	GCF_001086625.1	0
Pseudomonas_aeruginosa	GCF_001086635.1	0
Pseudomonas_aeruginosa	GCF_001086645.1	0
Pseudomonas_aeruginosa	GCF_001086655.1	0
Pseudomonas_aeruginosa	GCF_001086725.1	0
Pseudomonas_aeruginosa	GCF_001086735.1	0
Pseudomonas_aeruginosa	GCF_001086745.1	0
Pseudomonas_aeruginosa	GCF_001086755.1	0
Pseudomonas_aeruginosa	GCF_001086805.1	0
Pseudomonas_aeruginosa	GCF_001280745.1	0
Pseudomonas_aeruginosa	GCF_001280755.1	0
Pseudomonas_aeruginosa	GCF_001280765.1	0
Pseudomonas_aeruginosa	GCF_001373595.1	0
Pseudomonas_aeruginosa	GCF_001373615.1	0
Pseudomonas_aeruginosa	GCF_001373635.1	0
Pseudomonas_aeruginosa	GCF_001373655.1	0
Pseudomonas_aeruginosa	GCF_001373675.1	0
Pseudomonas_aeruginosa	GCF_001373695.1	0
Pseudomonas_aeruginosa	GCF_001373715.1	0

Pseudomonas_aeruginosa	GCF_001373735.1	0	
Pseudomonas_aeruginosa	GCF_001373755.1	0	
Pseudomonas_aeruginosa	GCF_001373775.1	0	
Pseudomonas_aeruginosa	GCF_001373795.1	0	
Pseudomonas_aeruginosa	GCF_001373815.1	0	
Pseudomonas_aeruginosa	GCF_001373835.1	0	
Pseudomonas_aeruginosa	GCF_001373855.1	0	
Pseudomonas_aeruginosa	GCF_001373875.1	0	
Pseudomonas_aeruginosa	GCF_001373895.1	0	
Pseudomonas_aeruginosa	GCF_001373915.1	0	
Pseudomonas_aeruginosa	GCF_001373935.1	0	
Pseudomonas_aeruginosa	GCF_001373955.1	0	
Pseudomonas_aeruginosa	GCF_001373975.1	0	
Pseudomonas_aeruginosa	GCF_001373995.1	0	
Pseudomonas_aeruginosa	GCF_001374015.1	0	
Pseudomonas_aeruginosa	GCF_001374035.1	0	
Pseudomonas_aeruginosa	GCF_001374055.1	0	
Pseudomonas_aeruginosa	GCF_001374075.1	0	
Pseudomonas_aeruginosa	GCF_001374095.1	0	
Pseudomonas_aeruginosa	GCF_001374115.1	0	
Pseudomonas_aeruginosa	GCF_001374135.1	0	
Pseudomonas_aeruginosa	GCF_001374155.1	0	
Pseudomonas_aeruginosa	GCF_001374175.1	0	
Pseudomonas_aeruginosa	GCF_001374195.1	0	
Pseudomonas_aeruginosa	GCF_001374215.1	0	
Pseudomonas_aeruginosa	GCF_001374235.1	0	
Pseudomonas_aeruginosa	GCF_001374255.1	0	
Pseudomonas_aeruginosa	GCF_001374275.1	0	
Pseudomonas_aeruginosa	GCF_001374295.1	0	
Pseudomonas_aeruginosa	GCF_001374315.1	0	
Pseudomonas_aeruginosa	GCF_001374335.1	0	
Pseudomonas_aeruginosa	GCF_001374355.1	0	
Pseudomonas_aeruginosa	GCF_001374375.1	0	
Pseudomonas_aeruginosa	GCF_001374395.1	0	
Pseudomonas_aeruginosa	GCF_001374415.1	0	
Pseudomonas_aeruginosa	GCF_001374435.1	0	
Pseudomonas_aeruginosa	GCF_001374455.1	0	
Pseudomonas_aeruginosa	GCF_001374475.1	0	
Pseudomonas_aeruginosa	GCF_001374495.1	0	
Pseudomonas_aeruginosa	GCF_001374515.1	0	
Pseudomonas_aeruginosa	GCF_001374535.1	0	
Pseudomonas_aeruginosa	GCF_001374555.1	0	
Pseudomonas_aeruginosa	GCF_001374575.1	0	
Pseudomonas_aeruginosa	GCF_001374595.1	0	
Pseudomonas_aeruginosa	GCF_001374615.1	0	
Pseudomonas_aeruginosa	GCF_001374635.1	0	
Pseudomonas_aeruginosa	GCF_001374655.1	0	
Pseudomonas_aeruginosa	GCF_001374675.1	0	
Pseudomonas_aeruginosa	GCF_001374695.1	0	
Pseudomonas_aeruginosa	GCF_001374715.1	0	
Pseudomonas_aeruginosa	GCF_001374735.1	0	
Pseudomonas_aeruginosa	GCF_001374755.1	0	
Pseudomonas_aeruginosa	GCF_001374775.1	0	
Pseudomonas_aeruginosa	GCF_001374795.1	0	
Pseudomonas_aeruginosa	GCF_001374815.1	0	

Pseudomonas_aeruginosa	GCF_001374835.1	0	
Pseudomonas_aeruginosa	GCF_001374855.1	0	
Pseudomonas_aeruginosa	GCF_001374875.1	0	
Pseudomonas_aeruginosa	GCF_001374895.1	0	
Pseudomonas_aeruginosa	GCF_001374915.1	0	
Pseudomonas_aeruginosa	GCF_001374935.1	0	
Pseudomonas_aeruginosa	GCF_001374955.1	0	
Pseudomonas_aeruginosa	GCF_001374975.1	0	
Pseudomonas_aeruginosa	GCF_001374995.1	0	
Pseudomonas_aeruginosa	GCF_001375015.1	0	
Pseudomonas_aeruginosa	GCF_001375035.1	0	
Pseudomonas_aeruginosa	GCF_001375055.1	0	
Pseudomonas_aeruginosa	GCF_001375075.1	0	
Pseudomonas_aeruginosa	GCF_001375095.1	0	
Pseudomonas_aeruginosa	GCF_001375115.1	0	
Pseudomonas_aeruginosa	GCF_001375135.1	0	
Pseudomonas_aeruginosa	GCF_001375155.1	0	
Pseudomonas_aeruginosa	GCF_001375175.1	0	
Pseudomonas_aeruginosa	GCF_001375195.1	0	
Pseudomonas_aeruginosa	GCF_001375215.1	0	
Pseudomonas_aeruginosa	GCF_001375235.1	0	
Pseudomonas_aeruginosa	GCF_001375255.1	0	
Pseudomonas_aeruginosa	GCF_001375275.1	0	
Pseudomonas_aeruginosa	GCF_001375295.1	0	
Pseudomonas_aeruginosa	GCF_001375315.1	0	
Pseudomonas_aeruginosa	GCF_001375335.1	0	
Pseudomonas_aeruginosa	GCF_001375355.1	0	
Pseudomonas_aeruginosa	GCF_001375375.1	0	
Pseudomonas_aeruginosa	GCF_001375395.1	0	
Pseudomonas_aeruginosa	GCF_001375415.1	0	
Pseudomonas_aeruginosa	GCF_001375435.1	0	
Pseudomonas_aeruginosa	GCF_001375455.1	0	
Pseudomonas_aeruginosa	GCF_001375475.1	0	
Pseudomonas_aeruginosa	GCF_001482875.1	0	
Pseudomonas_aeruginosa	GCF_001765435.1	0	
Pseudomonas_aeruginosa	GCF_001765455.1	0	
Pseudomonas_aeruginosa	GCF_001765465.1	0	
Pseudomonas_aeruginosa	GCF_001765475.1	0	
Pseudomonas_aeruginosa	GCF_001765535.1	0	
Pseudomonas_aeruginosa	GCF_001765545.1	0	
Pseudomonas_aeruginosa	GCF_001765575.1	0	
Pseudomonas_aeruginosa	GCF_001765585.1	0	
Pseudomonas_aeruginosa	GCF_001765595.1	0	
Pseudomonas_aeruginosa	GCF_001765635.1	0	
Pseudomonas_aeruginosa	GCF_001765645.1	0	
Pseudomonas_aeruginosa	GCF_001765655.1	0	
Pseudomonas_aeruginosa	GCF_001765695.1	0	
Pseudomonas_aeruginosa	GCF_001765715.1	0	
Pseudomonas_aeruginosa	GCF_001765725.1	0	
Pseudomonas_aeruginosa	GCF_001765755.1	0	
Pseudomonas_aeruginosa	GCF_001765775.1	0	
Pseudomonas_aeruginosa	GCF_001765795.1	0	
Pseudomonas_aeruginosa	GCF_001765805.1	0	
Pseudomonas_aeruginosa	GCF_001765835.1	0	
Pseudomonas_aeruginosa	GCF_001765845.1	0	

Pseudomonas_aeruginosa	GCF_001765855.1	0
Pseudomonas_aeruginosa	GCF_001765865.1	0
Pseudomonas_aeruginosa	GCF_001765915.1	0
Pseudomonas_aeruginosa	GCF_001765925.1	0
Pseudomonas_aeruginosa	GCF_001765955.1	0
Pseudomonas_aeruginosa	GCF_001765975.1	0
Pseudomonas_aeruginosa	GCF_001765985.1	0
Pseudomonas_aeruginosa	GCF_001765995.1	0
Pseudomonas_aeruginosa	GCF_001766035.1	0
Pseudomonas_aeruginosa	GCF_001766045.1	0
Pseudomonas_aeruginosa	GCF_001766075.1	0
Pseudomonas_aeruginosa	GCF_001766085.1	0
Pseudomonas_aeruginosa	GCF_001766115.1	0
Pseudomonas_aeruginosa	GCF_001766125.1	0
Pseudomonas_aeruginosa	GCF_001766135.1	0
Pseudomonas_aeruginosa	GCF_001766145.1	0
Pseudomonas_aeruginosa	GCF_001766195.1	0
Pseudomonas_aeruginosa	GCF_001766205.1	0
Pseudomonas_aeruginosa	GCF_001874795.1	0
Pseudomonas_aeruginosa	GCF_001874805.1	0
Pseudomonas_aeruginosa	GCF_001874955.1	0
Pseudomonas_aeruginosa	GCF_001874975.1	0
Pseudomonas_aeruginosa	GCF_001874995.1	0
Pseudomonas_aeruginosa	GCF_001875165.1	0
Pseudomonas_aeruginosa	GCF_001875185.1	0
Pseudomonas_aeruginosa	GCF_001875205.1	0
Pseudomonas_aeruginosa	GCF_001875215.1	0
Pseudomonas_aeruginosa	GCF_001921005.1	0
Pseudomonas_aeruginosa	GCF_001921015.1	0
Pseudomonas_aeruginosa	GCF_001921025.1	0
Pseudomonas_aeruginosa	GCF_001921065.1	0
Pseudomonas_aeruginosa	GCF_001921085.1	0
Pseudomonas_aeruginosa	GCF_001921095.1	0
Pseudomonas_aeruginosa	GCF_001921105.1	0
Pseudomonas_aeruginosa	GCF_001921125.1	0
Pseudomonas_aeruginosa	GCF_001921165.1	0
Pseudomonas_aeruginosa	GCF_001921175.1	0
Pseudomonas_aeruginosa	GCF_001937975.1	0
Pseudomonas_aeruginosa	GCF_900005845.1	0
Pseudomonas_aeruginosa	GCF_900143755.1	0
Pseudomonas_aeruginosa	GCF_900143765.1	0
Pseudomonas_aeruginosa	GCF_900143775.1	0
Pseudomonas_aeruginosa	GCF_900143795.1	0
Pseudomonas_aeruginosa	GCF_900143805.1	0
Pseudomonas_aeruginosa	GCF_900143815.1	0
Pseudomonas_aeruginosa	GCF_900143825.1	0
Pseudomonas_aeruginosa	GCF_900143835.1	0
Pseudomonas_aeruginosa	GCF_900143845.1	0
Pseudomonas_aeruginosa	GCF_900143855.1	0
Pseudomonas_aeruginosa	GCF_900143865.1	0
Pseudomonas_aeruginosa	GCF_900143875.1	0
Pseudomonas_aeruginosa	GCF_900143885.1	0
Pseudomonas_aeruginosa	GCF_900143895.1	0
Pseudomonas_aeruginosa	GCF_900143905.1	0
Pseudomonas_aeruginosa	GCF_900143915.1	0

Pseudomonas_aeruginosa	*GCF_900143925.1*	*0*
Pseudomonas_aeruginosa	*GCF_900143935.1*	*0*
Pseudomonas_aeruginosa	*GCF_900143945.1*	*0*
Pseudomonas_aeruginosa	*GCF_900143955.1*	*0*
Pseudomonas_aeruginosa	*GCF_900143965.1*	*0*
Pseudomonas_aeruginosa	*GCF_900143975.1*	*0*
Pseudomonas_aeruginosa	*GCF_900143985.1*	*0*
Pseudomonas_aeruginosa	*GCF_900143995.1*	*0*
Pseudomonas_aeruginosa	*GCF_900144005.1*	*0*
Pseudomonas_aeruginosa	*GCF_900144015.1*	*0*
Pseudomonas_aeruginosa	*GCF_900144025.1*	*0*
Pseudomonas_aeruginosa	*GCF_900144035.1*	*0*
Pseudomonas_aeruginosa	*GCF_900144045.1*	*0*
Pseudomonas_aeruginosa	*GCF_900144055.1*	*0*
Pseudomonas_aeruginosa	*GCF_900144065.1*	*0*
Pseudomonas_aeruginosa	*GCF_900144075.1*	*0*
Pseudomonas_aeruginosa	*GCF_900144085.1*	*0*
Pseudomonas_aeruginosa	*GCF_900144095.1*	*0*
Pseudomonas_aeruginosa	*GCF_900144105.1*	*0*
Pseudomonas_aeruginosa	*GCF_900144115.1*	*0*
Pseudomonas_aeruginosa	*GCF_900144135.1*	*0*
Pseudomonas_aeruginosa	*GCF_900144145.1*	*0*
Pseudomonas_aeruginosa	*GCF_900144155.1*	*0*
Pseudomonas_aeruginosa	*GCF_900144165.1*	*0*
Pseudomonas_aeruginosa	*GCF_900144175.1*	*0*
Pseudomonas_aeruginosa	*GCF_900144185.1*	*0*
Pseudomonas_aeruginosa	*GCF_900144195.1*	*0*
Pseudomonas_aeruginosa	*GCF_900144205.1*	*0*
Pseudomonas_aeruginosa	*GCF_900144215.1*	*0*
Pseudomonas_aeruginosa	*GCF_900144225.1*	*0*
Pseudomonas_aeruginosa	*GCF_900144235.1*	*0*
Pseudomonas_aeruginosa	*GCF_900144245.1*	*0*
Pseudomonas_aeruginosa	*GCF_900144255.1*	*0*
Pseudomonas_aeruginosa	*GCF_900144265.1*	*0*
Pseudomonas_aeruginosa	*GCF_900144275.1*	*0*
Pseudomonas_aeruginosa	*GCF_900144285.1*	*0*
Pseudomonas_aeruginosa	*GCF_900144295.1*	*0*
Pseudomonas_aeruginosa	*GCF_900144305.1*	*0*
Pseudomonas_aeruginosa	*GCF_900144315.1*	*0*
Pseudomonas_aeruginosa	*GCF_900144325.1*	*0*
Pseudomonas_aeruginosa	*GCF_900144335.1*	*0*
Pseudomonas_aeruginosa	*GCF_900144345.1*	*0*
Pseudomonas_aeruginosa	*GCF_900144355.1*	*0*
Pseudomonas_aeruginosa	*GCF_900144365.1*	*0*
Pseudomonas_aeruginosa	*GCF_900144375.1*	*0*
Pseudomonas_aeruginosa	*GCF_900144385.1*	*0*
Pseudomonas_aeruginosa	*GCF_900144395.1*	*0*
Pseudomonas_aeruginosa	*GCF_900144405.1*	*0*
Pseudomonas_aeruginosa	*GCF_900144415.1*	*0*
Pseudomonas_aeruginosa	*GCF_900144425.1*	*0*
Pseudomonas_aeruginosa	*GCF_900144435.1*	*0*
Pseudomonas_aeruginosa	*GCF_900144445.1*	*0*
Pseudomonas_aeruginosa	*GCF_900144455.1*	*0*
Pseudomonas_aeruginosa	*GCF_900144465.1*	*0*
Pseudomonas_aeruginosa	*GCF_900144475.1*	*0*

Pseudomonas_aeruginosa	GCF_900144485.1	0	
Pseudomonas_aeruginosa	GCF_900144495.1	0	
Pseudomonas_aeruginosa	GCF_900144505.1	0	
Pseudomonas_aeruginosa	GCF_900144515.1	0	
Pseudomonas_aeruginosa	GCF_900144525.1	0	
Pseudomonas_aeruginosa	GCF_900144535.1	0	
Pseudomonas_aeruginosa	GCF_900144545.1	0	
Pseudomonas_aeruginosa	GCF_900144555.1	0	
Pseudomonas_aeruginosa	GCF_900144565.1	0	
Pseudomonas_aeruginosa	GCF_900144575.1	0	
Pseudomonas_aeruginosa	GCF_900144585.1	0	
Pseudomonas_aeruginosa	GCF_900144595.1	0	
Pseudomonas_aeruginosa	GCF_900144605.1	0	
Pseudomonas_aeruginosa	GCF_900144615.1	0	
Pseudomonas_aeruginosa	GCF_900144625.1	0	
Pseudomonas_aeruginosa	GCF_900144635.1	0	
Pseudomonas_aeruginosa	GCF_900144645.1	0	
Pseudomonas_aeruginosa	GCF_900144655.1	0	
Pseudomonas_aeruginosa	GCF_900144665.1	0	
Pseudomonas_aeruginosa	GCF_900144675.1	0	
Pseudomonas_aeruginosa	GCF_900144685.1	0	
Pseudomonas_aeruginosa	GCF_900144695.1	0	
Pseudomonas_aeruginosa	GCF_900144705.1	0	
Pseudomonas_aeruginosa	GCF_900144715.1	0	
Pseudomonas_aeruginosa	GCF_900144725.1	0	
Pseudomonas_aeruginosa	GCF_900144735.1	0	
Pseudomonas_aeruginosa	GCF_900144745.1	0	
Pseudomonas_aeruginosa	GCF_900144755.1	0	
Pseudomonas_aeruginosa	GCF_900144765.1	0	
Pseudomonas_aeruginosa	GCF_900144775.1	0	
Pseudomonas_aeruginosa	GCF_900144815.1	0	
Pseudomonas_aeruginosa	GCF_900144865.1	0	
Pseudomonas_aeruginosa	GCF_900144905.1	0	
Pseudomonas_aeruginosa	GCF_900144915.1	0	
Pseudomonas_aeruginosa	GCF_900144925.1	0	
Pseudomonas_aeruginosa	GCF_900144935.1	0	
Pseudomonas_aeruginosa	GCF_900144945.1	0	
Pseudomonas_aeruginosa	GCF_900144955.1	0	
Pseudomonas_aeruginosa	GCF_900144965.1	0	
Pseudomonas_aeruginosa	GCF_900144975.1	0	
Pseudomonas_aeruginosa	GCF_900144985.1	0	
Pseudomonas_aeruginosa	GCF_900144995.1	0	
Pseudomonas_aeruginosa	GCF_900145005.1	0	
Pseudomonas_aeruginosa	GCF_900145015.1	0	
Pseudomonas_aeruginosa	GCF_900145025.1	0	
Pseudomonas_aeruginosa	GCF_900145035.1	0	
Pseudomonas_aeruginosa	GCF_900145045.1	0	
Pseudomonas_aeruginosa	GCF_900145055.1	0	
Pseudomonas_aeruginosa	GCF_900145065.1	0	
Pseudomonas_aeruginosa	GCF_900145075.1	0	
Pseudomonas_aeruginosa	GCF_900145085.1	0	
Pseudomonas_aeruginosa	GCF_900145095.1	0	
Pseudomonas_aeruginosa	GCF_900145105.1	0	
Pseudomonas_aeruginosa	GCF_900145115.1	0	
Pseudomonas_aeruginosa	GCF_900145125.1	0	

Pseudomonas_aeruginosa	GCF_900145135.1	0	
Pseudomonas_aeruginosa	GCF_900145145.1	0	
Pseudomonas_aeruginosa	GCF_900145155.1	0	
Pseudomonas_aeruginosa	GCF_900145165.1	0	
Pseudomonas_aeruginosa	GCF_900145175.1	0	
Pseudomonas_aeruginosa	GCF_900145185.1	0	
Pseudomonas_aeruginosa	GCF_900145195.1	0	
Pseudomonas_aeruginosa	GCF_900145205.1	0	
Pseudomonas_aeruginosa	GCF_900145215.1	0	
Pseudomonas_aeruginosa	GCF_900145225.1	0	
Pseudomonas_aeruginosa	GCF_900145235.1	0	
Pseudomonas_aeruginosa	GCF_900145245.1	0	
Pseudomonas_aeruginosa	GCF_900145255.1	0	
Pseudomonas_aeruginosa	GCF_900145265.1	0	
Pseudomonas_aeruginosa	GCF_900145275.1	0	
Pseudomonas_aeruginosa	GCF_900145285.1	0	
Pseudomonas_aeruginosa	GCF_900145295.1	0	
Pseudomonas_aeruginosa	GCF_900145305.1	0	
Pseudomonas_aeruginosa	GCF_900145315.1	0	
Pseudomonas_aeruginosa	GCF_900145325.1	0	
Pseudomonas_aeruginosa	GCF_900145335.1	0	
Pseudomonas_aeruginosa	GCF_900145345.1	0	
Pseudomonas_aeruginosa	GCF_900145355.1	0	
Pseudomonas_aeruginosa	GCF_900145365.1	0	
Pseudomonas_aeruginosa	GCF_900145375.1	0	
Pseudomonas_aeruginosa	GCF_900145385.1	0	
Pseudomonas_aeruginosa	GCF_900145395.1	0	
Pseudomonas_aeruginosa	GCF_900145405.1	0	
Pseudomonas_aeruginosa	GCF_900145415.1	0	
Pseudomonas_aeruginosa	GCF_900145425.1	0	
Pseudomonas_aeruginosa	GCF_900145435.1	0	
Pseudomonas_aeruginosa	GCF_900145445.1	0	
Pseudomonas_aeruginosa	GCF_900145455.1	0	
Pseudomonas_aeruginosa	GCF_900145465.1	0	
Pseudomonas_aeruginosa	GCF_900145475.1	0	
Pseudomonas_aeruginosa	GCF_900145485.1	0	
Pseudomonas_aeruginosa	GCF_900145495.1	0	
Pseudomonas_aeruginosa	GCF_900145505.1	0	
Pseudomonas_aeruginosa	GCF_900145515.1	0	
Pseudomonas_aeruginosa	GCF_900145525.1	0	
Pseudomonas_aeruginosa	GCF_900145535.1	0	
Pseudomonas_aeruginosa	GCF_900145545.1	0	
Pseudomonas_aeruginosa	GCF_900145555.1	0	
Pseudomonas_aeruginosa	GCF_900145565.1	0	
Pseudomonas_aeruginosa	GCF_900145575.1	0	
Pseudomonas_aeruginosa	GCF_900145585.1	0	
Pseudomonas_aeruginosa	GCF_900145595.1	0	
Pseudomonas_aeruginosa	GCF_900145605.1	0	
Pseudomonas_aeruginosa	GCF_900145615.1	0	
Pseudomonas_aeruginosa	GCF_900145625.1	0	
Pseudomonas_aeruginosa	GCF_900145635.1	0	
Pseudomonas_aeruginosa	GCF_900145645.1	0	
Pseudomonas_aeruginosa	GCF_900145655.1	0	
Pseudomonas_aeruginosa	GCF_900145665.1	0	
Pseudomonas_aeruginosa	GCF_900145675.1	0	

Pseudomonas_aeruginosa	GCF_900145685.1	0
Pseudomonas_aeruginosa	GCF_900145695.1	0
Pseudomonas_aeruginosa	GCF_900145705.1	0
Pseudomonas_aeruginosa	GCF_900145715.1	0
Pseudomonas_aeruginosa	GCF_900145725.1	0
Pseudomonas_aeruginosa	GCF_900145735.1	0
Pseudomonas_aeruginosa	GCF_900145745.1	0
Pseudomonas_aeruginosa	GCF_900145755.1	0
Pseudomonas_aeruginosa	GCF_900145765.1	0
Pseudomonas_aeruginosa	GCF_900145775.1	0
Pseudomonas_aeruginosa	GCF_900145785.1	0
Pseudomonas_aeruginosa	GCF_900145795.1	0
Pseudomonas_aeruginosa	GCF_900145805.1	0
Pseudomonas_aeruginosa	GCF_900145815.1	0
Pseudomonas_aeruginosa	GCF_900145825.1	0
Pseudomonas_aeruginosa	GCF_900145835.1	0
Pseudomonas_aeruginosa	GCF_900145845.1	0
Pseudomonas_aeruginosa	GCF_900145855.1	0
Pseudomonas_aeruginosa	GCF_900145865.1	0
Pseudomonas_aeruginosa	GCF_900145875.1	0
Pseudomonas_aeruginosa	GCF_900145885.1	0
Pseudomonas_aeruginosa	GCF_900145895.1	0
Pseudomonas_aeruginosa	GCF_900145905.1	0
Pseudomonas_aeruginosa	GCF_900145915.1	0
Pseudomonas_aeruginosa	GCF_900145925.1	0
Pseudomonas_aeruginosa	GCF_900145935.1	0
Pseudomonas_aeruginosa	GCF_900145945.1	0
Pseudomonas_aeruginosa	GCF_900145955.1	0
Pseudomonas_aeruginosa	GCF_900145965.1	0
Pseudomonas_aeruginosa	GCF_900145975.1	0
Pseudomonas_aeruginosa	GCF_900145985.1	0
Pseudomonas_aeruginosa	GCF_900145995.1	0
Pseudomonas_aeruginosa	GCF_900146005.1	0
Pseudomonas_aeruginosa	GCF_900146015.1	0
Pseudomonas_aeruginosa	GCF_900146025.1	0
Pseudomonas_aeruginosa	GCF_900146035.1	0
Pseudomonas_aeruginosa	GCF_900146045.1	0
Pseudomonas_aeruginosa	GCF_900146055.1	0
Pseudomonas_aeruginosa	GCF_900146065.1	0
Pseudomonas_aeruginosa	GCF_900146075.1	0
Pseudomonas_aeruginosa	GCF_900146085.1	0
Pseudomonas_aeruginosa	GCF_900146105.1	0
Pseudomonas_aeruginosa	GCF_900146115.1	0
Pseudomonas_aeruginosa	GCF_900146125.1	0
Pseudomonas_aeruginosa	GCF_900146135.1	0
Pseudomonas_aeruginosa	GCF_900146145.1	0
Pseudomonas_aeruginosa	GCF_900146155.1	0
Pseudomonas_aeruginosa	GCF_900146165.1	0
Pseudomonas_aeruginosa	GCF_900146175.1	0
Pseudomonas_aeruginosa	GCF_900146185.1	0
Pseudomonas_aeruginosa	GCF_900146195.1	0
Pseudomonas_aeruginosa	GCF_900146205.1	0
Pseudomonas_aeruginosa	GCF_900146215.1	0
Pseudomonas_aeruginosa	GCF_900146225.1	0
Pseudomonas_aeruginosa	GCF_900146235.1	0

Pseudomonas_aeruginosa	GCF_900146245.1	0
Pseudomonas_aeruginosa	GCF_900146255.1	0
Pseudomonas_aeruginosa	GCF_900146265.1	0
Pseudomonas_aeruginosa	GCF_900146275.1	0
Pseudomonas_aeruginosa	GCF_900146285.1	0
Pseudomonas_aeruginosa	GCF_900146295.1	0
Pseudomonas_aeruginosa	GCF_900146305.1	0
Pseudomonas_aeruginosa	GCF_900146315.1	0
Pseudomonas_aeruginosa	GCF_900146325.1	0
Pseudomonas_aeruginosa	GCF_900146335.1	0
Pseudomonas_aeruginosa	GCF_900146345.1	0
Pseudomonas_aeruginosa	GCF_900146355.1	0
Pseudomonas_aeruginosa	GCF_900146365.1	0
Pseudomonas_aeruginosa	GCF_900146375.1	0
Pseudomonas_aeruginosa	GCF_900146385.1	0
Pseudomonas_aeruginosa	GCF_900146395.1	0
Pseudomonas_aeruginosa	GCF_900146405.1	0
Pseudomonas_aeruginosa	GCF_900146415.1	0
Pseudomonas_aeruginosa	GCF_900146425.1	0
Pseudomonas_aeruginosa	GCF_900146435.1	0
Pseudomonas_aeruginosa	GCF_900146445.1	0
Pseudomonas_aeruginosa	GCF_900146455.1	0
Pseudomonas_aeruginosa	GCF_900146465.1	0
Pseudomonas_aeruginosa	GCF_900146475.1	0
Pseudomonas_aeruginosa	GCF_900146485.1	0
Pseudomonas_aeruginosa	GCF_900146495.1	0
Pseudomonas_aeruginosa	GCF_900146505.1	0
Pseudomonas_aeruginosa	GCF_900146515.1	0
Pseudomonas_aeruginosa	GCF_900146525.1	0
Pseudomonas_aeruginosa	GCF_900146535.1	0
Pseudomonas_aeruginosa	GCF_900146545.1	0
Pseudomonas_aeruginosa	GCF_900146555.1	0
Pseudomonas_aeruginosa	GCF_900146565.1	0
Pseudomonas_aeruginosa	GCF_900146575.1	0
Pseudomonas_aeruginosa	GCF_900146585.1	0
Pseudomonas_aeruginosa	GCF_900146595.1	0
Pseudomonas_aeruginosa	GCF_900146605.1	0
Pseudomonas_aeruginosa	GCF_900146615.1	0
Pseudomonas_aeruginosa	GCF_900146625.1	0
Pseudomonas_aeruginosa	GCF_900146635.1	0
Pseudomonas_aeruginosa	GCF_900146645.1	0
Pseudomonas_aeruginosa	GCF_900146655.1	0
Pseudomonas_aeruginosa	GCF_900146665.1	0
Pseudomonas_aeruginosa	GCF_900146675.1	0
Pseudomonas_aeruginosa	GCF_900146685.1	0
Pseudomonas_aeruginosa	GCF_900146695.1	0
Pseudomonas_aeruginosa	GCF_900146705.1	0
Pseudomonas_aeruginosa	GCF_900146715.1	0
Pseudomonas_aeruginosa	GCF_900146725.1	0
Pseudomonas_aeruginosa	GCF_900146735.1	0
Pseudomonas_aeruginosa	GCF_900146745.1	0
Pseudomonas_aeruginosa	GCF_900146755.1	0
Pseudomonas_aeruginosa	GCF_900146765.1	0
Pseudomonas_aeruginosa	GCF_900146775.1	0
Pseudomonas_aeruginosa	GCF_900146785.1	0

Pseudomonas_aeruginosa	*GCF_900146795.1*	*0*
Pseudomonas_aeruginosa	*GCF_900146805.1*	*0*
Pseudomonas_aeruginosa	*GCF_900146815.1*	*0*
Pseudomonas_aeruginosa	*GCF_900146825.1*	*0*
Pseudomonas_aeruginosa	*GCF_900146835.1*	*0*
Pseudomonas_aeruginosa	*GCF_900146845.1*	*0*
Pseudomonas_aeruginosa	*GCF_900146855.1*	*0*
Pseudomonas_aeruginosa	*GCF_900146865.1*	*0*
Pseudomonas_aeruginosa	*GCF_900146875.1*	*0*
Pseudomonas_aeruginosa	*GCF_900146885.1*	*0*
Pseudomonas_aeruginosa	*GCF_900146895.1*	*0*
Pseudomonas_aeruginosa	*GCF_900146905.1*	*0*
Pseudomonas_aeruginosa	*GCF_900146915.1*	*0*
Pseudomonas_aeruginosa	*GCF_900146925.1*	*0*
Pseudomonas_aeruginosa	*GCF_900146935.1*	*0*
Pseudomonas_aeruginosa	*GCF_900146945.1*	*0*
Pseudomonas_aeruginosa	*GCF_900146955.1*	*0*
Pseudomonas_aeruginosa	*GCF_900146965.1*	*0*
Pseudomonas_aeruginosa	*GCF_900146975.1*	*0*
Pseudomonas_aeruginosa	*GCF_900146985.1*	*0*
Pseudomonas_aeruginosa	*GCF_900146995.1*	*0*
Pseudomonas_aeruginosa	*GCF_900147005.1*	*0*
Pseudomonas_aeruginosa	*GCF_900147015.1*	*0*
Pseudomonas_aeruginosa	*GCF_900147025.1*	*0*
Pseudomonas_aeruginosa	*GCF_900147035.1*	*0*
Pseudomonas_aeruginosa	*GCF_900147045.1*	*0*
Pseudomonas_aeruginosa	*GCF_900147055.1*	*0*
Pseudomonas_aeruginosa	*GCF_900147065.1*	*0*
Pseudomonas_aeruginosa	*GCF_900147075.1*	*0*
Pseudomonas_aeruginosa	*GCF_900147085.1*	*0*
Pseudomonas_aeruginosa	*GCF_900147095.1*	*0*
Pseudomonas_aeruginosa	*GCF_900147105.1*	*0*
Pseudomonas_aeruginosa	*GCF_900147115.1*	*0*
Pseudomonas_aeruginosa	*GCF_900147125.1*	*0*
Pseudomonas_aeruginosa	*GCF_900147135.1*	*0*
Pseudomonas_aeruginosa	*GCF_900147145.1*	*0*
Pseudomonas_aeruginosa	*GCF_900147155.1*	*0*
Pseudomonas_aeruginosa	*GCF_900147165.1*	*0*
Pseudomonas_aeruginosa	*GCF_900147175.1*	*0*
Pseudomonas_aeruginosa	*GCF_900147185.1*	*0*
Pseudomonas_aeruginosa	*GCF_900147195.1*	*0*
Pseudomonas_aeruginosa	*GCF_900147205.1*	*0*
Pseudomonas_aeruginosa	*GCF_900147215.1*	*0*
Pseudomonas_aeruginosa	*GCF_900147225.1*	*0*
Pseudomonas_aeruginosa	*GCF_900147235.1*	*0*
Pseudomonas_aeruginosa	*GCF_900147245.1*	*0*
Pseudomonas_aeruginosa	*GCF_900147255.1*	*0*
Pseudomonas_aeruginosa	*GCF_900147265.1*	*0*
Pseudomonas_aeruginosa	*GCF_900147275.1*	*0*
Pseudomonas_aeruginosa	*GCF_900147285.1*	*0*
Pseudomonas_aeruginosa	*GCF_900147295.1*	*0*
Pseudomonas_aeruginosa	*GCF_900147305.1*	*0*
Pseudomonas_aeruginosa	*GCF_900147315.1*	*0*
Pseudomonas_aeruginosa	*GCF_900147325.1*	*0*
Pseudomonas_aeruginosa	*GCF_900147335.1*	*0*

Pseudomonas_aeruginosa	*GCF_900147345.1*	*0*
Pseudomonas_aeruginosa	*GCF_900147355.1*	*0*
Pseudomonas_aeruginosa	*GCF_900147365.1*	*0*
Pseudomonas_aeruginosa	*GCF_900147375.1*	*0*
Pseudomonas_aeruginosa	*GCF_900147385.1*	*0*
Pseudomonas_aeruginosa	*GCF_900147395.1*	*0*
Pseudomonas_aeruginosa	*GCF_900147405.1*	*0*
Pseudomonas_aeruginosa	*GCF_900147415.1*	*0*
Pseudomonas_aeruginosa	*GCF_900147425.1*	*0*
Pseudomonas_aeruginosa	*GCF_900147435.1*	*0*
Pseudomonas_aeruginosa	*GCF_900147445.1*	*0*
Pseudomonas_aeruginosa	*GCF_900147455.1*	*0*
Pseudomonas_aeruginosa	*GCF_900147465.1*	*0*
Pseudomonas_aeruginosa	*GCF_900147475.1*	*0*
Pseudomonas_aeruginosa	*GCF_900147485.1*	*0*
Pseudomonas_aeruginosa	*GCF_900147495.1*	*0*
Pseudomonas_aeruginosa	*GCF_900147505.1*	*0*
Pseudomonas_aeruginosa	*GCF_900147515.1*	*0*
Pseudomonas_aeruginosa	*GCF_900147525.1*	*0*
Pseudomonas_aeruginosa	*GCF_900147535.1*	*0*
Pseudomonas_aeruginosa	*GCF_900147555.1*	*0*
Pseudomonas_aeruginosa	*GCF_900147565.1*	*0*
Pseudomonas_aeruginosa	*GCF_900147575.1*	*0*
Pseudomonas_aeruginosa	*GCF_900147585.1*	*0*
Pseudomonas_aeruginosa	*GCF_900147595.1*	*0*
Pseudomonas_aeruginosa	*GCF_900147605.1*	*0*
Pseudomonas_aeruginosa	*GCF_900147615.1*	*0*
Pseudomonas_aeruginosa	*GCF_900147625.1*	*0*
Pseudomonas_aeruginosa	*GCF_900147635.1*	*0*
Pseudomonas_aeruginosa	*GCF_900147645.1*	*0*
Pseudomonas_aeruginosa	*GCF_900147655.1*	*0*
Pseudomonas_aeruginosa	*GCF_900147665.1*	*0*
Pseudomonas_aeruginosa	*GCF_900147675.1*	*0*
Pseudomonas_aeruginosa	*GCF_900147685.1*	*0*
Pseudomonas_aeruginosa	*GCF_900147695.1*	*0*
Pseudomonas_aeruginosa	*GCF_900147705.1*	*0*
Pseudomonas_aeruginosa	*GCF_900147715.1*	*0*
Pseudomonas_aeruginosa	*GCF_900147725.1*	*0*
Pseudomonas_aeruginosa	*GCF_900147745.1*	*0*
Pseudomonas_aeruginosa	*GCF_900147755.1*	*0*
Pseudomonas_aeruginosa	*GCF_900147775.1*	*0*
Pseudomonas_aeruginosa	*GCF_900147785.1*	*0*
Pseudomonas_aeruginosa	*GCF_900147795.1*	*0*
Pseudomonas_aeruginosa	*GCF_900147805.1*	*0*
Pseudomonas_aeruginosa	*GCF_900147815.1*	*0*
Pseudomonas_aeruginosa	*GCF_900147825.1*	*0*
Pseudomonas_aeruginosa	*GCF_900147835.1*	*0*
Pseudomonas_aeruginosa	*GCF_900147845.1*	*0*
Pseudomonas_aeruginosa	*GCF_900147855.1*	*0*
Pseudomonas_aeruginosa	*GCF_900147865.1*	*0*
Pseudomonas_aeruginosa	*GCF_900147875.1*	*0*
Pseudomonas_aeruginosa	*GCF_900147885.1*	*0*
Pseudomonas_aeruginosa	*GCF_900147895.1*	*0*
Pseudomonas_aeruginosa	*GCF_900147905.1*	*0*
Pseudomonas_aeruginosa	*GCF_900147915.1*	*0*

Pseudomonas_aeruginosa	*GCF_900147925.1*	*0*	
Pseudomonas_aeruginosa	*GCF_900147935.1*	*0*	
Pseudomonas_aeruginosa	*GCF_900147945.1*	*0*	
Pseudomonas_aeruginosa	*GCF_900147955.1*	*0*	
Pseudomonas_aeruginosa	*GCF_900147965.1*	*0*	
Pseudomonas_aeruginosa	*GCF_900147975.1*	*0*	
Pseudomonas_aeruginosa	*GCF_900147985.1*	*0*	
Pseudomonas_aeruginosa	*GCF_900147995.1*	*0*	
Pseudomonas_aeruginosa	*GCF_900148005.1*	*0*	
Pseudomonas_aeruginosa	*GCF_900148015.1*	*0*	
Pseudomonas_aeruginosa	*GCF_900148025.1*	*0*	
Pseudomonas_aeruginosa	*GCF_900148035.1*	*0*	
Pseudomonas_aeruginosa	*GCF_900148045.1*	*0*	
Pseudomonas_aeruginosa	*GCF_900148055.1*	*0*	
Pseudomonas_aeruginosa	*GCF_900148065.1*	*0*	
Pseudomonas_aeruginosa	*GCF_900148075.1*	*0*	
Pseudomonas_aeruginosa	*GCF_900148085.1*	*0*	
Pseudomonas_aeruginosa	*GCF_900148095.1*	*0*	
Pseudomonas_aeruginosa	*GCF_900148105.1*	*0*	
Pseudomonas_aeruginosa	*GCF_900148115.1*	*0*	
Pseudomonas_aeruginosa	*GCF_900148125.1*	*0*	
Pseudomonas_aeruginosa	*GCF_900148135.1*	*0*	
Pseudomonas_aeruginosa	*GCF_900148145.1*	*0*	
Pseudomonas_aeruginosa	*GCF_900148155.1*	*0*	
Pseudomonas_aeruginosa	*GCF_900148165.1*	*0*	
Pseudomonas_aeruginosa	*GCF_900148175.1*	*0*	
Pseudomonas_aeruginosa	*GCF_900148185.1*	*0*	
Pseudomonas_aeruginosa	*GCF_900148195.1*	*0*	
Pseudomonas_aeruginosa	*GCF_900148205.1*	*0*	
Pseudomonas_aeruginosa	*GCF_900148215.1*	*0*	
Pseudomonas_aeruginosa	*GCF_900148225.1*	*0*	
Pseudomonas_aeruginosa	*GCF_900148235.1*	*0*	
Pseudomonas_aeruginosa	*GCF_900148245.1*	*0*	
Pseudomonas_aeruginosa	*GCF_900148255.1*	*0*	
Pseudomonas_aeruginosa	*GCF_900148265.1*	*0*	
Pseudomonas_aeruginosa	*GCF_900148275.1*	*0*	
Pseudomonas_aeruginosa	*GCF_900148285.1*	*0*	
Pseudomonas_aeruginosa	*GCF_900148295.1*	*0*	
Pseudomonas_aeruginosa	*GCF_900148305.1*	*0*	
Pseudomonas_aeruginosa	*GCF_900148315.1*	*0*	
Pseudomonas_aeruginosa	*GCF_900148325.1*	*0*	
Pseudomonas_aeruginosa	*GCF_900148335.1*	*0*	
Pseudomonas_aeruginosa	*GCF_900148345.1*	*0*	
Pseudomonas_aeruginosa	*GCF_900148355.1*	*0*	
Pseudomonas_aeruginosa	*GCF_900148365.1*	*0*	
Pseudomonas_aeruginosa	*GCF_900148375.1*	*0*	
Pseudomonas_aeruginosa	*GCF_900148385.1*	*0*	
Pseudomonas_aeruginosa	*GCF_900148395.1*	*0*	
Pseudomonas_aeruginosa	*GCF_900148405.1*	*0*	
Pseudomonas_aeruginosa	*GCF_900148415.1*	*0*	
Pseudomonas_aeruginosa	*GCF_900148425.1*	*0*	
Pseudomonas_aeruginosa	*GCF_900148435.1*	*0*	
Pseudomonas_aeruginosa	*GCF_900148445.1*	*0*	
Pseudomonas_aeruginosa	*GCF_900148455.1*	*0*	
Pseudomonas_aeruginosa	*GCF_900148465.1*	*0*	

Pseudomonas_aeruginosa	*GCF_900148475.1*	*0*
Pseudomonas_aeruginosa	*GCF_900148485.1*	*0*
Pseudomonas_aeruginosa_0C2E	*GCA_001500235.1*	*0*
Pseudomonas_aeruginosa_148	*GCA_000647595.1*	*0*
Pseudomonas_aeruginosa_19660	*GCA_000481765.1*	*0*
Pseudomonas_aeruginosa_19BR	*GCF_000223945.1*	*0*
Pseudomonas_aeruginosa_213BR	*GCF_000223965.1*	*0*
Pseudomonas_aeruginosa_3573	*GCA_000629325.1*	*0*
Pseudomonas_aeruginosa_3574	*GCA_000629305.1*	*0*
Pseudomonas_aeruginosa_3575	*GCA_000629285.1*	*0*
Pseudomonas_aeruginosa_3576	*GCA_000629265.1*	*0*
Pseudomonas_aeruginosa_3577	*GCA_000629245.1*	*0*
Pseudomonas_aeruginosa_3578	*GCA_000629225.1*	*0*
Pseudomonas_aeruginosa_3579	*GCA_000629205.1*	*0*
Pseudomonas_aeruginosa_3580	*GCA_000629185.1*	*0*
Pseudomonas_aeruginosa_3581	*GCA_000629165.1*	*0*
Pseudomonas_aeruginosa_6077	*GCA_000481745.1*	*0*
Pseudomonas_aeruginosa_62	*GCA_000482025.1*	*0*
Pseudomonas_aeruginosa_9BR	*GCF_000223925.1*	*0*
Pseudomonas_aeruginosa_AES-1R	*GCA_000220025.3*	*0*
Pseudomonas_aeruginosa_AH16	*GCF_000287875.1*	*0*
Pseudomonas_aeruginosa_ATCC_14886	*GCA_000297275.1*	*0*
Pseudomonas_aeruginosa_ATCC_15442	*GCF_000504485.1*	*0*
Pseudomonas_aeruginosa_ATCC_700888	*GCA_000297315.1*	*0*
Pseudomonas_aeruginosa_B136-33	*GCA_000359505.1*	*0*
Pseudomonas_aeruginosa_B3-1811	*GCF_000455545.1*	*0*
Pseudomonas_aeruginosa_B3-208	*GCF_000455405.1*	*0*
Pseudomonas_aeruginosa_B3-20M	*GCF_000455505.1*	*0*
Pseudomonas_aeruginosa_B3-CFI	*GCF_000455425.1*	*0*
Pseudomonas_aeruginosa_BL01	*GCA_000481125.1*	*0*
Pseudomonas_aeruginosa_BL02	*GCA_000481105.1*	*0*
Pseudomonas_aeruginosa_BL03	*GCA_000481085.1*	*0*
Pseudomonas_aeruginosa_BL04	*GCA_000481065.1*	*0*
Pseudomonas_aeruginosa_BL05	*GCA_000481045.1*	*0*
Pseudomonas_aeruginosa_BL06	*GCA_000481025.1*	*0*
Pseudomonas_aeruginosa_BL07	*GCA_000481005.1*	*0*
Pseudomonas_aeruginosa_BL08	*GCA_000480985.1*	*0*
Pseudomonas_aeruginosa_BL09	*GCA_000480965.1*	*0*
Pseudomonas_aeruginosa_BL10	*GCA_000480945.1*	*0*
Pseudomonas_aeruginosa_BL11	*GCA_000480925.1*	*0*
Pseudomonas_aeruginosa_BL12	*GCA_000480905.1*	*0*
Pseudomonas_aeruginosa_BL13	*GCA_000480885.1*	*0*
Pseudomonas_aeruginosa_BL14	*GCA_000480865.1*	*0*
Pseudomonas_aeruginosa_BL15	*GCA_000480845.1*	*0*
Pseudomonas_aeruginosa_BL16	*GCA_000480825.1*	*0*
Pseudomonas_aeruginosa_BL17	*GCA_000480805.1*	*0*
Pseudomonas_aeruginosa_BL18	*GCA_000480785.1*	*0*
Pseudomonas_aeruginosa_BL19	*GCA_000480765.1*	*0*
Pseudomonas_aeruginosa_BL20	*GCA_000480745.1*	*0*
Pseudomonas_aeruginosa_BL21	*GCA_000480725.1*	*0*
Pseudomonas_aeruginosa_BL22	*GCA_000480705.1*	*0*
Pseudomonas_aeruginosa_BL23	*GCA_000480685.1*	*0*
Pseudomonas_aeruginosa_BL24	*GCA_000480665.1*	*0*
Pseudomonas_aeruginosa_BL25	*GCA_000480645.1*	*0*
Pseudomonas_aeruginosa_BWH029	*GCA_000629145.1*	*0*

Pseudomonas_aeruginosa_BWH030	*GCA_000629125.1*	*0*
Pseudomonas_aeruginosa_BWH031	*GCA_000629105.1*	*0*
Pseudomonas_aeruginosa_BWH032	*GCA_000629085.1*	*0*
Pseudomonas_aeruginosa_BWH033	*GCA_000629065.1*	*0*
Pseudomonas_aeruginosa_BWH035	*GCA_000629045.1*	*0*
Pseudomonas_aeruginosa_BWH036	*GCA_000629025.1*	*0*
Pseudomonas_aeruginosa_BWH049	*GCA_000629605.1*	*0*
Pseudomonas_aeruginosa_BWH050	*GCA_000629585.1*	*0*
Pseudomonas_aeruginosa_BWH051	*GCA_000629565.1*	*0*
Pseudomonas_aeruginosa_BWH052	*GCA_000629545.1*	*0*
Pseudomonas_aeruginosa_BWH053	*GCA_000629525.1*	*0*
Pseudomonas_aeruginosa_BWH054	*GCA_000629505.1*	*0*
Pseudomonas_aeruginosa_BWH055	*GCA_000629485.1*	*0*
Pseudomonas_aeruginosa_BWH056	*GCA_000629465.1*	*0*
Pseudomonas_aeruginosa_BWH057	*GCA_000629445.1*	*0*
Pseudomonas_aeruginosa_BWH058	*GCA_000629425.1*	*0*
Pseudomonas_aeruginosa_BWH059	*GCA_000629405.1*	*0*
Pseudomonas_aeruginosa_BWH060	*GCA_000629385.1*	*0*
Pseudomonas_aeruginosa_BWHPSA001	*GCA_000481685.1*	*0*
Pseudomonas_aeruginosa_BWHPSA002	*GCA_000481665.1*	*0*
Pseudomonas_aeruginosa_BWHPSA003	*GCA_000481645.1*	*0*
Pseudomonas_aeruginosa_BWHPSA004	*GCA_000481625.1*	*0*
Pseudomonas_aeruginosa_BWHPSA005	*GCA_000481605.1*	*0*
Pseudomonas_aeruginosa_BWHPSA006	*GCA_000481585.1*	*0*
Pseudomonas_aeruginosa_BWHPSA007	*GCA_000481565.1*	*0*
Pseudomonas_aeruginosa_BWHPSA008	*GCA_000481545.1*	*0*
Pseudomonas_aeruginosa_BWHPSA009	*GCA_000481525.1*	*0*
Pseudomonas_aeruginosa_BWHPSA010	*GCA_000481505.1*	*0*
Pseudomonas_aeruginosa_BWHPSA011	*GCA_000481485.1*	*0*
Pseudomonas_aeruginosa_BWHPSA012	*GCA_000481465.1*	*0*
Pseudomonas_aeruginosa_BWHPSA013	*GCA_000481445.1*	*0*
Pseudomonas_aeruginosa_BWHPSA014	*GCA_000481425.1*	*0*
Pseudomonas_aeruginosa_BWHPSA015	*GCA_000481405.1*	*0*
Pseudomonas_aeruginosa_BWHPSA016	*GCA_000481385.1*	*0*
Pseudomonas_aeruginosa_BWHPSA017	*GCA_000481365.1*	*0*
Pseudomonas_aeruginosa_BWHPSA018	*GCA_000481345.1*	*0*
Pseudomonas_aeruginosa_BWHPSA019	*GCA_000481325.1*	*0*
Pseudomonas_aeruginosa_BWHPSA020	*GCA_000481305.1*	*0*
Pseudomonas_aeruginosa_BWHPSA021	*GCA_000481285.1*	*0*
Pseudomonas_aeruginosa_BWHPSA022	*GCA_000481265.1*	*0*
Pseudomonas_aeruginosa_BWHPSA023	*GCA_000481245.1*	*0*
Pseudomonas_aeruginosa_BWHPSA024	*GCA_000481225.1*	*0*
Pseudomonas_aeruginosa_BWHPSA025	*GCA_000481205.1*	*0*
Pseudomonas_aeruginosa_BWHPSA026	*GCA_000481185.1*	*0*
Pseudomonas_aeruginosa_BWHPSA027	*GCA_000481165.1*	*0*
Pseudomonas_aeruginosa_BWHPSA028	*GCA_000481145.1*	*0*
Pseudomonas_aeruginosa_BWHPSA037	*GCA_000520455.1*	*0*
Pseudomonas_aeruginosa_BWHPSA038	*GCA_000520435.1*	*0*
Pseudomonas_aeruginosa_BWHPSA039	*GCA_000520415.1*	*0*
Pseudomonas_aeruginosa_BWHPSA040	*GCA_000520395.1*	*0*
Pseudomonas_aeruginosa_BWHPSA041	*GCA_000520375.1*	*0*
Pseudomonas_aeruginosa_BWHPSA042	*GCA_000520355.1*	*0*
Pseudomonas_aeruginosa_BWHPSA043	*GCA_000520335.1*	*0*
Pseudomonas_aeruginosa_BWHPSA044	*GCA_000520315.1*	*0*
Pseudomonas_aeruginosa_BWHPSA045	*GCA_000520295.1*	*0*

Pseudomonas_aeruginosa_BWHPSA046	*GCA_000520275.1*	*0*
Pseudomonas_aeruginosa_BWHPSA047	*GCA_000520255.1*	*0*
Pseudomonas_aeruginosa_BWHPSA048	*GCA_000520235.1*	*0*
Pseudomonas_aeruginosa_C1913C	*GCA_000705155.1*	*0*
Pseudomonas_aeruginosa_C20	*GCA_000480515.1*	*0*
Pseudomonas_aeruginosa_C2159M	*GCA_000705215.1*	*0*
Pseudomonas_aeruginosa_C23	*GCA_000480495.1*	*0*
Pseudomonas_aeruginosa_C2773C	*GCA_000705175.1*	*0*
Pseudomonas_aeruginosa_C40	*GCA_000480475.1*	*0*
Pseudomonas_aeruginosa_C41	*GCA_000480455.1*	*0*
Pseudomonas_aeruginosa_C48	*GCA_000480435.1*	*0*
Pseudomonas_aeruginosa_C51	*GCA_000480415.1*	*0*
Pseudomonas_aeruginosa_C52	*GCA_000480395.1*	*0*
Pseudomonas_aeruginosa_c7447m	*GCA_000468935.1*	*0*
Pseudomonas_aeruginosa_CF_PA39	*GCF_000568235.2*	*0*
Pseudomonas_aeruginosa_CF127	*GCA_000481945.1*	*0*
Pseudomonas_aeruginosa_CF18	*GCA_000481925.1*	*0*
Pseudomonas_aeruginosa_CF27	*GCA_000481905.1*	*0*
Pseudomonas_aeruginosa_CF5	*GCA_000481885.1*	*0*
Pseudomonas_aeruginosa_CF614	*GCA_000480355.1*	*0*
Pseudomonas_aeruginosa_CF77	*GCA_000480375.1*	*0*
Pseudomonas_aeruginosa_CI27	*GCA_000297335.1*	*0*
Pseudomonas_aeruginosa_CIG1	*GCA_000295475.1*	*0*
Pseudomonas_aeruginosa_DHS01	*GCA_000496455.2*	*0*
Pseudomonas_aeruginosa_DHS29	*GCA_000503175.1*	*0*
Pseudomonas_aeruginosa_DK2	*GCA_000271365.1*	*0*
Pseudomonas_aeruginosa_DQ8	*GCF_000283055.1*	*0*
Pseudomonas_aeruginosa_DSM_50071	*GCA_001045685.1*	*0*
Pseudomonas_aeruginosa_DSM_50071	*GCA_900167195.1*	*0*
Pseudomonas_aeruginosa_E2	*GCA_000297355.1*	*0*
Pseudomonas_aeruginosa_E2	*GCA_000482005.1*	*0*
Pseudomonas_aeruginosa_H1l	*GCF_000633495.1*	*0*
Pseudomonas_aeruginosa_HB13	*GCA_000215775.5*	*0*
Pseudomonas_aeruginosa_HB15	*GCA_000215795.5*	*0*
Pseudomonas_aeruginosa_IGB83	*GCA_000647635.1*	*0*
Pseudomonas_aeruginosa_JD303	*GCF_000505885.1*	*0*
Pseudomonas_aeruginosa_JD304	*GCF_000505905.1*	*0*
Pseudomonas_aeruginosa_JD312	*GCF_000505945.1*	*0*
Pseudomonas_aeruginosa_JD315	*GCF_000505985.1*	*0*
Pseudomonas_aeruginosa_JD316	*GCF_000506125.1*	*0*
Pseudomonas_aeruginosa_JD317	*GCF_000506145.1*	*0*
Pseudomonas_aeruginosa_JD322	*GCF_000506185.1*	*0*
Pseudomonas_aeruginosa_JD323	*GCF_000506025.1*	*0*
Pseudomonas_aeruginosa_JD328	*GCF_000506065.1*	*0*
Pseudomonas_aeruginosa_JD329	*GCF_000506265.1*	*0*
Pseudomonas_aeruginosa_JD333	*GCF_000506345.1*	*0*
Pseudomonas_aeruginosa_JD335	*GCF_000506365.1*	*0*
Pseudomonas_aeruginosa_JJ692	*GCA_000481805.1*	*0*
Pseudomonas_aeruginosa_LCT-PA102	*GCF_000258285.1*	*0*
Pseudomonas_aeruginosa_LCT-PA220	*GCF_000439855.1*	*0*
Pseudomonas_aeruginosa_LCT-PA41	*GCF_000439875.1*	*0*
Pseudomonas_aeruginosa_LES400	*GCA_000583935.1*	*0*
Pseudomonas_aeruginosa_LES431	*GCA_000508765.1*	*0*
Pseudomonas_aeruginosa_LESB58	*GCA_000026645.1*	*0*
Pseudomonas_aeruginosa_LESB65	*GCA_000583955.1*	*0*

Pseudomonas_aeruginosa_LESlike4	*GCA_000583995.1*	*0*
Pseudomonas_aeruginosa_LESlike5	*GCA_000583895.1*	*0*
Pseudomonas_aeruginosa_LESlike7	*GCA_000583915.1*	*0*
Pseudomonas_aeruginosa_M18	*GCA_000226155.1*	*0*
Pseudomonas_aeruginosa_M8A.1	*GCA_000480615.1*	*0*
Pseudomonas_aeruginosa_M8A.2	*GCA_000480595.1*	*0*
Pseudomonas_aeruginosa_M8A.3	*GCA_000480575.1*	*0*
Pseudomonas_aeruginosa_M8A.4	*GCA_000480555.1*	*0*
Pseudomonas_aeruginosa_M9A.1	*GCA_000480535.1*	*0*
Pseudomonas_aeruginosa_MH27	*GCA_000513235.1*	*0*
Pseudomonas_aeruginosa_MH38	*GCA_000689435.1*	*0*
Pseudomonas_aeruginosa_MPAO1/P1	*GCA_000247435.2*	*0*
Pseudomonas_aeruginosa_MPAO1/P2	*GCA_000247455.2*	*0*
Pseudomonas_aeruginosa_MRSN_317	*GCA_000982125.1*	*0*
Pseudomonas_aeruginosa_MRW44.1	*GCF_000282915.1*	*0*
Pseudomonas_aeruginosa_MSH-10	*GCA_000407905.1*	*0*
Pseudomonas_aeruginosa_MSH10	*GCA_000481965.1*	*0*
Pseudomonas_aeruginosa_MSH3	*GCA_000481985.1*	*0*
Pseudomonas_aeruginosa_MTB-1	*GCA_000504045.1*	*0*
Pseudomonas_aeruginosa_MW3a	*GCF_000590905.1*	*0*
Pseudomonas_aeruginosa_N002	*GCF_000287815.1*	*0*
Pseudomonas_aeruginosa_NCAIM_B.001380	*GCF_000685845.1*	*0*
Pseudomonas_aeruginosa_P37	*GCA_002025525.1*	*0*
Pseudomonas_aeruginosa_P49	*GCA_002025515.1*	*0*
Pseudomonas_aeruginosa_PA1	*GCA_000496605.2*	*0*
Pseudomonas_aeruginosa_PA14	*GCA_000404265.1*	*0*
Pseudomonas_aeruginosa_PA21_ST175	*GCA_000342145.1*	*0*
Pseudomonas_aeruginosa_PA45	*GCA_000359565.1*	*0*
Pseudomonas_aeruginosa_PA96	*GCA_000626655.2*	*0*
Pseudomonas_aeruginosa_PAb1	*GCF_000172395.1*	*0*
Pseudomonas_aeruginosa_PABL056	*GCF_000290555.2*	*0*
Pseudomonas_aeruginosa_PACS2	*GCF_000168335.1*	*0*
Pseudomonas_aeruginosa_PAHM4	*GCF_001431165.1*	*0*
Pseudomonas_aeruginosa_PAK	*GCA_000408865.1*	*0*
Pseudomonas_aeruginosa_PAO1	*GCA_000006765.1*	*0*
Pseudomonas_aeruginosa_PAO1	*GCF_000414275.1*	*0*
Pseudomonas_aeruginosa_PAO1-GFP	*GCA_000629345.1*	*0*
Pseudomonas_aeruginosa_PAO1-VE13	*GCA_000484545.1*	*0*
Pseudomonas_aeruginosa_PAO1-VE2	*GCA_000484495.1*	*0*
Pseudomonas_aeruginosa_PAO579	*GCA_000296325.1*	*0*
Pseudomonas_aeruginosa_PAO581	*GCA_000468555.1*	*0*
Pseudomonas_aeruginosa_PS42	*GCA_000520195.1*	*0*
Pseudomonas_aeruginosa_PS50	*GCA_000520175.1*	*0*
Pseudomonas_aeruginosa_PS75	*GCA_000629365.1*	*0*
Pseudomonas_aeruginosa_RB-48	*GCF_000568115.1*	*0*
Pseudomonas_aeruginosa_RP73	*GCA_000414035.1*	*0*
Pseudomonas_aeruginosa_S35004	*GCA_000481725.1*	*0*
Pseudomonas_aeruginosa_S54485	*GCA_000481825.1*	*0*
Pseudomonas_aeruginosa_SD9	*GCA_002025565.1*	*0*
Pseudomonas_aeruginosa_SG17M	*GCA_000568215.1*	*0*
Pseudomonas_aeruginosa_SJTD-1	*GCA_000271985.2*	*0*
Pseudomonas_aeruginosa_str._C_1334	*GCF_000412295.1*	*0*
Pseudomonas_aeruginosa_str._C_1426	*GCF_000412555.1*	*0*
Pseudomonas_aeruginosa_str._C_763	*GCF_000412355.1*	*0*
Pseudomonas_aeruginosa_str._E2UoS	*GCF_000412395.1*	*0*

Pseudomonas_aeruginosa_str._J_1385	GCF_000412515.1	0
Pseudomonas_aeruginosa_str._MSH_10	GCF_000412435.1	0
Pseudomonas_aeruginosa_str._MSH_3	GCF_000412375.1	0
Pseudomonas_aeruginosa_str._PA_17	GCF_000412455.1	0
Pseudomonas_aeruginosa_str._PA_17SCV	GCF_000412495.1	0
Pseudomonas_aeruginosa_str._PA_62	GCF_000412415.1	0
Pseudomonas_aeruginosa_str._Stone_130	GCA_000478465.2	0
Pseudomonas_aeruginosa_U2504	GCA_000481785.1	0
Pseudomonas_aeruginosa_UCBPP-PA14	GCA_000014625.1	0
Pseudomonas_aeruginosa_UDL	GCA_000481845.1	0
Pseudomonas_aeruginosa_VRFPA02	GCA_000399805.1	0
Pseudomonas_aeruginosa_VRFPA04	GCA_000473745.3	0
Pseudomonas_aeruginosa_VRFPA05	GCA_000496325.1	0
Pseudomonas_aeruginosa_VRFPA06	GCA_000567865.1	0
Pseudomonas_aeruginosa_WS136	GCA_000820805.1	0
Pseudomonas_aeruginosa_WS394	GCA_000786485.1	0
Pseudomonas_aeruginosa_X13273	GCA_000481705.1	0
Pseudomonas_aeruginosa_X24509	GCA_000481865.1	0
Pseudomonas_aeruginosa_XMG	GCF_000265035.1	0
Pseudomonas_aeruginosa_YL84	GCA_000524595.1	0
Pseudomonas_aeruginosa_Z61	GCA_000520215.1	0
Pseudomonas_denitrificans	GCF_001060205.1	0
Pseudomonas_denitrificans	GCF_001060395.1	0
Pseudomonas_denitrificans	GCF_001060745.1	0
Pseudomonas_denitrificans	GCF_001061455.1	0
Pseudomonas_denitrificans	GCF_001062205.1	0
Pseudomonas_denitrificans	GCF_001062775.1	0
Pseudomonas_denitrificans	GCF_001062785.1	0
Pseudomonas_denitrificans	GCF_001063435.1	0
Pseudomonas_denitrificans	GCF_001063615.1	0
Pseudomonas_denitrificans	GCF_001064395.1	0
Pseudomonas_denitrificans	GCF_001065055.1	0
Pseudomonas_denitrificans	GCF_001065155.1	0
Pseudomonas_denitrificans	GCF_001066435.1	0
Pseudomonas_denitrificans	GCF_001066465.1	0
Pseudomonas_denitrificans	GCF_001067805.1	0
Pseudomonas_denitrificans	GCF_001067815.1	0
Pseudomonas_sp._2_1_26	GCA_000233495.1	0
Pseudomonas_sp._HMSC057H01	GCA_001836405.1	0
Pseudomonas_sp._HMSC058A10	GCA_001809935.1	0
Pseudomonas_sp._HMSC058B07	GCA_001836415.1	0
Pseudomonas_sp._HMSC058C05	GCA_001809775.1	0
Pseudomonas_sp._HMSC059F05	GCA_001836505.1	0
Pseudomonas_sp._HMSC060F12	GCA_001836625.1	0
Pseudomonas_sp._HMSC060G01	GCA_001836645.1	0
Pseudomonas_sp._HMSC060G02	GCA_001836655.1	0
Pseudomonas_sp._HMSC061A10	GCA_001836665.1	0
Pseudomonas_sp._HMSC063H08	GCA_001813645.1	0
Pseudomonas_sp._HMSC064G05	GCA_001810325.1	0
Pseudomonas_sp._HMSC065H01	GCA_001815235.1	0
Pseudomonas_sp._HMSC065H02	GCA_001813325.1	0
Pseudomonas_sp._HMSC066A08	GCA_001813025.1	0
Pseudomonas_sp._HMSC066B03	GCA_001811975.1	0
Pseudomonas_sp._HMSC067D05	GCA_001811215.1	0
Pseudomonas_sp._HMSC067F09	GCA_001812145.1	0

Pseudomonas_sp._HMSC067G02	*GCA_001813485.1*	*0*		
Pseudomonas_sp._HMSC069G05	*GCA_001813995.1*	*0*		
Pseudomonas_sp._HMSC070B12	*GCA_001837545.1*	*0*		
Pseudomonas_sp._HMSC071F02	*GCA_001837685.1*	*0*		
Pseudomonas_sp._HMSC072F09	*GCA_001809805.1*	*0*		
Pseudomonas_sp._HMSC073F05	*GCA_001814745.1*	*0*		
Pseudomonas_sp._HMSC075A08	*GCA_001810465.1*	*0*		
Pseudomonas_sp._HMSC076A11	*GCA_001810205.1*	*0*		
Pseudomonas_sp._HMSC076A12	*GCA_001812165.1*	*0*		
Pseudomonas_sp._HMSC11A05	*GCA_001807925.1*	*0*		
Pseudomonas_sp._HMSC16B01	*GCA_001808305.1*	*0*		
Pseudomonas_sp._P179	*GCA_000478485.2*	*0*		
Pseudomonas_putida_KT2440	*GCA_000007565.2*	*1*	x	x
Pseudomonas_viridiflava_CC1582	*GCF_000452505.1*	*2*	x	
[Pseudomonas_syringae]_pv._tomato_str._DC3000	*GCA_000007805.1*	*2*		
Pseudomonas_amygdali	*GCA_001400115.1*	*2*		
Pseudomonas_amygdali	*GCF_000935645.1*	*2*		
Pseudomonas_amygdali_pv._aesculi	*GCA_001400675.1*	*2*		
Pseudomonas_amygdali_pv._aesculi	*GCA_001537955.1*	*2*		
Pseudomonas_amygdali_pv._aesculi	*GCA_001537965.1*	*2*		
Pseudomonas_amygdali_pv._aesculi	*GCA_001537985.1*	*2*		
Pseudomonas_amygdali_pv._aesculi	*GCA_001538045.1*	*2*		
Pseudomonas_amygdali_pv._aesculi	*GCA_001538085.1*	*2*		
Pseudomonas_amygdali_pv._aesculi	*GCA_001538185.1*	*2*		
Pseudomonas_amygdali_pv._aesculi_str._0893_23	*GCA_000145685.1*	*2*		
Pseudomonas_amygdali_pv._aesculi_str._2250	*GCF_000163275.1*	*2*		
Pseudomonas_amygdali_pv._aesculi_str._NCPPB_3681	*GCF_000163255.1*	*2*		
Pseudomonas_amygdali_pv._ciccaronei	*GCA_001400755.1*	*2*		
Pseudomonas_amygdali_pv._dendropanacis	*GCA_001538145.1*	*2*		
Pseudomonas_amygdali_pv._dendropanacis	*GCF_000935665.1*	*2*		
Pseudomonas_amygdali_pv._dendropanacis	*GCF_000935735.1*	*2*		
Pseudomonas_amygdali_pv._eriobotryae	*GCA_001538055.1*	*2*		
Pseudomonas_amygdali_pv._hibisci	*GCA_001400395.1*	*2*		
Pseudomonas_amygdali_pv._lachrymans	*GCA_001006445.1*	*2*		
Pseudomonas_amygdali_pv._lachrymans	*GCA_001400475.1*	*2*		
Pseudomonas_amygdali_pv._lachrymans	*GCA_002068135.1*	*2*		
Pseudomonas_amygdali_pv._lachrymans_str._M301315	*GCA_000146005.1*	*2*		
Pseudomonas_amygdali_pv._lachrymans_str._M302278	*GCA_000145885.1*	*2*		
Pseudomonas_amygdali_pv._morsprunorum	*GCA_001535735.1*	*2*		
Pseudomonas_amygdali_pv._morsprunorum	*GCA_001535755.1*	*2*		
Pseudomonas_amygdali_pv._morsprunorum	*GCA_001535785.1*	*2*		
Pseudomonas_amygdali_pv._morsprunorum_str._M302280	*GCA_000145745.1*	*2*		
Pseudomonas_amygdali_pv._myricae	*GCA_001400555.1*	*2*		
Pseudomonas_amygdali_pv._myricae	*GCA_001535805.1*	*2*		
Pseudomonas_amygdali_pv._photiniae	*GCA_001400415.1*	*2*		
Pseudomonas_amygdali_pv._sesami	*GCA_001401265.1*	*2*		
Pseudomonas_amygdali_pv._tabaci	*GCA_000934645.1*	*2*		
Pseudomonas_amygdali_pv._tabaci_str._6605	*GCA_000275945.1*	*2*		
Pseudomonas_amygdali_pv._tabaci_str._ATCC_11528	*GCA_000159835.2*	*2*		
Pseudomonas_amygdali_pv._tabaci_str._ATCC_11528	*GCA_001006455.1*	*2*		
Pseudomonas_amygdali_pv._ulmi	*GCA_001401165.1*	*2*		

Pseudomonas_amygdali_pv._ulmi	*GCA_001535915.1*	*2*
Pseudomonas_asturiensis	*GCA_900143095.1*	*2*
Pseudomonas_avellanae	*GCF_000935825.1*	*2*
Pseudomonas_avellanae_BPIC_631	*GCF_000444135.1*	*2*
Pseudomonas_avellanae_CRAFRUec1	*GCF_000441975.1*	*2*
Pseudomonas_avellanae_CRAFRUed2	*GCF_000978625.2*	*2*
Pseudomonas_avellanae_CRAFRUee3	*GCF_000978635.2*	*2*
Pseudomonas_cannabina	*GCA_001400175.1*	*2*
Pseudomonas_caricapapayae	*GCA_001400735.1*	*2*
Pseudomonas_cerasi	*GCA_900074915.1*	*2*
Pseudomonas_congelans	*GCA_900103225.1*	*2*
Pseudomonas_coronafaciens_pv._garcae	*GCA_001400345.1*	*2*
Pseudomonas_coronafaciens_pv._porri	*GCA_001275725.1*	*2*
Pseudomonas_coronafaciens_pv._porri	*GCA_001275735.1*	*2*
Pseudomonas_coronafaciens_pv._porri	*GCA_001400915.1*	*2*
Pseudomonas_meliae	*GCF_000935675.1*	*2*
Pseudomonas_savastanoi_pv._fraxini	*GCA_001483545.1*	*2*
Pseudomonas_savastanoi_pv._fraxini	*GCA_001538155.1*	*2*
Pseudomonas_savastanoi_pv._glycinea_str._B076	*GCA_000187045.2*	*2*
Pseudomonas_savastanoi_pv._glycinea_str._race_4	*GCA_000187065.2*	*2*
Pseudomonas_savastanoi_pv._nerii	*GCA_001400575.1*	*2*
Pseudomonas_savastanoi_pv._nerii	*GCA_001535815.1*	*2*
Pseudomonas_savastanoi_pv._phaseolicola_1644R	*GCF_000225805.1*	*2*
Pseudomonas_savastanoi_pv._retacarpa	*GCA_001400935.1*	*2*
Pseudomonas_savastanoi_pv._savastanoi	*GCA_001401285.1*	*2*
Pseudomonas_savastanoi_pv._savastanoi	*GCF_000732035.1*	*2*
Pseudomonas_savastanoi_pv._savastanoi	*GCF_000751155.1*	*2*
Pseudomonas_savastanoi_pv._savastanoi	*GCF_000935695.1*	*2*
Pseudomonas_savastanoi_pv._savastanoi_NCPPB_3335	*GCA_000164015.3*	*2*
Pseudomonas_sp._ICMP_10191	*GCA_001467335.1*	*2*
Pseudomonas_sp._ICMP_3272	*GCA_001466905.1*	*2*
Pseudomonas_sp._NFACC10-1	*GCA_900119195.1*	*2*
Pseudomonas_syringae	*GCA_000737225.1*	*2*
Pseudomonas_syringae	*GCA_900103765.1*	*2*
Pseudomonas_syringae	*GCA_900106705.1*	*2*
Pseudomonas_syringae	*GCA_900107345.1*	*2*
Pseudomonas_syringae	*GCA_900113535.1*	*2*
Pseudomonas_syringae	*GCA_900113625.1*	*2*
Pseudomonas_syringae	*GCA_900114665.1*	*2*
Pseudomonas_syringae	*GCA_900115275.1*	*2*
Pseudomonas_syringae	*GCA_900115405.1*	*2*
Pseudomonas_syringae	*GCF_000800685.1*	*2*
Pseudomonas_syringae_BRIP34876	*GCA_000334035.1*	*2*
Pseudomonas_syringae_BRIP34881	*GCA_000334055.1*	*2*
Pseudomonas_syringae_BRIP39023	*GCA_000333995.1*	*2*
Pseudomonas_syringae_CC1416	*GCF_000452845.1*	*2*
Pseudomonas_syringae_CC1417	*GCF_000452825.1*	*2*
Pseudomonas_syringae_CC1458	*GCF_000452805.1*	*2*
Pseudomonas_syringae_CC1466	*GCF_000452785.1*	*2*
Pseudomonas_syringae_CC1513	*GCF_000452765.1*	*2*
Pseudomonas_syringae_CC1524	*GCF_000452745.1*	*2*
Pseudomonas_syringae_CC1543	*GCF_000452725.1*	*2*
Pseudomonas_syringae_CC1544	*GCF_000452905.1*	*2*
Pseudomonas_syringae_CC1557	*GCA_000452705.3*	*2*

Pseudomonas_syringae_CC1559	*GCF_000452685.1*	*2*	
Pseudomonas_syringae_CC1583	*GCF_000452665.1*	*2*	
Pseudomonas_syringae_CC1629	*GCF_000452645.1*	*2*	
Pseudomonas_syringae_CC1630	*GCF_000452625.1*	*2*	
Pseudomonas_syringae_CC440	*GCF_000452605.1*	*2*	
Pseudomonas_syringae_CC457	*GCF_000452585.1*	*2*	
Pseudomonas_syringae_CC94	*GCF_000452925.1*	*2*	
Pseudomonas_syringae_DSM_10604	*GCF_000597765.1*	*2*	
Pseudomonas_syringae_ICMP_11168	*GCA_001467285.1*	*2*	
Pseudomonas_syringae_ICMP_11292	*GCA_001467365.1*	*2*	
Pseudomonas_syringae_ICMP_11293	*GCA_001466845.1*	*2*	
Pseudomonas_syringae_ICMP_13102	*GCA_001466945.1*	*2*	
Pseudomonas_syringae_ICMP_18806	*GCF_000344415.1*	*2*	
Pseudomonas_syringae_ICMP_19498	*GCA_001467105.1*	*2*	
Pseudomonas_syringae_ICMP_19499	*GCA_001467115.1*	*2*	
Pseudomonas_syringae_KCTC_12500	*GCA_000507185.2*	*2*	
Pseudomonas_syringae_pv._aceris_str._M302273	*GCA_000145925.1*	*2*	
Pseudomonas_syringae_pv._actinidiae	*GCA_001910465.1*	*2*	
Pseudomonas_syringae_pv._actinidiae	*GCA_001910485.1*	*2*	
Pseudomonas_syringae_pv._actinidiae	*GCA_001910495.1*	*2*	
Pseudomonas_syringae_pv._actinidiae	*GCA_001910545.1*	*2*	
Pseudomonas_syringae_pv._actinidiae	*GCA_001910555.1*	*2*	
Pseudomonas_syringae_pv._actinidiae	*GCA_001913215.1*	*2*	
Pseudomonas_syringae_pv._actinidiae	*GCA_001913235.1*	*2*	
Pseudomonas_syringae_pv._actinidiae	*GCA_002024285.1*	*2*	
Pseudomonas_syringae_pv._actinidiae	*GCA_002024305.1*	*2*	
Pseudomonas_syringae_pv._actinidiae_CFBP_7286	*GCF_000245415.1*	*2*	
Pseudomonas_syringae_pv._actinidiae_CH2010-6	*GCF_000245475.1*	*2*	
Pseudomonas_syringae_pv._actinidiae_ICMP_18708	*GCA_000344355.2*	*2*	
Pseudomonas_syringae_pv._actinidiae_ICMP_18744	*GCF_000342185.1*	*2*	
Pseudomonas_syringae_pv._actinidiae_ICMP_18800	*GCF_000344375.1*	*2*	
Pseudomonas_syringae_pv._actinidiae_ICMP_18801	*GCA_000416945.1*	*2*	
Pseudomonas_syringae_pv._actinidiae_ICMP_18804	*GCF_000344395.1*	*2*	
Pseudomonas_syringae_pv._actinidiae_ICMP_18807	*GCA_000416625.1*	*2*	
Pseudomonas_syringae_pv._actinidiae_ICMP_18807	*GCF_000344535.1*	*2*	
Pseudomonas_syringae_pv._actinidiae_ICMP_18883	*GCA_000416785.2*	*2*	
Pseudomonas_syringae_pv._actinidiae_ICMP_18884	*GCA_000648735.3*	*2*	
Pseudomonas_syringae_pv._actinidiae_ICMP_18886	*GCA_000416925.1*	*2*	
Pseudomonas_syringae_pv._actinidiae_ICMP_19068	*GCA_000416705.1*	*2*	
Pseudomonas_syringae_pv._actinidiae_ICMP_19070	*GCA_000416685.1*	*2*	
Pseudomonas_syringae_pv._actinidiae_ICMP_19071	*GCA_000416485.1*	*2*	
Pseudomonas_syringae_pv._actinidiae_ICMP_19072	*GCA_000416885.1*	*2*	
Pseudomonas_syringae_pv._actinidiae_ICMP_19073	*GCA_000416505.1*	*2*	
Pseudomonas_syringae_pv._actinidiae_ICMP_19079	*GCA_000416605.1*	*2*	

Pseudomonas_syringae_pv._actinidiae_ICMP_19094	*GCA_000416745.1*	*2*	
Pseudomonas_syringae_pv._actinidiae_ICMP_19095	*GCA_000416765.1*	*2*	
Pseudomonas_syringae_pv._actinidiae_ICMP_19097	*GCA_000416725.1*	*2*	
Pseudomonas_syringae_pv._actinidiae_ICMP_19098	*GCA_000416545.1*	*2*	
Pseudomonas_syringae_pv._actinidiae_ICMP_19099	*GCA_000416805.1*	*2*	
Pseudomonas_syringae_pv._actinidiae_ICMP_19100	*GCA_000416825.2*	*2*	
Pseudomonas_syringae_pv._actinidiae_ICMP_19101	*GCA_000416585.1*	*2*	
Pseudomonas_syringae_pv._actinidiae_ICMP_19102	*GCA_000416845.2*	*2*	
Pseudomonas_syringae_pv._actinidiae_ICMP_19103	*GCA_000416525.1*	*2*	
Pseudomonas_syringae_pv._actinidiae_ICMP_19104	*GCA_000416865.1*	*2*	
Pseudomonas_syringae_pv._actinidiae_ICMP_19439	*GCF_000344555.1*	*2*	
Pseudomonas_syringae_pv._actinidiae_ICMP_19455	*GCF_000344515.2*	*2*	
Pseudomonas_syringae_pv._actinidiae_ICMP_19497	*GCA_001467225.1*	*2*	
Pseudomonas_syringae_pv._actinidiae_ICMP_9617	*GCA_000658965.1*	*2*	
Pseudomonas_syringae_pv._actinidiae_ICMP_9853	*GCA_000344335.2*	*2*	
Pseudomonas_syringae_pv._actinidiae_ICMP_9855	*GCA_000416665.1*	*2*	
Pseudomonas_syringae_pv._actinidiae_KW41	*GCF_000245435.1*	*2*	
Pseudomonas_syringae_pv._actinidiae_PA459	*GCF_000245455.1*	*2*	
Pseudomonas_syringae_pv._actinidiae_str._CRAFRU8.43	*GCF_000233815.1*	*2*	
Pseudomonas_syringae_pv._actinidiae_str._M302091	*GCA_000145865.1*	*2*	
Pseudomonas_syringae_pv._actinidiae_str._NCPPB_3739	*GCF_000233835.1*	*2*	
Pseudomonas_syringae_pv._actinidiae_str._NCPPB_3871	*GCF_000233795.1*	*2*	
Pseudomonas_syringae_pv._actinidiae_str._Shaanxi_M228	*GCF_000344475.2*	*2*	
Pseudomonas_syringae_pv._actinidiae_str._Shaanxi_M7	*GCF_000344495.1*	*2*	
Pseudomonas_syringae_pv._actinidiae_TP1	*GCF_000344435.1*	*2*	
Pseudomonas_syringae_pv._actinidiae_TP6-1	*GCF_000344455.1*	*2*	
Pseudomonas_syringae_pv._actinidifoliorum	*GCA_002003545.1*	*2*	
Pseudomonas_syringae_pv._actinidifoliorum	*GCF_001497455.1*	*2*	
Pseudomonas_syringae_pv._actinidifoliorum	*GCF_001497465.1*	*2*	
Pseudomonas_syringae_pv._actinidifoliorum	*GCF_001497495.1*	*2*	
Pseudomonas_syringae_pv._actinidifoliorum	*GCF_001497555.1*	*2*	
Pseudomonas_syringae_pv._actinidifoliorum	*GCF_001497595.1*	*2*	
Pseudomonas_syringae_pv._antirrhini	*GCA_001401395.1*	*2*	
Pseudomonas_syringae_pv._aptata	*GCA_001401335.1*	*2*	
Pseudomonas_syringae_pv._atrofaciens	*GCA_001400125.1*	*2*	
Pseudomonas_syringae_pv._atrofaciens_LMG_5095	*GCF_000710085.1*	*2*	
Pseudomonas_syringae_pv._atrofaciens_str._DSM_50255	*GCF_000498595.1*	*2*	
Pseudomonas_syringae_pv._avii	*GCA_001538095.1*	*2*	
Pseudomonas_syringae_pv._berberidis	*GCA_001401405.1*	*2*	
Pseudomonas_syringae_pv._broussonetiae	*GCA_001400715.1*	*2*	
Pseudomonas_syringae_pv._broussonetiae	*GCA_001538205.1*	*2*	
Pseudomonas_syringae_pv._castaneae	*GCA_001538245.1*	*2*	
Pseudomonas_syringae_pv._cerasicola	*GCA_001537945.1*	*2*	
Pseudomonas_syringae_pv._coriandricola	*GCA_001400185.1*	*2*	

Pseudomonas_syringae_pv._coryli_str._NCPPB_4273	*GCF_000972175.1*	*2*	
Pseudomonas_syringae_pv._cunninghamiae	*GCA_001400795.1*	*2*	
Pseudomonas_syringae_pv._daphniphylli	*GCA_001538255.1*	*2*	
Pseudomonas_syringae_pv._lapsa	*GCA_001482725.1*	*2*	
Pseudomonas_syringae_pv._maculicola	*GCA_001400855.1*	*2*	
Pseudomonas_syringae_pv._maculicola	*GCF_000935725.1*	*2*	
Pseudomonas_syringae_pv._papulans	*GCA_001535905.1*	*2*	
Pseudomonas_syringae_pv._papulans	*GCF_000935795.1*	*2*	
Pseudomonas_syringae_pv._persicae	*GCA_000980905.1*	*2*	
Pseudomonas_syringae_pv._pisi_str._PP1	*GCF_000452445.1*	*2*	
Pseudomonas_syringae_pv._rhaphiolepidis	*GCA_001535835.1*	*2*	
Pseudomonas_syringae_pv._solidagae	*GCA_001401055.1*	*2*	
Pseudomonas_syringae_pv._syringae	*GCA_000738515.1*	*2*	
Pseudomonas_syringae_pv._syringae	*GCA_001401075.1*	*2*	
Pseudomonas_syringae_pv._syringae	*GCA_001535725.1*	*2*	
Pseudomonas_syringae_pv._syringae	*GCA_001535855.1*	*2*	
Pseudomonas_syringae_pv._syringae	*GCA_001535875.1*	*2*	
Pseudomonas_syringae_pv._syringae	*GCA_001535945.1*	*2*	
Pseudomonas_syringae_pv._syringae	*GCA_001623415.1*	*2*	
Pseudomonas_syringae_pv._syringae	*GCA_001675375.1*	*2*	
Pseudomonas_syringae_pv._syringae	*GCA_001675415.1*	*2*	
Pseudomonas_syringae_pv._syringae	*GCF_000935775.1*	*2*	
Pseudomonas_syringae_pv._syringae_1212	*GCF_000452465.1*	*2*	
Pseudomonas_syringae_pv._syringae_642	*GCF_000177515.1*	*2*	
Pseudomonas_syringae_pv._syringae_B301D	*GCA_000988485.1*	*2*	
Pseudomonas_syringae_pv._syringae_B64	*GCA_000331385.1*	*2*	
Pseudomonas_syringae_pv._syringae_B728a	*GCA_000012245.1*	*2*	
Pseudomonas_syringae_pv._syringae_CRAFRU11	*GCF_000972155.1*	*2*	
Pseudomonas_syringae_pv._syringae_CRAFRU12	*GCF_000972195.1*	*2*	
Pseudomonas_syringae_pv._syringae_HS191	*GCA_000988395.1*	*2*	
Pseudomonas_syringae_pv._syringae_PD2766	*GCA_001466965.1*	*2*	
Pseudomonas_syringae_pv._syringae_PD2774	*GCA_001466875.1*	*2*	
Pseudomonas_syringae_pv._syringae_SM	*GCA_000412165.1*	*2*	
Pseudomonas_syringae_pv._syringae_str._B301D-R	*GCA_000585725.1*	*2*	
Pseudomonas_syringae_pv._tagetis	*GCA_001401315.1*	*2*	
Pseudomonas_syringae_pv._theae	*GCA_001401255.1*	*2*	
Pseudomonas_syringae_pv._theae_ICMP_3923	*GCA_000416465.2*	*2*	
Pseudomonas_syringae_pv._theae_NCPPB_2598	*GCF_000245395.1*	*2*	
Pseudomonas_syringae_pv._theae_NCPPB_2598	*GCF_000444115.1*	*2*	
Pseudomonas_syringae_pv._tomato	*GCA_000765305.1*	*2*	
Pseudomonas_syringae_pv._tomato	*GCA_001518465.1*	*2*	
Pseudomonas_syringae_pv._tomato	*GCA_001518695.1*	*2*	
Pseudomonas_syringae_pv._tomato	*GCA_002024925.1*	*2*	
Pseudomonas_syringae_pv._tomato_K40	*GCF_000177455.1*	*2*	
Pseudomonas_syringae_pv._tomato_Max13	*GCF_000177475.1*	*2*	
Pseudomonas_syringae_pv._tomato_NCPPB_1108	*GCF_000177495.1*	*2*	
Pseudomonas_syringae_pv._tomato_T1	*GCA_000172895.1*	*2*	
Pseudomonas_syringae_pv._viburni	*GCA_001401175.1*	*2*	
Pseudomonas_syringae_pv._viburni	*GCF_000935765.1*	*2*	
Pseudomonas_syringae_UB303	*GCF_000452565.1*	*2*	
Pseudomonas_syringae_UMAF0158	*GCA_001281365.1*	*2*	
Pseudomonas_syringae_USA007	*GCF_000452545.1*	*2*	
Pseudomonas_syringae_USA011	*GCF_000452525.2*	*2*	
Pseudomonas_trivialis	*GCF_001730655.1*	*3*	x

Pseudomonas_antarctica	*GCA_900103795.1*	*3*
Pseudomonas_azotoformans	*GCA_001870415.1*	*3*
Pseudomonas_azotoformans	*GCA_001983205.1*	*3*
Pseudomonas_azotoformans	*GCA_002007785.1*	*3*
Pseudomonas_azotoformans	*GCA_900103345.1*	*3*
Pseudomonas_azotoformans_NBRC_12693	*GCF_002091515.1*	*3*
Pseudomonas_canadensis	*GCF_000503215.1*	*3*
Pseudomonas_cedrina_subsp._cedrina	*GCA_001983175.1*	*3*
Pseudomonas_costantinii	*GCA_001870435.1*	*3*
Pseudomonas_costantinii	*GCA_900105935.1*	*3*
Pseudomonas_extremaustralis_14-3_substr._14-3b	*GCA_000242115.2*	*3*
Pseudomonas_extremorientalis	*GCA_001870465.1*	*3*
Pseudomonas_extremorientalis	*GCA_900104365.1*	*3*
Pseudomonas_fluorescens	*GCA_000730425.1*	*3*
Pseudomonas_fluorescens	*GCA_000876155.1*	*3*
Pseudomonas_fluorescens	*GCA_000934565.1*	*3*
Pseudomonas_fluorescens	*GCA_000967935.1*	*3*
Pseudomonas_fluorescens	*GCA_000968025.1*	*3*
Pseudomonas_fluorescens	*GCA_001466915.1*	*3*
Pseudomonas_fluorescens	*GCA_001541845.1*	*3*
Pseudomonas_fluorescens	*GCA_001542695.1*	*3*
Pseudomonas_fluorescens	*GCA_001542705.1*	*3*
Pseudomonas_fluorescens	*GCA_001542725.1*	*3*
Pseudomonas_fluorescens	*GCA_001602175.1*	*3*
Pseudomonas_fluorescens	*GCA_001747385.1*	*3*
Pseudomonas_fluorescens	*GCA_001908925.1*	*3*
Pseudomonas_fluorescens	*GCA_002022255.1*	*3*
Pseudomonas_fluorescens	*GCA_002022265.1*	*3*
Pseudomonas_fluorescens	*GCA_002022275.1*	*3*
Pseudomonas_fluorescens	*GCA_002022335.1*	*3*
Pseudomonas_fluorescens	*GCA_002022365.1*	*3*
Pseudomonas_fluorescens	*GCA_002022375.1*	*3*
Pseudomonas_fluorescens	*GCF_000708695.2*	*3*
Pseudomonas_fluorescens	*GCF_000801775.1*	*3*
Pseudomonas_fluorescens	*GCF_000801795.1*	*3*
Pseudomonas_fluorescens	*GCF_000801815.1*	*3*
Pseudomonas_fluorescens	*GCF_000801875.1*	*3*
Pseudomonas_fluorescens	*GCF_000801895.1*	*3*
Pseudomonas_fluorescens	*GCF_000803005.1*	*3*
Pseudomonas_fluorescens	*GCF_001902145.1*	*3*
Pseudomonas_fluorescens	*GCF_001931665.1*	*3*
Pseudomonas_fluorescens_BRIP34879	*GCA_000334015.1*	*3*
Pseudomonas_fluorescens_BS2	*GCF_000308175.1*	*3*
Pseudomonas_fluorescens_FH5	*GCA_000511155.2*	*3*
Pseudomonas_fluorescens_ICMP_11288	*GCA_001466835.1*	*3*
Pseudomonas_fluorescens_LMG_5329	*GCA_000411675.1*	*3*
Pseudomonas_fluorescens_NZ007	*GCF_000280805.1*	*3*
Pseudomonas_fluorescens_PA4C2	*GCF_000785395.1*	*3*
Pseudomonas_fluorescens_SBW25	*GCA_000009225.1*	*3*
Pseudomonas_fluorescens_SS101	*GCA_000263675.2*	*3*
Pseudomonas_gessardii	*GCA_001983165.1*	*3*
Pseudomonas_libanensis	*GCA_001297075.1*	*3*
Pseudomonas_libanensis	*GCA_001439685.1*	*3*
Pseudomonas_marginalis	*GCA_001645105.1*	*3*
Pseudomonas_marginalis_ICMP_9505	*GCA_001467265.1*	*3*

Pseudomonas_mucidolens_NBRC_103159	*GCF_002091735.1*	*3*
Pseudomonas_orientalis	*GCA_001439815.1*	*3*
Pseudomonas_poae	*GCA_001439785.1*	*3*
Pseudomonas_poae	*GCA_900103965.1*	*3*
Pseudomonas_poae	*GCF_001730605.1*	*3*
Pseudomonas_poae_RE*1-1-14	*GCA_000336465.1*	*3*
Pseudomonas_rhodesiae	*GCA_900105575.1*	*3*
Pseudomonas_salomonii	*GCA_900107155.1*	*3*
Pseudomonas_salomonii	*GCF_001730645.1*	*3*
Pseudomonas_simiae	*GCA_000698265.1*	*3*
Pseudomonas_simiae	*GCA_900111895.1*	*3*
Pseudomonas_simiae	*GCF_000785125.1*	*3*
Pseudomonas_simiae	*GCF_001730615.1*	*3*
Pseudomonas_sp._06C_126	*GCA_001855215.1*	*3*
Pseudomonas_sp._1_R_17	*GCA_900067035.1*	*3*
Pseudomonas_sp._24_E_1	*GCA_900005875.1*	*3*
Pseudomonas_sp._24_E_13	*GCA_900004715.1*	*3*
Pseudomonas_sp._24_R_17	*GCA_900004795.1*	*3*
Pseudomonas_sp._25_E_4	*GCA_900004645.1*	*3*
Pseudomonas_sp._25_R_14	*GCA_900004755.1*	*3*
Pseudomonas_sp._28_E_9	*GCA_900004785.1*	*3*
Pseudomonas_sp._31_E_5	*GCA_900005815.1*	*3*
Pseudomonas_sp._31_E_6	*GCA_900005935.1*	*3*
Pseudomonas_sp._31_R_17	*GCA_900004915.1*	*3*
Pseudomonas_sp._34_E_7	*GCA_900005715.1*	*3*
Pseudomonas_sp._35_E_8	*GCA_900005945.1*	*3*
Pseudomonas_sp._37_R_15	*GCA_900004865.1*	*3*
Pseudomonas_sp._44_R_15	*GCA_900004965.1*	*3*
Pseudomonas_sp._52_E_6	*GCA_900004655.1*	*3*
Pseudomonas_sp._58_R_12	*GCA_900005865.1*	*3*
Pseudomonas_sp._58_R_3	*GCA_900006605.1*	*3*
Pseudomonas_sp._8_R_14	*GCA_900005955.1*	*3*
Pseudomonas_sp._AP42	*GCA_001728935.1*	*3*
Pseudomonas_sp._BRG-100	*GCA_000737955.1*	*3*
Pseudomonas_sp._BTN1	*GCA_001907715.1*	*3*
Pseudomonas_sp._CBZ-4	*GCF_000346755.1*	*3*
Pseudomonas_sp._CFT9	*GCA_000416255.1*	*3*
Pseudomonas_sp._CHM02	*GCF_000612585.1*	*3*
Pseudomonas_sp._DSM_28142	*GCA_001439745.1*	*3*
Pseudomonas_sp._DSM_29164	*GCA_001439735.1*	*3*
Pseudomonas_sp._DSM_29167	*GCA_001439845.1*	*3*
Pseudomonas_sp._Eur1_9.41	*GCF_000744215.1*	*3*
Pseudomonas_sp._FH1	*GCA_000510895.2*	*3*
Pseudomonas_sp._FH4	*GCA_000510915.2*	*3*
Pseudomonas_sp._FSL_W5-0203	*GCA_001896155.1*	*3*
Pseudomonas_sp._ICMP_19500	*GCA_001467145.1*	*3*
Pseudomonas_sp._KG01	*GCA_001050345.1*	*3*
Pseudomonas_sp._LAMO17WK12:I2	*GCF_000514375.1*	*3*
Pseudomonas_sp._Leaf15	*GCA_001421425.1*	*3*
Pseudomonas_sp._MF6394	*GCA_002018845.1*	*3*
Pseudomonas_sp._MIACH	*GCF_001269925.1*	*3*
Pseudomonas_sp._NBRC_111137	*GCF_001320785.1*	*3*
Pseudomonas_sp._NBRC_111138	*GCF_001320835.1*	*3*
Pseudomonas_sp._NFPP02	*GCA_900119355.1*	*3*
Pseudomonas_sp._NFR02	*GCA_900119375.1*	*3*

Pseudomonas_sp._R81	*GCF_000257625.1*	*3*	
Pseudomonas_sp._RIT357	*GCA_000632245.1*	*3*	
Pseudomonas_sp._Root569	*GCA_001427465.1*	*3*	
Pseudomonas_sp._Root9	*GCA_001429205.1*	*3*	
Pseudomonas_sp._S3E12	*GCA_001702295.1*	*3*	
Pseudomonas_sp._WCS374	*GCA_000698295.1*	*3*	
Pseudomonas_synxantha	*GCA_001439725.1*	*3*	
Pseudomonas_synxantha	*GCA_002022245.1*	*3*	
Pseudomonas_synxantha_NBRC_3913	*GCF_002091795.1*	*3*	
Pseudomonas_tolaasii	*GCA_002072675.1*	*3*	
Pseudomonas_tolaasii_PMS117	*GCF_000276565.1*	*3*	
Pseudomonas_trivialis	*GCA_001186335.1*	*3*	
Pseudomonas_trivialis	*GCA_001439805.1*	*3*	
Pseudomonas_trivialis	*GCA_900104885.1*	*3*	
Pseudomonas_syringae	*GCA_900105295.1*	*4*	x
Pseudomonas_cannabina	*GCA_900100365.1*	*4*	
Pseudomonas_savastanoi_pv._phaseolicola_1448A	*GCA_000012205.1*	*4*	
Pseudomonas_protegens	*GCF_001269495.1*	*5*	x
Pseudomonas_chlororaphis	*GCA_000698865.1*	*5*	
Pseudomonas_chlororaphis	*GCA_900104985.1*	*5*	
Pseudomonas_chlororaphis_O6	*GCA_000264555.1*	*5*	
Pseudomonas_chlororaphis_subsp._aurantiaca	*GCA_000761195.1*	*5*	
Pseudomonas_chlororaphis_subsp._aurantiaca_PB-St2	*GCA_000506385.1*	*5*	
Pseudomonas_chlororaphis_subsp._aureofaciens	*GCF_001269575.1*	*5*	
Pseudomonas_chlororaphis_subsp._aureofaciens	*GCF_001269595.1*	*5*	
Pseudomonas_chlororaphis_subsp._aureofaciens_30-84	*GCA_000281915.1*	*5*	
Pseudomonas_chlororaphis_subsp._chlororaphis	*GCF_001269625.1*	*5*	
Pseudomonas_chlororaphis_subsp._chlororaphis_NBRC_3904	*GCF_002091535.1*	*5*	
Pseudomonas_chlororaphis_subsp._piscium	*GCA_001630695.1*	*5*	
Pseudomonas_chlororaphis_subsp._piscium	*GCF_001269555.1*	*5*	
Pseudomonas_protegens	*GCA_001904985.1*	*5*	
Pseudomonas_protegens	*GCA_001906605.1*	*5*	
Pseudomonas_protegens	*GCA_001906615.1*	*5*	
Pseudomonas_protegens	*GCA_001906625.1*	*5*	
Pseudomonas_protegens	*GCA_002006545.1*	*5*	
Pseudomonas_protegens	*GCF_001269465.1*	*5*	
Pseudomonas_protegens	*GCF_001269475.1*	*5*	
Pseudomonas_protegens	*GCF_001269485.1*	*5*	
Pseudomonas_protegens_Cab57	*GCA_000828695.1*	*5*	
Pseudomonas_protegens_CHA0	*GCA_000397205.1*	*5*	
Pseudomonas_protegens_Pf-5	*GCA_000012265.1*	*5*	
Pseudomonas_sp._BIOMIG1BAC	*GCF_001705995.1*	*5*	
Pseudomonas_sp._BIOMIG1BD	*GCF_001705885.1*	*5*	
Pseudomonas_sp._BIOMIG1BDMA	*GCF_001705845.1*	*5*	
Pseudomonas_sp._BIOMIG1N	*GCF_001705835.1*	*5*	
Pseudomonas_sp._CMR5c	*GCF_001269545.1*	*5*	
Pseudomonas_sp._NFPP09	*GCA_900119575.1*	*5*	
Pseudomonas_sp._NFPP14	*GCA_900119635.1*	*5*	
Pseudomonas_sp._NFPP16	*GCA_900119675.1*	*5*	
Pseudomonas_sp._Os17	*GCA_001547895.1*	*5*	
Pseudomonas_sp._St29	*GCA_001547915.1*	*5*	
Pseudomonas_putida	*GCA_900104105.1*	*6*	x
Pseudomonas_fluorescens	*GCA_000817905.1*	*6*	

Pseudomonas_fluorescens_Pf0-1	*GCA_000012445.1*	*6*		
Pseudomonas_sp._MS586	*GCA_001594225.1*	*6*		
Pseudomonas_oleovorans	*GCF_000732445.1*	*7*	x	
Pseudomonas_mendocina	*GCA_000725105.2*	*7*		
Pseudomonas_mendocina_NBRC_14162	*GCF_000813265.1*	*7*		
Pseudomonas_mendocina_NK-01	*GCA_000204295.1*	*7*		
Pseudomonas_mendocina_S5.2	*GCA_000733715.2*	*7*		
Pseudomonas_mendocina_ymp	*GCA_000016565.1*	*7*		
Pseudomonas_mendocina_ZWU0006	*GCF_000798915.1*	*7*		
Pseudomonas_sp._P818	*GCF_000418555.1*	*7*		
Pseudomonas_putida	*GCF_001293025.1*	*8*	x	x
Pseudomonas_monteilii	*GCF_000633915.1*	*8*		x
Pseudomonas_putida	*GCA_001077495.1*	*8*		x
Pseudomonas_putida	*GCA_001183585.1*	*8*		x
Pseudomonas_putida	*GCA_001653615.1*	*8*		x
Pseudomonas_putida	*GCA_001886975.1*	*8*		x
Pseudomonas_putida	*GCF_000787655.1*	*8*		x
Pseudomonas_putida	*GCF_000799625.1*	*8*		x
Pseudomonas_putida	*GCF_001282125.1*	*8*		x
Pseudomonas_putida_B6-2	*GCA_000226035.3*	*8*		x
Pseudomonas_putida_F1	*GCA_000016865.1*	*8*		x
Pseudomonas_putida_JB	*GCA_001767335.1*	*8*		x
Pseudomonas_putida_LF54	*GCA_000390005.2*	*8*		x
Pseudomonas_putida_LS46	*GCA_000294445.2*	*8*		x
Pseudomonas_putida_S12	*GCA_000495455.2*	*8*		x
Pseudomonas_putida_S12	*GCF_000287915.1*	*8*		x
Pseudomonas_putida_SJTE-1	*GCA_000271965.2*	*8*		x
Pseudomonas_putida_TRO1	*GCA_000367825.1*	*8*		x
Pseudomonas_sp._JY-Q	*GCA_001655295.1*	*8*		x
Pseudomonas_sp._NBRC_111118	*GCF_001320085.1*	*8*		x
Pseudomonas_sp._NBRC_111121	*GCF_001320165.1*	*8*		x
Pseudomonas_sp._NBRC_111125	*GCF_001320295.1*	*8*		x
Pseudomonas_sp._NBRC_111133	*GCF_001320605.1*	*8*		x
Pseudomonas_sp._NBRC_111136	*GCF_001320745.1*	*8*		x
Pseudomonas_sp._NBRC_111139	*GCF_001753955.1*	*8*		x
Pseudomonas_aeruginosa_PA7	*GCA_000017205.1*	*9*	x	
Pseudomonas_putida	*GCF_002025705.1*	*10*	x	x
Pseudomonas_fulva_NBRC_16637_=_DSM_17717	*GCF_000621265.1*	*10*		x
Pseudomonas_fulva_NBRC_16637_=_DSM_17717	*GCF_000730565.1*	*10*		x
Pseudomonas_monteilii	*GCA_001643215.1*	*10*		x
Pseudomonas_monteilii_NBRC_103158_=_DSM_14164	*GCF_000621245.1*	*10*		x
Pseudomonas_monteilii_NBRC_103158_=_DSM_14164	*GCF_000730605.1*	*10*		x
Pseudomonas_monteilii_QM	*GCF_000262005.1*	*10*		x
Pseudomonas_monteilii_SB3078	*GCA_000510285.1*	*10*		x
Pseudomonas_monteilii_SB3101	*GCA_000510325.1*	*10*		x
Pseudomonas_parafulva	*GCA_001477225.1*	*10*		x
Pseudomonas_parafulva	*GCA_001477425.1*	*10*		x
Pseudomonas_parafulva	*GCA_002021815.1*	*10*		x
Pseudomonas_parafulva	*GCF_001186195.1*	*10*		x
Pseudomonas_parafulva_NBRC_16636_=_DSM_17004	*GCF_000425765.1*	*10*		x
Pseudomonas_parafulva_NBRC_16636_=_DSM_17004	*GCF_000730645.1*	*10*		x
Pseudomonas_plecoglossicida_NB2011	*GCA_000412715.1*	*10*		x

Pseudomonas_plecoglossicida_NBRC_103162_=_DSM_15088	GCF_000730665.1	10		x
Pseudomonas_putida	GCA_001306515.1	10		x
Pseudomonas_putida	GCA_001630725.1	10		x
Pseudomonas_putida	GCA_001645635.1	10		x
Pseudomonas_putida	GCF_000875995.1	10		x
Pseudomonas_putida	GCF_001066335.1	10		x
Pseudomonas_putida	GCF_001750465.1	10		x
Pseudomonas_putida_B001	GCF_000285395.1	10		x
Pseudomonas_putida_GB-1	GCA_000019125.1	10		x
Pseudomonas_putida_H8234	GCA_000410575.1	10		x
Pseudomonas_putida_HB3267	GCA_000325725.1	10		x
Pseudomonas_putida_KG-4	GCA_000987155.1	10		x
Pseudomonas_putida_NBRC_14164	GCA_000412675.1	10		x
Pseudomonas_putida_OUS82	GCF_000507325.1	10		x
Pseudomonas_putida_S13.1.2	GCA_000498395.3	10		x
Pseudomonas_putida_T2-2	GCF_000710785.1	10		x
Pseudomonas_putida_W15Oct28	GCF_000708715.2	10		x
Pseudomonas_sp._10-1B	GCA_000935045.1	10		x
Pseudomonas_sp._DRA525	GCA_001908395.1	10		x
Pseudomonas_sp._GTC_16481	GCF_001753875.1	10		x
Pseudomonas_sp._GTC_16482	GCF_001319995.1	10		x
Pseudomonas_sp._LAIL14HWK12:I12	GCF_000514335.1	10		x
Pseudomonas_sp._LAIL14HWK12:I6	GCF_000514295.1	10		x
Pseudomonas_sp._LAIL14HWK12:I7	GCF_000483465.1	10		x
Pseudomonas_sp._LAIL14HWK12:I9	GCF_000514315.1	10		x
Pseudomonas_sp._LAMO17WK12:I4	GCF_000514355.1	10		x
Pseudomonas_sp._NBRC_111120	GCF_001753895.1	10		x
Pseudomonas_sp._NBRC_111122	GCF_001753915.1	10		x
Pseudomonas_sp._NBRC_111123	GCF_001320205.1	10		x
Pseudomonas_sp._NBRC_111124	GCF_001320245.1	10		x
Pseudomonas_sp._NBRC_111126	GCF_001753935.1	10		x
Pseudomonas_sp._NBRC_111129	GCF_001320435.1	10		x
Pseudomonas_sp._NBRC_111130	GCF_001320485.1	10		x
Pseudomonas_sp._NBRC_111131	GCF_001320525.1	10		x
Pseudomonas_sp._NBRC_111132	GCF_001320565.1	10		x
Pseudomonas_sp._NBRC_111134	GCF_001320655.1	10		x
Pseudomonas_sp._NBRC_111140	GCF_001320885.1	10		x
Pseudomonas_sp._NBRC_111144	GCF_001321005.1	10		x
Pseudomonas_sp._S13.1.2	GCF_000292285.1	10		x
Pseudomonas_sp._URIL14HWK12:I4	GCF_000483105.1	10		x
Pseudomonas_sp._URIL14HWK12:I5	GCA_900176445.1	10		x
Pseudomonas_sp._URMO17WK12:I11	GCA_001511775.1	10		x
Pseudomonas_sp._URMO17WK12:I11	GCF_000514235.1	10		x
Pseudomonas_sp._URMO17WK12:I8	GCF_000620365.1	10		x
Pseudomonas_putida	GCF_001005285.1	11	x	x
Pseudomonas_putida_W619	GCA_000019445.1	11		x
Pseudomonas_entomophila_L48	GCA_000026105.1	12	x	x
Pseudomonas_sp._CCOS_191	GCA_001007005.1	12		x
Pseudomonas_thivervalensis	GCF_001269685.1	13	x	
Pseudomonas_brassicacearum	GCA_000585995.1	13		
Pseudomonas_brassicacearum	GCA_001746815.1	13		
Pseudomonas_brassicacearum	GCA_900103245.1	13		
Pseudomonas_brassicacearum	GCF_000800585.1	13		
Pseudomonas_brassicacearum	GCF_001017815.1	13		
Pseudomonas_brassicacearum	GCF_001269635.1	13		

Pseudomonas_brassicacearum_51MFCVI2.1	*GCF_000510785.1*	*13*	
Pseudomonas_brassicacearum_PP1_210F	*GCF_000785375.1*	*13*	
Pseudomonas_brassicacearum_subsp._brassicacearum_NFM 421	*GCA_000194805.1*	*13*	
Pseudomonas_fluorescens	*GCA_000876175.1*	*13*	
Pseudomonas_fluorescens	*GCA_000952735.1*	*13*	
Pseudomonas_fluorescens	*GCA_001307275.1*	*13*	
Pseudomonas_fluorescens	*GCA_001623525.1*	*13*	
Pseudomonas_fluorescens	*GCF_001468775.2*	*13*	
Pseudomonas_fluorescens_Q8r1-96	*GCA_000263695.2*	*13*	
Pseudomonas_fluorescens_S12	*GCF_000498415.1*	*13*	
Pseudomonas_fluorescens_Wood1R	*GCF_000285615.1*	*13*	
Pseudomonas_frederiksbergensis	*GCA_000802155.2*	*13*	
Pseudomonas_kilonensis	*GCA_000968575.1*	*13*	
Pseudomonas_kilonensis	*GCA_900105635.1*	*13*	
Pseudomonas_kilonensis	*GCF_001269725.1*	*13*	
Pseudomonas_kilonensis	*GCF_001269885.1*	*13*	
Pseudomonas_sp._A25(2017)	*GCA_002001065.1*	*13*	
Pseudomonas_sp._CFII68	*GCA_000416195.1*	*13*	
Pseudomonas_sp._Ep_R1	*GCA_002027095.1*	*13*	
Pseudomonas_sp._NFACC04-2	*GCA_900119265.1*	*13*	
Pseudomonas_sp._NFACC16-2	*GCA_900119205.1*	*13*	
Pseudomonas_sp._Q12-87	*GCF_001269755.1*	*13*	
Pseudomonas_sp._Root401	*GCA_001425105.1*	*13*	
Pseudomonas_sp._URIL14HWK12:I7	*GCF_000514275.1*	*13*	
Pseudomonas_thivervalensis	*GCA_001637285.1*	*13*	
Pseudomonas_thivervalensis	*GCA_900102295.1*	*13*	
Pseudomonas_thivervalensis	*GCF_001269655.1*	*13*	
Pseudomonas_stutzeri_DSM_4166	*GCA_000195105.1*	*14*	x
Pseudomonas_fulva_12-X	*GCA_000213805.1*	*15*	x
Pseudomonas_argentinensis	*GCA_900113905.1*	*15*	
Pseudomonas_sp._PA1(2017)	*GCA_001945375.1*	*15*	
Pseudomonas_sp._PA15(2017)	*GCA_001945395.1*	*15*	
Pseudomonas_sp._PA27(2017)	*GCA_001945445.1*	*15*	
Pseudomonas_sp._URMO17WK12:I3	*GCF_000514255.1*	*15*	
Pseudomonas_sp._URMO17WK12:I4	*GCF_000514215.1*	*15*	
Pseudomonas_frederiksbergensis	*GCA_001874645.1*	*16*	x
Pseudomonas_fluorescens	*GCA_001307155.1*	*16*	
Pseudomonas_fluorescens_HK44	*GCA_000217955.3*	*16*	
Pseudomonas_psychrotolerans	*GCA_900102665.1*	*17*	x
Pseudomonas_oleovorans_MOIL14HWK12	*GCF_000510765.1*	*17*	
Pseudomonas_oryzihabitans	*GCA_000935305.1*	*17*	
Pseudomonas_oryzihabitans	*GCA_001518815.1*	*17*	
Pseudomonas_oryzihabitans	*GCA_001650425.1*	*17*	
Pseudomonas_oryzihabitans_NBRC_102199	*GCF_000730625.1*	*17*	
Pseudomonas_psychrotolerans	*GCA_001476385.1*	*17*	
Pseudomonas_psychrotolerans	*GCA_001476415.1*	*17*	
Pseudomonas_psychrotolerans	*GCA_001476435.1*	*17*	
Pseudomonas_psychrotolerans	*GCA_001476445.1*	*17*	
Pseudomonas_psychrotolerans	*GCA_001476475.1*	*17*	
Pseudomonas_psychrotolerans	*GCA_001476825.1*	*17*	
Pseudomonas_psychrotolerans	*GCA_001476875.1*	*17*	
Pseudomonas_psychrotolerans	*GCA_001477265.1*	*17*	
Pseudomonas_psychrotolerans	*GCA_001477295.1*	*17*	
Pseudomonas_psychrotolerans	*GCA_001477485.1*	*17*	
Pseudomonas_psychrotolerans	*GCA_001913135.1*	*17*	

Pseudomonas_psychrotolerans	*GCA_001991015.1*	*17*		
Pseudomonas_psychrotolerans_L19	*GCA_000236825.2*	*17*		
Pseudomonas_sp._313	*GCF_000316965.1*	*17*		
Pseudomonas_sp._MOIL14HWK12:I1	*GCF_000510705.1*	*17*		
Pseudomonas_sp._MOIL14HWK12:I2	*GCF_000510745.1*	*17*		
Pseudomonas_stutzeri	*GCA_000982865.1*	*18*	x	
Pseudomonas_sp._TTU2014-105ASC	*GCA_001446975.1*	*18*		
Pseudomonas_stutzeri	*GCA_000661915.1*	*18*		
Pseudomonas_stutzeri_ATCC_14405_=_CCUG_16156	*GCA_000237885.2*	*18*		
Pseudomonas_stutzeri_CCUG_29243	*GCA_000267545.1*	*18*		
Pseudomonas_pseudoalcaligenes_KF707_=_NBRC_110670	*GCA_000262065.3*	*19*	x	
Pseudomonas_synxantha	*GCA_900105675.1*	*20*	x	
Pseudomonas_fluorescens_EGD-AQ6	*GCA_000465595.1*	*20*		
Pseudomonas_fluorescens_PICF7	*GCA_000963495.1*	*20*		
Pseudomonas_libanensis	*GCA_900101035.1*	*20*		
Pseudomonas_marginalis	*GCA_900105325.1*	*20*		
Pseudomonas_orientalis	*GCA_900105795.1*	*20*		
Pseudomonas_sp._TKP	*GCA_000508205.1*	*20*		
Pseudomonas_synxantha_BG33R	*GCA_000263715.2*	*20*		
Pseudomonas_sp._M47T1	*GCA_000263855.1*	*21*	x	
Pseudomonas_putida_ND6	*GCA_000264665.1*	*22*	x	x
Pseudomonas_veronii_1YdBTEX2	*GCF_000350565.1*	*23*	x	
Pseudomonas_fluorescens_BBc6R8	*GCA_000297195.2*	*23*		
Pseudomonas_sp._Ag1	*GCA_000278565.1*	*23*		
Pseudomonas_sp._ATCC_PTA-122608	*GCA_001952855.1*	*23*		
Pseudomonas_sp._PAMC_25886	*GCF_000242655.1*	*23*		
Pseudomonas_sp._PAMC_26793	*GCF_000313235.1*	*23*		
Pseudomonas_veronii	*GCA_002028325.1*	*23*		
Pseudomonas_xanthomarina	*GCA_001705225.1*	*24*	x	
Pseudomonas_sp._TTU2014-066ASC	*GCA_001446915.1*	*24*		
Pseudomonas_sp._TTU2014-096BSC	*GCA_001446945.1*	*24*		
Pseudomonas_stutzeri_DSM_10701	*GCA_000279165.1*	*24*		
Pseudomonas_fluorescens_Q2-87	*GCA_000281895.1*	*25*	x	
Pseudomonas_sp._RIT-PI-o	*GCA_001297215.1*	*26*	x	
Pseudomonas_chlororaphis	*GCA_000783395.1*	*26*		
Pseudomonas_fluorescens	*GCA_000817895.1*	*26*		
Pseudomonas_fluorescens	*GCA_000834545.1*	*26*		
Pseudomonas_fluorescens	*GCF_001020865.1*	*26*		
Pseudomonas_koreensis	*GCA_001605965.1*	*26*		
Pseudomonas_koreensis	*GCA_001654515.1*	*26*		
Pseudomonas_koreensis	*GCA_001856885.1*	*26*		
Pseudomonas_koreensis	*GCA_002003425.1*	*26*		
Pseudomonas_koreensis	*GCA_900101415.1*	*26*		
Pseudomonas_moraviensis	*GCA_001896125.1*	*26*		
Pseudomonas_moraviensis	*GCA_900105805.1*	*26*		
Pseudomonas_moraviensis	*GCF_001691585.1*	*26*		
Pseudomonas_sp._B10	*GCA_900156235.1*	*26*		
Pseudomonas_sp._GM30	*GCA_000282275.2*	*26*		
Pseudomonas_sp._H1h	*GCF_000633255.1*	*26*		
Pseudomonas_sp._Leaf434	*GCA_001425545.1*	*26*		
Pseudomonas_sp._PTA1	*GCF_000745605.1*	*26*		
Pseudomonas_sp._R62	*GCF_000257605.1*	*26*		
Pseudomonas_sp._RIT-PI-r	*GCA_001297015.1*	*26*		
Pseudomonas_sp._RIT288	*GCA_000631985.1*	*26*		

Pseudomonas_sp._URIL14HWK12:I6	*GCF_000514195.1*	*26*	
Pseudomonas_sp._W15Feb9B	*GCA_000828175.1*	*26*	
Pseudomonas_syringae	*GCA_001238485.1*	*27*	x
Pseudomonas_fluorescens	*GCA_000967955.1*	*27*	
Pseudomonas_fluorescens	*GCA_000968015.1*	*27*	
Pseudomonas_fluorescens	*GCA_001625455.1*	*27*	
Pseudomonas_fluorescens_NCIMB_11764	*GCA_000293885.3*	*27*	
Pseudomonas_frederiksbergensis	*GCA_001952935.1*	*27*	
Pseudomonas_frederiksbergensis	*GCA_900105495.1*	*27*	
Pseudomonas_mandelii_PD30	*GCA_000690555.2*	*27*	
Pseudomonas_sp._655	*GCA_001280445.1*	*27*	
Pseudomonas_sp._A3(2016)	*GCA_001661075.1*	*27*	
Pseudomonas_sp._Ant30-3	*GCF_000690905.1*	*27*	
Pseudomonas_sp._ES3-33	*GCA_000952175.1*	*27*	
Pseudomonas_sp._FSL_W5-0299	*GCA_002005125.1*	*27*	
Pseudomonas_sp._GM41(2012)	*GCA_000282315.2*	*27*	
Pseudomonas_sp._In5	*GCA_001308855.1*	*27*	
Pseudomonas_sp._RIT-PI-q	*GCA_001297125.1*	*27*	
Pseudomonas_sp._Root329	*GCA_001424925.1*	*27*	
Pseudomonas_pseudoalcaligenes_NBRC_14167	*GCF_002091775.1*	*28*	x
Pseudomonas_composti	*GCA_900115475.1*	*28*	
Pseudomonas_mendocina_EGD-AQ5	*GCA_000465575.1*	*28*	
Pseudomonas_oleovorans	*GCA_900109155.1*	*28*	
Pseudomonas_oleovorans_subsp._oleovorans_NBRC_13583	*GCF_002091815.1*	*28*	
Pseudomonas_pseudoalcaligenes	*GCA_000953455.1*	*28*	
Pseudomonas_pseudoalcaligenes_AD6	*GCA_000626735.1*	*28*	
Pseudomonas_pseudoalcaligenes_CECT_5344	*GCA_000297075.2*	*28*	
Pseudomonas_aeruginosa_JD334	*GCF_000506285.1*	*29*	x
Pseudomonas_aeruginosa_ATCC_25324	*GCA_000297295.1*	*29*	
Pseudomonas_aeruginosa_JD318	*GCF_000506005.1*	*29*	
Pseudomonas_aeruginosa_JD324	*GCF_000506205.1*	*29*	
Pseudomonas_stutzeri	*GCF_001294145.1*	*30*	x
Pseudomonas_sp._Chol1	*GCA_000306015.1*	*30*	
Pseudomonas_sp._HMP271	*GCA_000765155.1*	*30*	
Pseudomonas_stutzeri	*GCA_001996325.1*	*30*	
Pseudomonas_viridiflava	*GCA_001716855.1*	*31*	x
Pseudomonas_marginalis_ICMP_11289	*GCA_001467325.1*	*31*	
Pseudomonas_sp._GEV388	*GCA_002087235.1*	*31*	
Pseudomonas_syringae_pv._ribicola	*GCA_001400955.1*	*31*	
Pseudomonas_viridiflava	*GCA_001401215.1*	*31*	
Pseudomonas_viridiflava_UASWS0038	*GCA_000307715.1*	*31*	
Pseudomonas_stutzeri_KOS6	*GCA_000307775.2*	*32*	x
Pseudomonas_reinekei	*GCA_001945365.1*	*33*	x
Pseudomonas_fluorescens	*GCA_000802965.1*	*33*	
Pseudomonas_fluorescens	*GCA_000967965.1*	*33*	
Pseudomonas_fluorescens	*GCA_001440325.1*	*33*	
Pseudomonas_putida	*GCA_000729805.1*	*33*	
Pseudomonas_putida	*GCA_001006135.1*	*33*	
Pseudomonas_putida	*GCA_001976065.1*	*33*	
Pseudomonas_sp._11/12A	*GCF_000800055.1*	*33*	
Pseudomonas_sp._KK4	*GCF_001984065.1*	*33*	
Pseudomonas_sp._Leaf48	*GCA_001421885.1*	*33*	
Pseudomonas_sp._Root562	*GCA_001427125.1*	*33*	
Pseudomonas_sp._Root68	*GCA_001427765.1*	*33*	
Pseudomonas_sp._Root71	*GCA_001429045.1*	*33*	

Pseudomonas_sp._UW4	*GCA_000316175.1*	*33*		
Pseudomonas_xanthomarina	*GCA_000825645.1*	*34*	x	
Pseudomonas_stutzeri	*GCA_000935215.1*	*34*		
Pseudomonas_stutzeri_NF13	*GCA_000341615.1*	*34*		
Pseudomonas_psychrophila	*GCA_900106105.1*	*35*	x	
Pseudomonas_deceptionensis	*GCA_001042895.1*	*35*		
Pseudomonas_fragi	*GCA_001543265.1*	*35*		
Pseudomonas_fragi_A22	*GCF_000250595.1*	*35*		
Pseudomonas_psychrophila	*GCA_001005765.1*	*35*		
Pseudomonas_psychrophila	*GCA_001043005.1*	*35*		
Pseudomonas_sp._A4R1.12	*GCA_001901345.1*	*35*		
Pseudomonas_sp._A4R1.5	*GCA_001901355.1*	*35*		
Pseudomonas_sp._CF149	*GCA_000416155.1*	*35*		
Pseudomonas_sp._L10.10	*GCA_001294575.1*	*35*		
Pseudomonas_sp._Lz4W	*GCA_000346225.1*	*35*		
Pseudomonas_nitroreducens_NBRC_12694	*GCF_002091755.1*	*36*	x	
Pseudomonas_denitrificans_ATCC_13867	*GCA_000349845.1*	*36*		
Pseudomonas_nitroreducens	*GCF_000807755.1*	*36*		
Pseudomonas_nitroreducens_TX1	*GCF_000313755.1*	*36*		
Pseudomonas_sp._21	*GCA_000955805.1*	*36*		
Pseudomonas_sp._AU11447	*GCA_001672555.2*	*36*		
Pseudomonas_sp._AU12215	*GCA_001675465.2*	*36*		
Pseudomonas_sp._T	*GCA_002079985.1*	*36*		
Pseudomonas_sp._G5(2012)	*GCA_000408945.1*	*37*	x	
Pseudomonas_resinovorans_NBRC_106553	*GCA_000412695.1*	*38*	x	
Pseudomonas_syringae	*GCA_000737235.1*	*39*	x	
Pseudomonas_sp._CFII64	*GCA_000416235.1*	*39*		
Pseudomonas_stutzeri	*GCF_001064225.1*	*40*	x	
Pseudomonas_stutzeri	*GCA_001276475.1*	*40*		
Pseudomonas_stutzeri	*GCA_001575085.1*	*40*		
Pseudomonas_stutzeri	*GCA_001705635.1*	*40*		
Pseudomonas_stutzeri	*GCA_002027175.1*	*40*		
Pseudomonas_stutzeri	*GCF_000952205.1*	*40*		
Pseudomonas_stutzeri	*GCF_001062345.1*	*40*		
Pseudomonas_stutzeri_B1SMN1	*GCA_000416345.1*	*40*		
Pseudomonas_stutzeri_T13	*GCF_000282955.1*	*40*		
Pseudomonas_stutzeri_XLDN-R	*GCF_000280555.1*	*40*		
Pseudomonas_stutzeri	*GCA_000952685.1*	*41*	x	
Pseudomonas_stutzeri	*GCA_000590475.1*	*41*		
Pseudomonas_stutzeri_MF28	*GCA_000455665.1*	*41*		
Pseudomonas_alcaligenes	*GCA_900156545.1*	*42*	x	
Pseudomonas_alcaligenes	*GCA_001597285.1*	*42*		
Pseudomonas_alcaligenes	*GCA_900156135.1*	*42*		
Pseudomonas_alcaligenes_NBRC_14159	*GCA_000467105.1*	*42*		
Pseudomonas_zeshuii	*GCA_900141925.1*	*43*	x	
Pseudomonas_lutea	*GCA_900110795.1*	*43*		
Pseudomonas_luteola_NBRC_103146	*GCF_002091675.1*	*43*		
Pseudomonas_sp._HPB0071	*GCA_000478505.2*	*43*		
Pseudomonas_taiwanensis_DSM_21245	*GCF_000425785.1*	*44*	x	x
Pseudomonas_sp._VLB120	*GCA_000494915.1*	*44*		x
Pseudomonas_taeanensis_MS-3	*GCA_000498575.2*	*45*	x	
Pseudomonas_cichorii_JBC1	*GCA_000517305.1*	*46*	x	
Pseudomonas_bauzanensis	*GCA_000632535.1*	*47*	x	
Pseudomonas_knackmussii_B13	*GCA_000689415.1*	*48*	x	
Pseudomonas_citronellolis_NBRC_103043	*GCF_002091555.1*	*49*	x	

Pseudomonas_citronellolis	*GCA_001446495.1*	*49*		
Pseudomonas_citronellolis	*GCA_001446995.1*	*49*		
Pseudomonas_citronellolis	*GCA_001586155.1*	*49*		
Pseudomonas_citronellolis	*GCA_001654435.1*	*49*		
Pseudomonas_citronellolis	*GCA_900112375.1*	*49*		
Pseudomonas_sp._AAC	*GCA_000725445.1*	*49*		
Pseudomonas_sp._CCA_1	*GCF_001748265.1*	*49*		
Pseudomonas_sp._EGD-AKN5	*GCA_001669755.1*	*49*		
Pseudomonas_sp._HMSC75E02	*GCA_001838695.1*	*49*		
Pseudomonas_sp._H2	*GCA_000763225.1*	*50*	x	x
Pseudomonas_capeferrum	*GCA_000731675.1*	*50*		x
Pseudomonas_syringae_UB246	*GCF_000452865.1*	*51*	x	
Pseudomonas_syringae	*GCA_000737245.1*	*51*		
Pseudomonas_syringae	*GCA_001698815.1*	*51*		
Pseudomonas_vranovensis_DSM_16006	*GCF_000425805.1*	*52*	x	x
Pseudomonas_alkylphenolica	*GCA_000746525.1*	*52*		x
Pseudomonas_sp._5	*GCA_000955815.1*	*52*		x
Pseudomonas_saudiphocaensis	*GCA_000756775.2*	*53*	x	
Pseudomonas_lutea	*GCA_000759445.1*	*54*	x	
Pseudomonas_graminis	*GCA_001705435.1*	*54*		
Pseudomonas_cremoricolorata_DSM_17059_=_NBRC_16634	*GCF_000425745.1*	*55*	x	x
Pseudomonas_cremoricolorata	*GCA_000759535.1*	*55*		x
Pseudomonas_rhizosphaerae	*GCA_000761155.1*	*56*	x	
Pseudomonas_sp._Leaf129	*GCA_001423465.1*	*56*		
Pseudomonas_sp._RIT-PI-a	*GCA_001187875.1*	*56*		
Pseudomonas_mediterranea	*GCF_001412185.1*	*57*	x	
Pseudomonas_corrugata	*GCA_001708425.1*	*57*		
Pseudomonas_corrugata	*GCA_900106085.1*	*57*		
Pseudomonas_corrugata	*GCF_001269905.1*	*57*		
Pseudomonas_corrugata	*GCF_001411965.1*	*57*		
Pseudomonas_corrugata	*GCF_001412065.1*	*57*		
Pseudomonas_corrugata	*GCF_001412195.1*	*57*		
Pseudomonas_corrugata_CFBP_5454	*GCF_000522485.1*	*57*		
Pseudomonas_mediterranea	*GCA_900106005.1*	*57*		
Pseudomonas_mediterranea	*GCF_001411985.1*	*57*		
Pseudomonas_mediterranea	*GCF_001412005.1*	*57*		
Pseudomonas_mediterranea	*GCF_001412045.1*	*57*		
Pseudomonas_mediterranea_CFBP_5447	*GCA_000774145.1*	*57*		
Pseudomonas_sp._7SR1	*GCA_900156465.1*	*57*		
Pseudomonas_sp._NFACC09-4	*GCA_900119215.1*	*57*		
Pseudomonas_sp._NFACC43	*GCA_900119645.1*	*57*		
Pseudomonas_sp._NFACC47-1	*GCA_900119425.1*	*57*		
Pseudomonas_sp._NFACC49-2	*GCA_900119655.1*	*57*		
Pseudomonas_sp._Pf153	*GCF_001269775.1*	*57*		
Pseudomonas_sp._SHC52	*GCA_000801235.1*	*58*	x	
Pseudomonas_fluorescens	*GCF_001648775.1*	*59*	x	
Pseudomonas_fluorescens	*GCA_000802985.1*	*59*		
Pseudomonas_fluorescens_NZ011	*GCF_000276585.1*	*59*		
Pseudomonas_flexibilis	*GCA_900155995.1*	*60*	x	
Pseudomonas_flexibilis	*GCA_000806415.1*	*60*		
Pseudomonas_flexibilis	*GCA_900101515.1*	*60*		
Pseudomonas_sp._RL	*GCF_000647775.1*	*60*		
Pseudomonas_fluorescens	*GCF_001020875.1*	*61*	x	
Pseudomonas_fluorescens	*GCA_000827755.2*	*61*		
Pseudomonas_sp._P1.31	*GCF_001269815.1*	*61*		

Pseudomonas_xanthomarina_DSM_18231	GCA_900129835.1	62	x	
Pseudomonas_sp._10B238	GCA_000970615.1	62		
Pseudomonas_sp._MT-1	GCA_000828755.1	62		
Pseudomonas_stutzeri	GCA_001648195.1	62		
Pseudomonas_xanthomarina	GCA_900108535.1	62		
Pseudomonas_sp._StFLB209	GCA_000829415.1	63	x	
Pseudomonas_fluorescens	GCA_000836415.1	64	x	
Pseudomonas_chlororaphis	GCA_001023535.1	64		
Pseudomonas_sp._P97.38	GCF_001269745.1	64		
Pseudomonas_sp._FeS53a	GCA_000931385.1	65	x	
Pseudomonas_putida	GCF_000800615.1	66	x	x
Pseudomonas_donghuensis_HYS	GCF_000259195.1	66		x
Pseudomonas_sp._2(2015)	GCA_000955865.1	66		x
Pseudomonas_sp._P482	GCA_000696345.1	66		x
Pseudomonas_toyotomiensis	GCF_000974625.1	67	x	
Pseudomonas_alcaliphila	GCA_900101755.1	67		
Pseudomonas_alcaliphila	GCF_001653895.1	67		
Pseudomonas_alcaliphila_34	GCF_000319815.1	67		
Pseudomonas_alcaliphila_JAB1	GCA_001941865.1	67		
Pseudomonas_alcaliphila_NBRC_102411	GCF_002091495.1	67		
Pseudomonas_pseudoalcaligenes	GCA_000962895.1	67		
Pseudomonas_sp._Leaf83	GCA_001422075.1	67		
Pseudomonas_sp._NFACC19-2	GCA_900119125.1	67		
Pseudomonas_toyotomiensis	GCA_900115695.1	67		
Pseudomonas_lini	GCA_001238395.1	68	x	
Pseudomonas_fluorescens	GCA_000967945.1	68		
Pseudomonas_fluorescens	GCF_000800625.1	68		
Pseudomonas_lini	GCA_001042905.1	68		
Pseudomonas_sp._C9	GCA_002000165.1	68		
Pseudomonas_sp._QTF5	GCF_000512695.1	68		
Pseudomonas_sp._URMO17WK12:I11	GCA_001511755.1	68		
Pseudomonas_sp._URMO17WK12:I12	GCF_000514395.1	68		
Pseudomonas_taetrolens	GCA_900104825.1	69	x	
Pseudomonas_fluorescens	GCF_001020625.1	69		
Pseudomonas_fluorescens	GCF_001020635.1	69		
Pseudomonas_fluorescens	GCF_001020645.1	69		
Pseudomonas_fluorescens	GCF_001020655.1	69		
Pseudomonas_fluorescens	GCF_001020705.1	69		
Pseudomonas_fluorescens	GCF_001020765.1	69		
Pseudomonas_fluorescens	GCF_001020785.1	69		
Pseudomonas_fluorescens	GCF_001020795.1	69		
Pseudomonas_fluorescens	GCF_001020805.1	69		
Pseudomonas_helleri	GCA_001043025.1	69		
Pseudomonas_helleri	GCA_001043065.1	69		
Pseudomonas_lundensis	GCA_001042985.1	69		
Pseudomonas_lundensis	GCA_900103705.1	69		
Pseudomonas_sp._BS-2016	GCA_001020725.2	69		
Pseudomonas_sp._BS-2016	GCF_001806125.1	69		
Pseudomonas_sp._BS-2016	GCF_001806135.1	69		
Pseudomonas_sp._BS-2016	GCF_001806145.1	69		
Pseudomonas_taetrolens	GCA_001042915.1	69		
Pseudomonas_weihenstephanensis	GCA_001043055.1	70	x	
Pseudomonas_psychrophila_HA-4	GCF_000282975.1	70		
Pseudomonas_sp._DSM_28140	GCA_001042975.1	70		
Pseudomonas_sp._TAA207	GCA_001529285.1	70		

Pseudomonas_sp._TAD18	*GCA_001529305.1*	*70*		
Pseudomonas_aeruginosa	*GCF_001765485.1*	*71*	x	
Pseudomonas_aeruginosa	*GCA_001180425.1*	*71*		
Pseudomonas_fuscovaginae_SE-1	*GCF_000364705.1*	*72*	x	
Pseudomonas_fuscovaginae	*GCA_001293465.1*	*72*		
Pseudomonas_fuscovaginae	*GCA_001293475.1*	*72*		
Pseudomonas_syringae_pv._syringae	*GCA_001293665.1*	*73*	x	
Pseudomonas_meliae	*GCA_001400515.1*	*74*	x	
Pseudomonas_amygdali_pv._dendropanacis	*GCA_001400435.1*	*74*		
Pseudomonas_syringae_pv._lapsa	*GCA_001400495.1*	*75*	x	
Pseudomonas_coronafaciens_pv._oryzae	*GCA_001400895.1*	*76*	x	
Pseudomonas_coronafaciens_pv._atropurpurea	*GCA_001400695.1*	*76*		
Pseudomonas_syringae_pv._rhaphiolepidis	*GCA_001400995.1*	*77*	x	
Pseudomonas_tremae	*GCA_001401155.1*	*78*	x	
Pseudomonas_endophytica	*GCA_001411475.1*	*79*	x	
Pseudomonas_veronii	*GCA_001439695.1*	*80*	x	
Pseudomonas_antarctica	*GCA_001647715.1*	*80*		
Pseudomonas_extremaustralis	*GCA_900102035.1*	*80*		
Pseudomonas_extremaustralis	*GCA_900167635.1*	*80*		
Pseudomonas_fluorescens	*GCA_001708445.1*	*80*		
Pseudomonas_fluorescens	*GCA_001708465.1*	*80*		
Pseudomonas_fluorescens	*GCA_001708485.1*	*80*		
Pseudomonas_fluorescens	*GCA_002022325.1*	*80*		
Pseudomonas_fluorescens	*GCF_000801835.1*	*80*		
Pseudomonas_fluorescens	*GCF_001269845.1*	*80*		
Pseudomonas_fluorescens	*GCF_001444295.1*	*80*		
Pseudomonas_fluorescens_NBRC_14160	*GCF_002091595.1*	*80*		
Pseudomonas_sp._AP19	*GCA_001728855.1*	*80*		
Pseudomonas_sp._TTU2014-080ASC	*GCA_001446935.1*	*81*	x	
Pseudomonas_agarici_NCPPB_2289	*GCF_000280785.1*	*82*	x	
Pseudomonas_agarici	*GCA_001543125.1*	*82*		
Pseudomonas_agarici	*GCA_900109755.1*	*82*		
Pseudomonas_balearica_DSM_6083	*GCA_900103375.1*	*83*	x	
Pseudomonas_balearica	*GCA_001575025.2*	*83*		
Pseudomonas_balearica	*GCA_001575035.2*	*83*		
Pseudomonas_stutzeri	*GCA_001635435.1*	*84*	x	
Pseudomonas_putida	*GCA_001700705.1*	*85*	x	x
Pseudomonas_putida	*GCA_001636055.1*	*85*		x
Pseudomonas_putida	*GCA_001700655.1*	*85*		x
Pseudomonas_putida	*GCA_001700665.1*	*85*		x
Pseudomonas_putida	*GCA_001700695.1*	*85*		x
Pseudomonas_sp._NBRC_111142	*GCF_001320925.1*	*85*		x
Pseudomonas_sp._NBRC_111143	*GCF_001320965.1*	*85*		x
Pseudomonas_putida	*GCA_001941965.1*	*86*	x	x
Pseudomonas_putida	*GCA_001644955.1*	*86*		x
Pseudomonas_sp._GR_6-02	*GCA_001655615.1*	*87*	x	
Pseudomonas_resinovorans	*GCF_002003485.1*	*88*	x	
Pseudomonas_sp._TCU-HL1	*GCA_001708505.1*	*88*		
Pseudomonas_sp._21C1	*GCA_001728865.1*	*89*	x	
Pseudomonas_marincola	*GCF_001940335.1*	*90*	x	
Pseudomonas_sp._J237	*GCA_001749735.1*	*90*		
Pseudomonas_putida_MTCC_5279	*GCF_000411615.1*	*91*	x	x
Pseudomonas_monteilii	*GCF_001753835.1*	*91*		x
Pseudomonas_sp._HMSC08G10	*GCA_001808425.1*	*91*		x
Pseudomonas_aeruginosa	*GCA_001920965.1*	*92*	x	

Pseudomonas_chlororaphis	*GCA_001921865.1*	*93*	x	
Pseudomonas_pachastrellae	*GCA_001989375.1*	*94*	x	
Pseudomonas_sp._MF4836	*GCA_002018875.1*	*95*	x	
Pseudomonas_sp._MF6396	*GCA_002018915.1*	*96*	x	x
Pseudomonas_sp._LPH1	*GCA_002037565.1*	*97*	x	
Pseudomonas_sp._S-6-2	*GCA_002056295.1*	*98*	x	
Pseudomonas_abietaniphila	*GCF_000974525.1*	*99*	x	
Pseudomonas_abietaniphila	*GCA_900100795.1*	*99*		
Pseudomonas_abietaniphila	*GCF_000876035.1*	*99*		
Pseudomonas_sp._Bc-h	*GCA_002080045.1*	*99*		
Pseudomonas_cichorii	*GCA_900104015.1*	*100*	x	
Pseudomonas_fragi_NBRC_3458	*GCF_002091615.1*	*101*	x	
Pseudomonas_fragi	*GCA_900105835.1*	*101*		
Pseudomonas_fragi_B25	*GCF_000250615.1*	*101*		
Pseudomonas_fuscovaginae_UPB0736	*GCF_000251185.1*	*102*	x	
Pseudomonas_fuscovaginae	*GCA_900108595.1*	*102*		
Pseudomonas_hussainii	*GCA_900109735.1*	*103*	x	
Pseudomonas_bauzanensis	*GCA_900114735.1*	*104*	x	
Pseudomonas_bauzanensis	*GCA_900111225.1*	*104*		
Pseudomonas_otitidis	*GCA_900111835.1*	*105*	x	
Pseudomonas_punonensis	*GCA_900142655.1*	*106*	x	
Pseudomonas_putida	*GCA_900167985.1*	*107*	x	x
Pseudomonas_putida	*GCA_900156185.1*	*107*		x
Pseudomonas_sp._A214	*GCA_900156295.1*	*108*	x	
Pseudomonas_sp._S9	*GCF_000222125.1*	*109*	x	
Pseudomonas_thermotolerans_J53	*GCF_000513835.1*	*110*	x	
Pseudomonas_thermotolerans_DSM_14292	*GCF_000364625.1*	*110*		
Pseudomonas_umsongensis_UNC430CL58Col	*GCF_000620285.1*	*111*	x	
Pseudomonas_mandelii_36MFCvi1.1	*GCF_000381285.1*	*111*		
Pseudomonas_sp._35MFCvi1.1	*GCF_000378525.1*	*111*		
Pseudomonas_sp._45MFCol3.1	*GCF_000382025.1*	*111*		
Pseudomonas_umsongensis_20MFCvi1.1	*GCF_000377725.1*	*111*		
Pseudomonas_pelagia_CL-AP6	*GCF_000410875.1*	*112*	x	
Pseudomonas_aeruginosa_PAO1-CipR	*GCF_000414255.1*	*113*	x	
Pseudomonas_caeni_DSM_24390	*GCF_000421765.1*	*114*	x	
Pseudomonas_resinovorans_DSM_21078	*GCF_000423545.1*	*115*	x	
Pseudomonas_azotifigens_DSM_17556	*GCF_000425625.1*	*116*	x	
Pseudomonas_nitroreducens_HBP1	*GCF_000518065.1*	*117*	x	
Pseudomonas_sp._URHB0015	*GCF_000620245.1*	*118*	x	
Pseudomonas_japonica_NBRC_103040_=_DSM_22348	*GCF_000730585.1*	*119*	x	x
Pseudomonas_sp._ML96	*GCF_000761545.1*	*120*	x	
Pseudomonas_aeruginosa	*GCF_001066645.1*	*121*	x	
Pseudomonas_aeruginosa	*GCF_000797145.1*	*121*		
Pseudomonas_rhodesiae	*GCF_000821225.1*	*122*	x	
Pseudomonas_fluorescens	*GCF_000801855.1*	*122*		
Pseudomonas_fluorescens	*GCF_000801915.1*	*122*		
Pseudomonas_sp._ARP3	*GCF_001029595.1*	*122*		
Pseudomonas_sp._CB1	*GCF_000826105.1*	*123*	x	
Pseudomonas_sp._LFM046	*GCF_000949385.1*	*124*	x	
Pseudomonas_aeruginosa	*GCF_001035605.1*	*125*	x	
Pseudomonas_sp._P1.8	*GCF_001269805.1*	*126*	x	
Pseudomonas_monteilii	*GCF_001319945.1*	*127*	x	x
Pseudomonas_sp._NBRC_111119	*GCF_001320125.1*	*128*	x	x
Pseudomonas_sp._NBRC_111135	*GCF_001320705.1*	*129*	x	
Pseudomonas_composti	*GCF_001567565.1*	*130*	x	

Pseudomonas_sp._BMS12	*GCF_001592875.1*	*131*	x
Pseudomonas_fluorescens	*GCF_001679645.1*	*132*	x
Pseudomonas_punonensis	*GCF_001839645.1*	*133*	x
Pseudomonas_hussainii	*GCF_001885685.1*	*134*	x
Pseudomonas_putida	*GCF_001904555.1*	*135*	x
Pseudomonas_flavescens_NBRC_103044	*GCF_002091575.1*	*136*	x
Pseudomonas_indica_NBRC_103045	*GCF_002091635.1*	*137*	x
Pseudomonas_jinjuensis_NBRC_103047	*GCF_002091655.1*	*138*	x
Pseudomonas_migulae_NBRC_103157	*GCF_002091715.1*	*139*	x

a Sampled Genomes as representatives of the genetic diversity of *Pseudomonas*. Selection was done based on sequence identity of 44 highly conserved genes (See S2 Table)

b List of genomes identified as closely related to SVBP6, included in the Phylogenetic tree shown in Fig 2. These organisms were included as part of the *Putida* complex in Gomila et al. 2015.
