# Supplementary material for: Genomic insights into the broad antifungal activity, plant-probiotic properties, and their regulation, in Pseudomonas donghuensis strain SVBP6
Source: PLoS One. 2018 Mar 14;13(3):e0194088. doi: 10.1371/journal.pone.0194088 (PMC5851621; doi:10.1371/journal.pone.0194088)
Supplement: S4 Table — The MIDI profile of SVBP6 was compared with bibliography data of closely related Pseudomonas species. (PDF) [file pone.0194088.s004.pdf]

Fatty acids[a]	Percentage composition in every strain				
	P. putida ATCC 12633	*P. vranovensis* DSM 16006	*P. alkylphenolica* KL28	*P. donghuensis* HYS	*P. donghuensis* SVBP6[b]
8:0 3-OH	–	–	**0.5**	–	–
10:0	0.35	–	–	1.75	3.81
10:0 3-OH	5.14	6.04	7.8	8.33	17.79
11:0 3-OH	–	0.07	–	0.13	–
12:0	2.98	3.61	3.2	1.66	2.24
12:0 2-OH	6.4	4.85	7.1	5.3	5.62
12:0 3-OH	4.92	4.97	7.8	5.94	8.49
12:1 3-OH	0.43	1.04	1.5	2.21	5.80
13:1 at 12-13	–	–	–	–	**0.65**
14:0	0.59	0.89	0.7	0.59	0.82
14:1 w5c	–	–	**0.5**	–	–
15:1 iso G	**0.11**	–	–	–	–
15:0 iso	**0.2**	–	–	–	–
16:0	26.64	27.1	27.8	27.53	18.86
16:0 3-OH	0.17	0.24	–	–	–
16:1 w5c	–	**0.1**	–	–	–
17:0	0.16	0.14	–	–	–
17:1 w8c	0.15	0.14	–	–	–
17:1 anteiso w9c	–	**0.08**	–	–	–
17:0 cyclo	9.0	5.64	6.0	11.29	9.19
17:1 iso w5c	–	–	–	**0.07**	–
18:0	0.33	0.19	–	0.47	0.46
18:1 w9c	–	–	–	**0.49**	–
19:0 iso	–	–	–	**0.11**	–
19:0 cyclo w8c	0.36	–	–	0.62	1.66
19:0 10-methyl	0.27	0.3	–	–	–
**Summed Features**					
1 (13:0 3-OH and/or iso-15:1 H)	–	–	–	0.42	2.09
2 (C12:0 aldehyde)	–	0.1	–	0.36	0.88
3 (C16:1 w6c and/or C16:1 w7c)	27.57	31.64	26.7	21.34	12.00
5 (anteiso-18:0 and/or 18:2 w6,9c)	0.23	–	–	–	0.54
8 (18:1 w7c or C18:1 w6c)	13.99	12.86	10.5	11.4	8.74
**Total (%)**	**99.99**	**100.00**	**100.1**	**100.01**	**99.63**

[a]Unique representatives of each species are grey-colored. Typical fatty acids of the *Pseudomonas* genus are present in all the species (10:0 3-OH, 12:0, 12:0 2-OH)

[b]SVBP6 values are an average of 5 individual experiments, and we selected those fatty acids that were present in 3 or more assays. SVBP6 strain profile showed 5 differences with that of type strain *P. donghuensis* HYS, those grey-colored in each species column and the Summed feature 5 present in SVBP6 but not in HYS strains.
