# Supplementary material for: Genomic insights into the broad antifungal activity, plant-probiotic properties, and their regulation, in Pseudomonas donghuensis strain SVBP6
Source: PLoS One. 2018 Mar 14;13(3):e0194088. doi: 10.1371/journal.pone.0194088 (PMC5851621; doi:10.1371/journal.pone.0194088)
Supplement: S5 Table — Classification of the COGs by functional categories with one-letter abbreviations for the functional categories was based in the COG database [152]. (PDF) [file pone.0194088.s005.pdf]

Code	Value	Percentage	Description
A	187	3.6	RNA processing and modification
B	0	0,0	Chromatin Structure and dynamics
C	155	3.0	Energy production and conversion
D	33	0.6	Cell cycle control, mitosis and meiosis
E	699	13.3	Amino Acid metabolism and transport
F	124	2.4	Nucleotide metabolism and transport
G	423	8.1	Carbohydrate metabolism and transport
H	363	6.9	Coenzyme metabolism and transport
I	196	3.7	Lipid metabolism and transport
J	146	2.8	Translation, ribosomal structure and biogenesis
K	26	0.5	Transcription
L	85	1.6	Replication, recombination and repair
M	199	3.8	Cell wall/membrane/envelop biogenesis
N	126	2.4	Cell motility
O	100	1.9	Post-translational modification, protein turnover, chaperone functions
P	321	6.1	Inorganic ion transport and metabolism
Q	7	0.1	Secondary metabolites biosynthesis, transport and catabolism
R	143	2.7	General Functional Prediction only
S	887	16.9	Function Unknown
T	123	2.3	Signal Transduction
U	201	3.8	Intracellular trafficing and secretion
V	321	6.1	Defense mechanisms
W	0	0.0	Extracellular structures
Y	0	0.0	Nuclear structure
Z	0	0.0	Cytoskeleton
-	440	8.4	Not in COGs
