# Supplementary material for: Genomic insights into the broad antifungal activity, plant-probiotic properties, and their regulation, in Pseudomonas donghuensis strain SVBP6
Source: PLoS One. 2018 Mar 14;13(3):e0194088. doi: 10.1371/journal.pone.0194088 (PMC5851621; doi:10.1371/journal.pone.0194088)
Supplement: S6 Table — Detailed values of the ANI analysis of the 142 more closely related Pseudomonas genomes that were chosen to perform the analysis (See materials and methods for further explanation). ANI score is a result from a whole genome comparison. Values higher than 97% of similarity among strains are shown in red. P. donghuensis HYS and Pseudomonas sp. P482 showed high similarity values with SVBP6 strain. (PDF) [file pone.0194088.s006.pdf]

GROUP 11														
P._cremoricolorata_GCA_000759535.1		93.56(3.33)	84.40(4.15)	84.54(4.28)	84.64(4.19)	84.39(4.01)	84.42(4.01)	84.43(4.06)	84.46(4.05)	84.34(4.06)	84.36(4.20)	83.68(4.03)	84.07(3.98)	84.40(4.11)
P._cremoricolorata_DSM_17059__NBRC_16634_GCF_00042	93.56(3.33)		84.48(4.15)	84.61(4.22)	84.86(4.08)	84.47(3.99)	84.56(3.97)	84.55(3.97)	84.58(3.97)	84.54(3.96)	84.26(4.00)	83.73(3.95)	84.07(4.02)	84.52(4.06)
P._putida_GCA_001644955.1	84.40(4.15)	84.48(4.15)		91.63(3.42)	89.22(3.94)	85.83(4.23)	85.78(4.26)	85.81(4.24)	85.83(4.26)	85.81(4.23)	85.56(4.20)	84.75(4.14)	85.37(4.18)	86.01(4.08)
P._putida_GCA_001941965.1	84.54(4.28)	84.61(4.22)	91.63(3.42)		89.68(3.96)	86.01(4.38)	86.08(4.35)	86.09(4.35)	86.20(4.34)	86.03(4.35)	85.84(4.32)	85.06(4.23)	85.59(4.23)	86.34(4.31)
P._japonica_NBRC_103040__DSM_22348_GCF_000730585.1	84.64(4.19)	84.86(4.08)	89.22(3.94)	89.68(3.96)		86.14(4.26)	86.15(4.27)	86.20(4.27)	86.19(4.29)	86.19(4.22)	85.88(4.26)	84.92(4.19)	85.64(4.17)	86.42(4.12)
Pseudomonas_sp._P482_GCA_000696345.1	84.39(4.01)	84.47(3.99)	85.83(4.23)	86.01(4.38)	86.14(4.26)		99.51(0.94)	99.55(0.72)	92.59(3.23)	92.83(3.07)	88.26(4.11)	87.32(4.17)	87.86(4.08)	86.80(4.19)
P._donghuensis_HYS_GCF_000259195.1	84.42(4.01)	84.56(3.97)	85.78(4.26)	86.08(4.35)	86.15(4.27)	99.51(0.94)		99.52(0.99)	92.60(3.22)	92.85(3.05)	88.35(4.15)	87.28(4.18)	87.85(4.09)	86.79(4.22)
Pseudomonas_sp._SVBP6	84.43(4.06)	84.55(3.97)	85.81(4.24)	86.09(4.35)	86.20(4.27)	99.55(0.72)	99.52(0.99)		92.59(3.25)	92.91(3.07)	88.26(4.18)	87.27(4.15)	87.88(4.06)	86.73(4.24)
Pseudomonas_sp._2_2015_GCA_000955865.1	84.46(4.05)	84.58(3.97)	85.83(4.26)	86.20(4.34)	86.19(4.29)	92.59(3.23)	92.60(3.22)	92.59(3.25)		93.26(2.97)	88.29(4.13)	87.28(4.20)	87.83(4.04)	86.87(4.22)
P._putida_GCF_000800615.1	84.34(4.06)	84.54(3.96)	85.81(4.23)	86.03(4.35)	86.19(4.22)	92.83(3.07)	92.85(3.05)	92.91(3.07)	93.26(2.97)		88.28(4.17)	87.28(4.20)	87.82(4.07)	86.76(4.22)
P._alkylphenolica_GCA_000746525.1	84.36(4.20)	84.26(4.00)	85.56(4.20)	85.84(4.32)	85.88(4.26)	88.26(4.11)	88.35(4.15)	88.26(4.18)	88.29(4.13)	88.28(4.17)		89.09(3.77)	88.43(3.86)	86.45(4.20)
Pseudomonas_sp._5_GCA_000955815.1	83.68(4.03)	83.73(3.95)	84.75(4.14)	85.06(4.23)	84.92(4.19)	87.32(4.17)	87.28(4.18)	87.27(4.15)	87.28(4.20)	87.28(4.20)	89.09(3.77)		87.57(3.91)	85.61(4.23)
P._vranovensis_DSM_16006_GCF_000425805.1	84.07(3.98)	84.07(4.02)	85.37(4.18)	85.59(4.23)	85.64(4.17)	87.86(4.08)	87.85(4.09)	87.88(4.06)	87.83(4.04)	87.82(4.07)	88.43(3.86)	87.57(3.91)		86.28(4.17)
Pseudomonas_sp._MF6396_GCA_002018915.1	84.40(4.11)	84.52(4.06)	86.01(4.08)	86.34(4.31)	86.42(4.12)	86.80(4.19)	86.79(4.22)	86.73(4.24)	86.87(4.22)	86.76(4.22)	86.45(4.20)	85.61(4.23)	86.28(4.17)	
