# Supplementary figures and images for: Genomic insights into the broad antifungal activity, plant-probiotic properties, and their regulation, in Pseudomonas donghuensis strain SVBP6
Source: PLoS One. 2018 Mar 14;13(3):e0194088. doi: 10.1371/journal.pone.0194088 (PMC5851621; doi:10.1371/journal.pone.0194088)

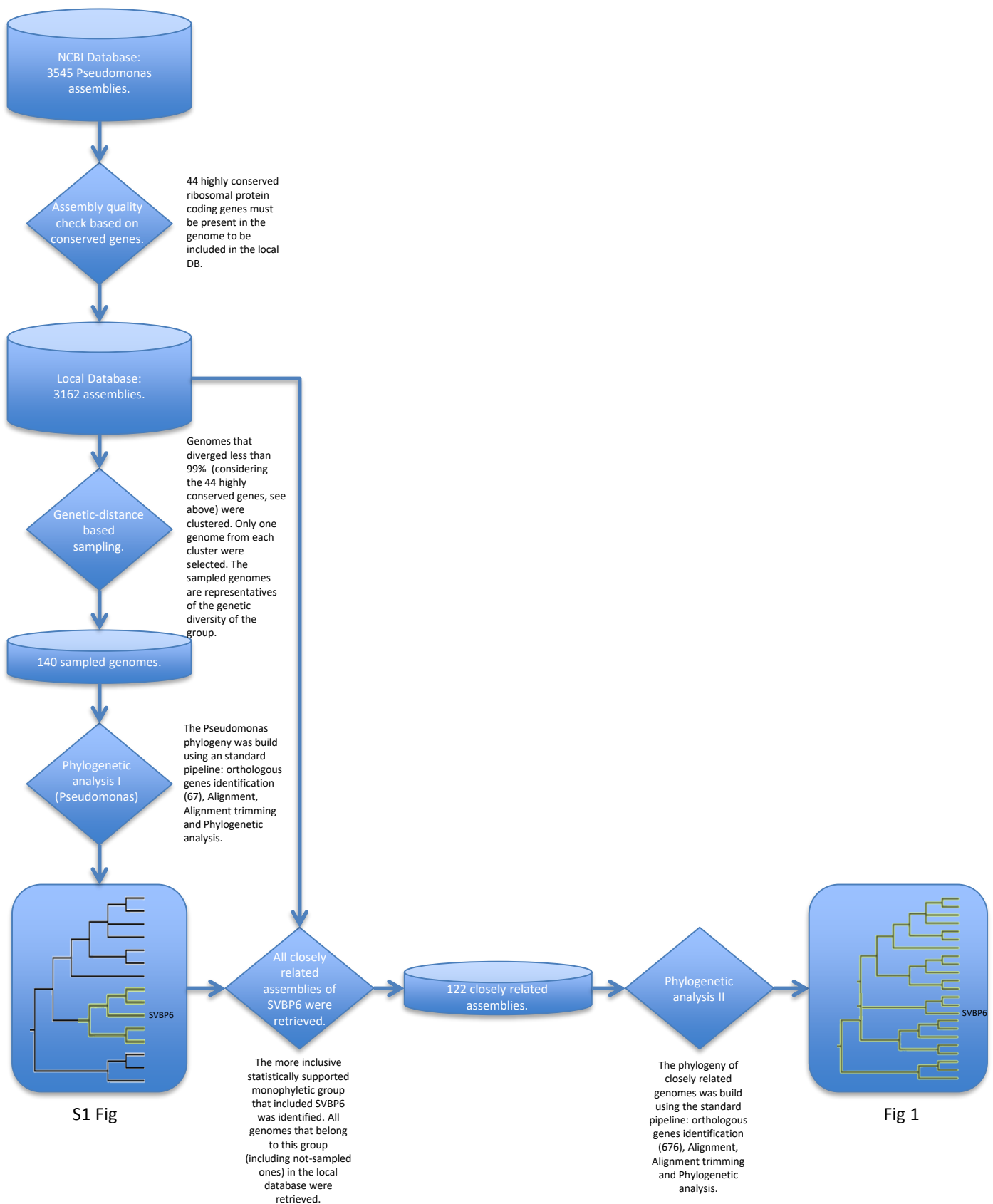

Supplement: S1 Fig — (PDF) [file pone.0194088.s007.pdf]

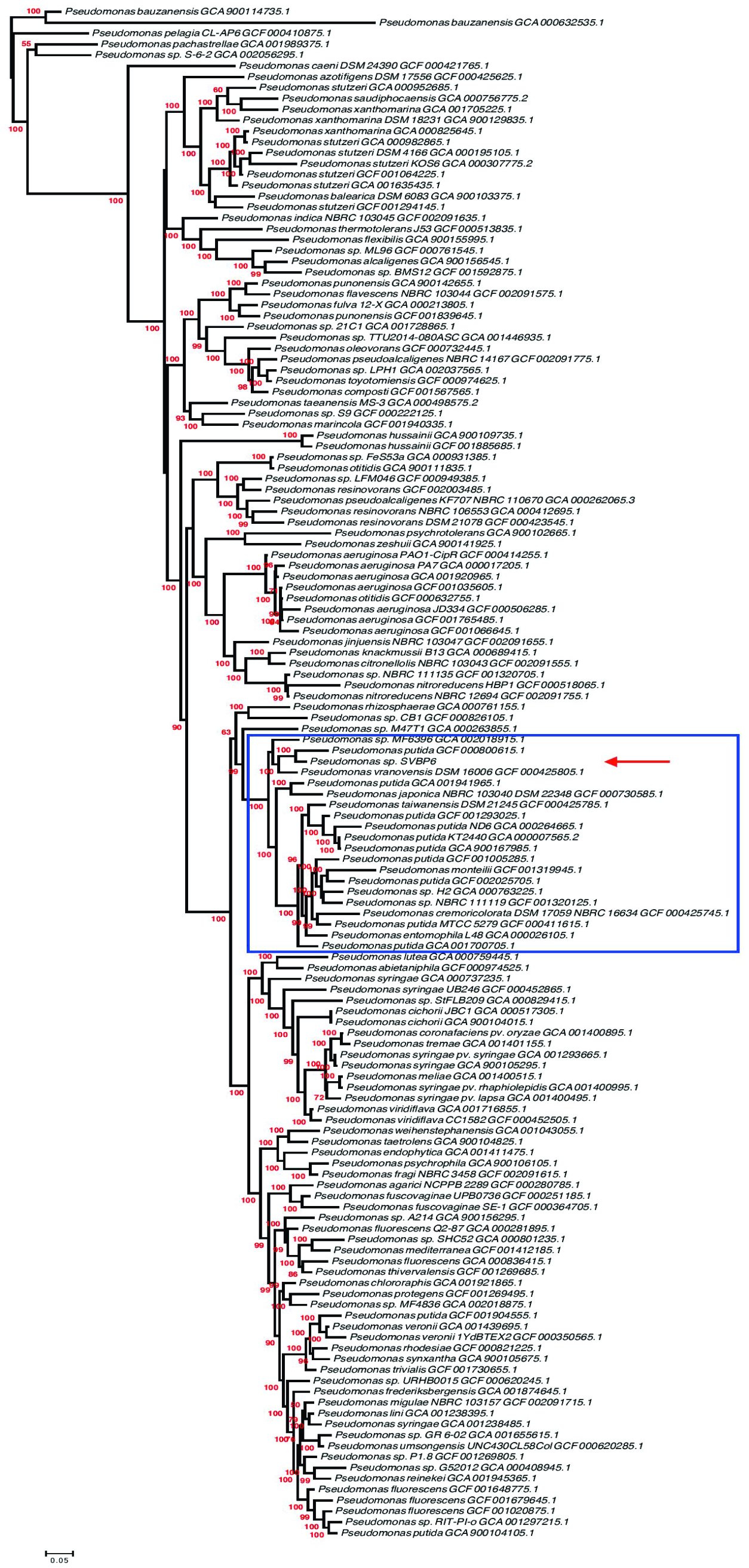

Supplement: S2 Fig — Tree was inferred using an approximate maximum-likelihood method with default parameters by means of FastTree version 2.1. The SH test was used to evaluate branch supports. The 140 genomes other than SVBP6 that were included in this analysis were selected as representatives of the genetic diversity of the genus (see text). Red arrow indicates the position of SVBP6 strain. The monophyletic group selected for further phylogenetic analysis is indicated within a blue box. (TIF) [file pone.0194088.s008.tif]

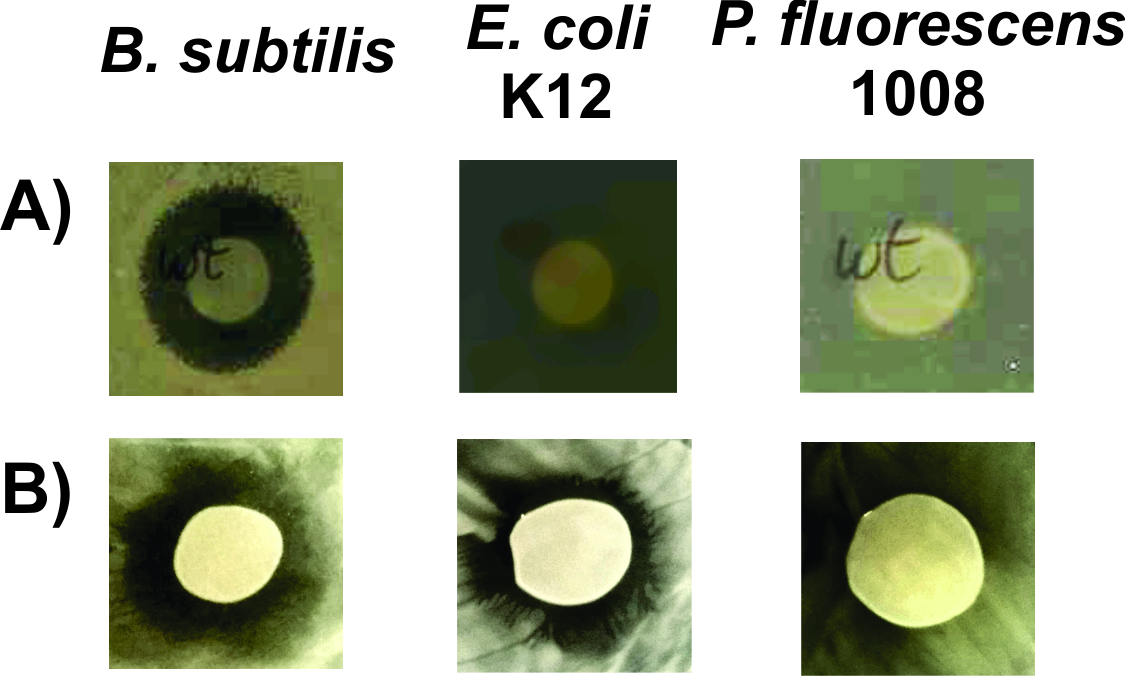

Supplement: S3 Fig — A) Overlaid layer assay, showing a putative release metabolite that could exert the antibacterial activity; B) Co-culture assay, showing a putative secreted metabolite or even a contact-dependent strategy, as T6SS. (TIF) [file pone.0194088.s009.tif]
